# Nipbl Interacts with Zfp609 and the Integrator Complex to Regulate Cortical Neuron Migration

**DOI:** 10.1016/j.neuron.2016.11.047

**Published:** 2017-01-18

**Authors:** Debbie L.C. van den Berg, Roberta Azzarelli, Koji Oishi, Ben Martynoga, Noelia Urbán, Dick H.W. Dekkers, Jeroen A. Demmers, François Guillemot

**Affiliations:** 1 The Francis Crick Institute, Mill Hill Laboratory, The Ridgeway, London NW7 1AA, UK; 2 Center for Proteomics, Erasmus MC, Wytemaweg 80, 3015 CN Rotterdam, the Netherlands

**Keywords:** Nipbl, Zfp609, Cornelia de Lange syndrome, Integrator, neuronal migration, transcription, RNA pol2 pausing

## Abstract

Mutations in *NIPBL* are the most frequent cause of Cornelia de Lange syndrome (CdLS), a developmental disorder encompassing several neurological defects, including intellectual disability and seizures. How *NIPBL* mutations affect brain development is not understood. Here we identify Nipbl as a functional interaction partner of the neural transcription factor Zfp609 in brain development. Depletion of Zfp609 or Nipbl from cortical neural progenitors in vivo is detrimental to neuronal migration. Zfp609 and Nipbl overlap at genomic binding sites independently of cohesin and regulate genes that control cortical neuron migration. We find that Zfp609 and Nipbl interact with the Integrator complex, which functions in RNA polymerase 2 pause release. Indeed, Zfp609 and Nipbl co-localize at gene promoters containing paused RNA polymerase 2, and Integrator similarly regulates neuronal migration. Our data provide a rationale and mechanistic insights for the role of Nipbl in the neurological defects associated with CdLS.

## Highlights

• Nipbl interacts with the transcription factor Zfp609 and the Integrator complex
• Nipbl, Zfp609, and Integrator are required for cortical neuron migration
• Nipbl, Zfp609, and Integrator co-occupy genomic binding sites independently of cohesin
• Nipbl, Zfp609, and Integrator directly regulate neuronal migration genes

## Teaser

*NIPBL* mutations cause Cornelia de Lange syndrome, but Nipbl function in brain development is not well understood. Van den Berg et al. show that Nipbl interacts with Zfp609 and the Integrator complex to transcriptionally regulate cortical neuron migration.

## Introduction

The cerebral cortex, responsible for higher cognitive function, is generated from a pool of progenitor cells that will give rise to the neuronal and glial lineages of the adult brain. Unperturbed migration of newly born neurons across the expanding cortex to their final destination in specific cortical layers ensures accurate connectivity and neuronal circuit formation. Cell-intrinsic transcription factors play key roles in orchestrating the underlying molecular processes, as was recently shown for the proneural transcription factors Neurog2 and Ascl1 (Heng et al., 2008, Pacary et al., 2011). Developmental disturbance of neuronal migration affects shaping of the neuronal network and has been linked to a variety of neurological disorders, including epilepsy, schizophrenia, autism spectrum disorder (ASD), and intellectual disability (Guerrini and Parrini, 2010, Muraki and Tanigaki, 2015, Reiner et al., 2016, Verrotti et al., 2010).

Cornelia de Lange syndrome (CdLS) is one particular example of a developmental disorder highlighted by neurological defects, including seizures and intellectual disability. Other characteristics include facial dysmorphism, growth retardation, and upper limb defects. Heterozygous mutations in the cohesin loading factor Nipped-B-like (*NIPBL*) have been identified in 50%–60% of cases and are associated with a more severe clinical presentation, while mutations in cohesin complex subunits *SMC1A*, *SMC3*, and *RAD21* and in the SMC3-targeting deacetylase *HDAC8* account for a further 10% of mostly mildly affected cases (Braunholz et al., 2015). A genetic cause for the remaining 30% of clinically diagnosed CdLS patients remains unknown.

Despite cohesin complex subunits originally having been identified for their role in sister chromatid cohesion (Michaelis et al., 1997), studies have failed to detect overt chromosome segregation defects in CdLS patients, and instead, deregulated gene expression is thought to be the prime cause of the observed developmental abnormalities (Castronovo et al., 2009, Deardorff et al., 2012, Kawauchi et al., 2009, Liu et al., 2009, Remeseiro et al., 2013). This likely relates to the ability of cohesin to mediate long-range chromosome interactions in *cis*, thereby facilitating enhancer-promoter looping (Kagey et al., 2010, Nativio et al., 2009, Seitan et al., 2013).

How Nipbl acts in gene regulatory networks and developmental pathways in brain development is poorly understood. In this study, we set out to identify new regulators of cortical development by studying a mouse ortholog of *Drosophila scribbler (sbb)*, the single zinc-finger protein Zfp609. We identified Nipbl as a binding partner of Zfp609, which is specifically expressed in neural progenitors in the developing mouse cortex. Zfp609 and Nipbl interact and co-bind genomic regions with the RNA polymerase 2 (RNA pol2)-associated Integrator complex to directly regulate neuronal migration genes. Accordingly, depletion of Zfp609, Nipbl, or Integrator from cortical progenitors in vivo results in neuronal migration defects. Our findings define a Nipbl transcriptional pathway relevant to CdLS.

## Results

### Zfp609 Is Expressed in Neural Progenitors and Regulates Cortical Neuron Migration

**Figure 1:**
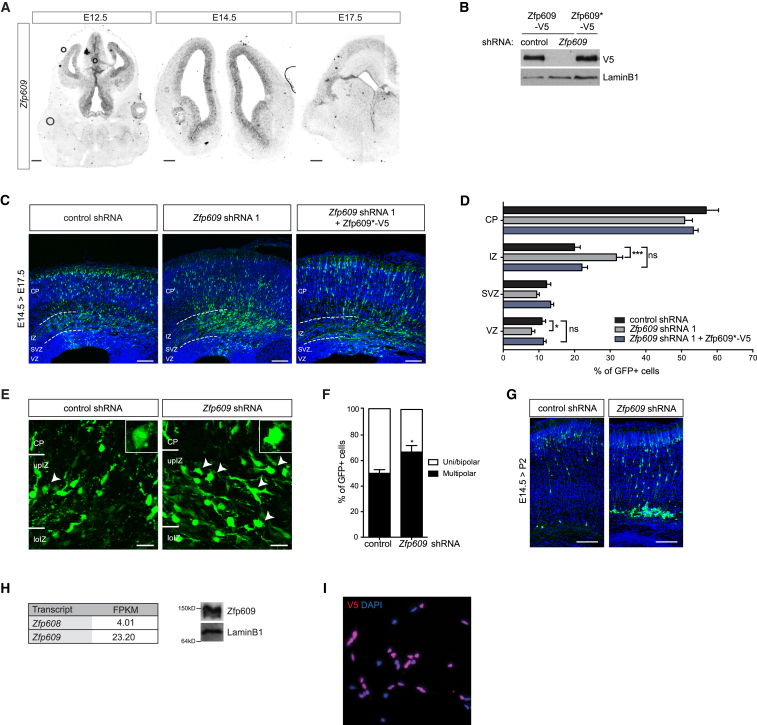
Zfp609 Is Expressed in Neural Progenitors and Regulates Cortical Neuron Migration (A) Composite bright field images of in situ hybridization on cortical cryosections at indicated stages of mouse development. Scale bar represents 200 μm. (B) Western blot with indicated antibodies on HEK293T lysates transiently transfected with wild-type or shRNA-resistant (^∗^) Zfp609-V5 expression constructs and control or Zfp609-targeting shRNA. Lamin B1 was used as a loading control. (C) Cryosections of mouse embryonic brains in utero electroporated with *Zfp609*-targeting shRNAs and Zfp609^∗^-V5 rescue construct, stained with GFP to visualize transfected cells. Ventricular (VZ), subventricular (SVZ), and intermediate zones (IZ) and cortical plate (CP) are indicated. Scale bar represents 100 μm. (D) Quantification of (C) showing percentage of GFP-expressing cells in indicated cortical regions. Error bars represent SEM, ^∗^p < 0.05, ^∗∗∗^p < 0.001; ns, non significant; two-tailed unpaired Student’s t test, n = 7. (E) Representative images showing morphology of electroporated neurons at E17.5 near the border between IZ and CP. Arrowheads point to multipolar cells; higher magnification in inset. Scale bar represents 20 μm. (F) Quantification of cell morphology in upper IZ. Error bars represent SEM, ^∗^p < 0.05, two-tailed unpaired Student’s t test, n = 7 (control shRNA) and 8 (*Zfp609* shRNA). (G) Representative images of cryosections of electroporated mouse embryonic brains at postnatal day 2, stained with GFP antibody. Scale bar represents 100 μm. (H) Normalized expression levels in fragments per kilobase of exon per million mapped reads (FPKM) of *Zfp608* and *Zfp609* transcripts in NSCs. Western blot analysis of NSC lysate with Zfp609 antibody. Lamin B1 was used as a loading control. (I) Immunocytochemistry with V5 antibody on NSCs showing nuclear localization of ectopically expressed Zfp609-V5.

Zfp608 and Zfp609 are vertebrate homologs of *Drosophila* scribbler, a single zinc-finger protein that is highly expressed in the larval CNS, where it is proposed to act as a transcription factor (Haecker et al., 2007, Yang et al., 2000). *Zfp608* is specifically expressed in the mouse forebrain subventricular (SVZ) and intermediate zone (IZ) at embryonic day (E)14.5 (Ayoub et al., 2011). To delineate the expression domain of *Zfp609*, we performed in situ hybridization on brain sections at different developmental stages (Figures 1A and S1A, available online). *Zfp609* transcripts are enriched in and subsequently become restricted to the progenitor population as cortical neurogenesis peaks at E14.5. The absence of *Zfp609* transcripts from cells in the SVZ/IZ at this stage could be attributed to direct repression by Zfp608, as occurs in developing thymocytes (Reed et al., 2013). At later stages of development, *Zfp609* expression is detected in the neurons of the cortical plate (CP) and in stem cells near the ventricular surface.

Because *scribbler* mutations affect axon targeting and larval locomotion (Rao et al., 2000, Suster et al., 2004, Yang et al., 2000), we decided to assess the role of its vertebrate homologs in brain development. Based on their expression pattern and the assumption that any disruption to the progenitor population would affect downstream lineages, we decided to focus our initial analysis on Zfp609. To address the importance of *Zfp609* expression in mouse neural progenitor cells (NPCs) in vivo, we electroporated short hairpin RNA (shRNA) constructs into E14.5 mouse embryonic brains (Tabata and Nakajima, 2001). We designed two independent shRNAs that efficiently deplete Zfp609 at the transcript and protein level (Figures 1B and S1B). Each shRNA construct was injected along with a GFP expression vector into the lateral ventricles of E14.5 mouse embryos and transduced into NPCs near the ventricular surface by a series of electric pulses. We first analyzed the effect of Zfp609 depletion on progenitor proliferation by labeling dividing cells by EdU incorporation at E15.5. The fraction of transduced cells that had exited the cell cycle 24 hr later, identified as labeled by EdU and negative for Ki67, did not significantly differ between the two populations (Figures S1C and S1D).

At E14.5, NPCs give rise to upper-layer cortical neurons and, consistent with this, the majority of control shRNA transduced neurons were found in superficial positions in the CP at E17.5 (Figures 1C and 1D). In contrast, the neuronal progeny of Zfp609-depleted NPCs had an abnormal multipolar morphology and accumulated in the IZ, a phenotype that was confirmed by an independent *Zfp609*-targeting shRNA (Figures 1C–1F, S1E, and S1F). Zfp609-deficient neurons that reached the CP acquired a bipolar morphology, and neither apical dendrites nor axonal length or projection toward the midline were affected (Figures S1G–S1J). Strikingly, heterotopic cell clusters were observed in the white matter of postnatal Zfp609 knockdown (KD) mice (Zfp609 KD, 2/3 mice; control, 0/3 mice; Figure 1G). To further rule out that the aberrant neuronal positioning is due to off-target effects of the shRNAs, we generated an shRNA-resistant Zfp609 construct harboring three silent mutations, designated Zfp609^∗^ (Figure 1B). Co-electroporation of Zfp609^∗^ fully rescued the IZ accumulation observed for Zfp609-depleted cells (Figures 1C and 1D). Taken together, these data suggest that Zfp609 plays a crucial role in the regulation of cortical neuron migration, which cannot be compensated for by Zfp608.

Neural progenitors can be adherently cultured in vitro and form a valuable model system to study molecular mechanisms of neural stem cell (NSC) identity and differentiation, as their limitless expansion enables generation of sufficient material for proteomics studies and other genome-wide approaches. To explore whether they could be used as a tool to study Zfp609 function, we analyzed Zfp609 transcript and protein levels in embryonic stem cell (ESC)-derived NSCs (Conti et al., 2005). Consistent with the in vivo expression pattern, RNA sequencing (RNA-seq) data showed preferential expression of *Zfp609* over *Zfp608* (Figure 1H). We generated an antibody that specifically recognizes Zfp609 (Figures S1K and S1L) and could detect Zfp609 protein in NSC lysates (Figure 1H). Immunocytochemistry on NSCs expressing V5-tagged Zfp609 showed an exclusively nuclear localization, fitting with its proposed role as a transcription factor (Figure 1I).

### Nipbl Is an Interaction Partner of Zfp609 and Regulates Neuronal Migration

**Figure 2:**
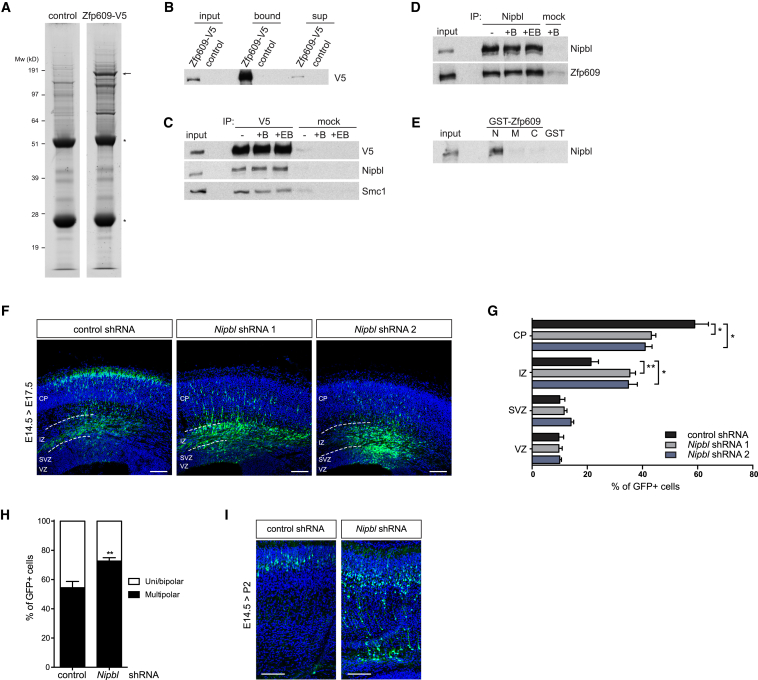
Nipbl Interacts with Zfp609 and Regulates Neuronal Migration (A) Colloidal Coomassie-stained SDS-PAA gel of Zfp609-V5 and control purification. Zfp609-V5 band is indicated by an arrow. Bands representing antibody heavy and light chain are indicated by an asterisk. (B) Western blot with V5 antibody on input, supernatant, and bound fractions of V5 affinity purification. (C) Western blot with indicated antibodies on V5 immunoprecipitates from Zfp609-V5-expressing NSCs. Benzonase (B) or ethidium bromide (EB) was added as indicated. Normal mouse IgG was used as control. (D) Western blot with indicated antibodies on Nipbl immunoprecipitates. Benzonase or ethidium bromide was added as indicated. Normal mouse IgG was used as control. (E) Western blot analysis with Nipbl antibody on GST pull-down fractions from NSC nuclear extract using GST-Zfp609 N-terminal (N), middle (M), and C-terminal (C) fragments or GST control. (F) Cryosections of mouse embryonic brains in utero electroporated with indicated *Nipbl*-targeting shRNAs, stained with GFP to visualize transfected cells. Ventricular (VZ), subventricular (SVZ), and intermediate zones (IZ) and cortical plate (CP) are indicated. Scale bar represents 100 μm. (G) Quantification of (F) showing percentage of GFP-expressing cells in indicated cortical regions. Error bars represent SEM, ^∗^p < 0.05, ^∗∗^p < 0.01, two-tailed unpaired Student’s t test, n = 4. (H) Quantification of cell morphology in upper IZ. Error bars represent SEM, ^∗∗^p < 0.01, two-tailed unpaired Student’s t test, n = 4 (control, *Nipbl* shRNA 1) and 5 (*Nipbl* shRNA 2). (I) Representative images of cryosections of electroporated mouse embryonic brains at postnatal day 2, stained with GFP antibody. Scale bar represents 100 μm.

To gain insight into the molecular environment of Zfp609, we purified Zfp609 from NSCs and identified its interaction partners by mass spectrometry. Nuclear extracts from NSCs expressing doxycycline-inducible V5-tagged Zfp609 were subjected to V5 affinity purification, and Zfp609-containing protein complexes were separated by SDS-PAA gel electrophoresis (Figure 2A). NSCs not expressing Zfp609-V5 were used as a control and benzonase nuclease was added to eliminate DNA-mediated interactions. Colloidal Coomassie staining of Zfp609-V5 immunoprecipitates showed a prominent band at around 150 kD that reacts with V5 antibody (Figure 2B) and many additional bands not detected in the control purification, probably representing Zfp609-interacting proteins.

Gel lanes of Zfp609-V5 and control purifications were analyzed by mass spectrometry, and interaction partners present in two Zfp609 purifications are listed in Table 1. Mascot scores, emPAI (exponentially modified protein abundance index) scores, a semiquantitative measure (Ishihama et al., 2005), and numbers of identified unique peptides of the replicate samples are shown in Table S1. Interestingly, the cohesin complex, comprised of Smc1a, Smc3, Rad21, and Stag2, and its loading factor Nipbl/Mau2 were highly enriched in Zfp609-V5 fractions. Western blot analysis on Zfp609-V5 immunoprecipitates indeed confirmed co-purification of Smc1 and Nipbl (Figure 2C). Neither benzonase nor ethidium bromide affected this interaction, suggesting it occurs independently of DNA. Furthermore, by antibody immunoprecipitation we could show the interaction of endogenous Nipbl and Zfp609 (Figure 2D). Very little Smc1 was detected in these Nipbl immunoprecipitations, indicating that soluble cohesin at endogenous levels is a substoichiometric interactor of Nipbl and Zfp609 (Figure S2A). Finally, to identify the protein domains involved in a direct interaction between Zfp609 and Nipbl, we expressed GST-fusion proteins representing partially overlapping domains of Zfp609 in bacteria (Figures S2B and S2C). GST pull-downs on NSC nuclear extract mapped the interaction with Nipbl to the N-terminal part of Zfp609, which also includes the most highly conserved region. The C2H2 zinc-finger domain by itself was not sufficient for Nipbl binding.

Analogous to *Zfp609*, *Nipbl* transcripts are enriched in the ventricular zone at E14.5 (Figure S2D). Our identified direct physical association between Zfp609 and Nipbl/cohesin may suggest they act together in the cell and therefore mediate the same phenotype. Accordingly, we assessed the effect of Nipbl depletion on neuronal migration in E14.5 NPCs. Each of two independent shRNA constructs that resulted in over 50% reduction in *Nipbl* transcript levels caused significant accumulation of targeted cells in the IZ 3 days after electroporation, accompanied by a reduction of cell numbers in the CP (Figures 2F, 2G, and S2E). Arrested neurons had an atypical multipolar morphology and resulted in white matter heterotopias at postnatal stages (3/5 mice; Figures 2H and 2I). Other aspects of neurogenesis were not notably affected by Nipbl depletion (Figures S1G–S1I). We conclude that Zfp609 and Nipbl physically interact and regulate the same cellular process during mouse forebrain development.

### Zfp609 and Nipbl Co-occupy Active Promoter and Enhancer Regions

**Figure 3:**
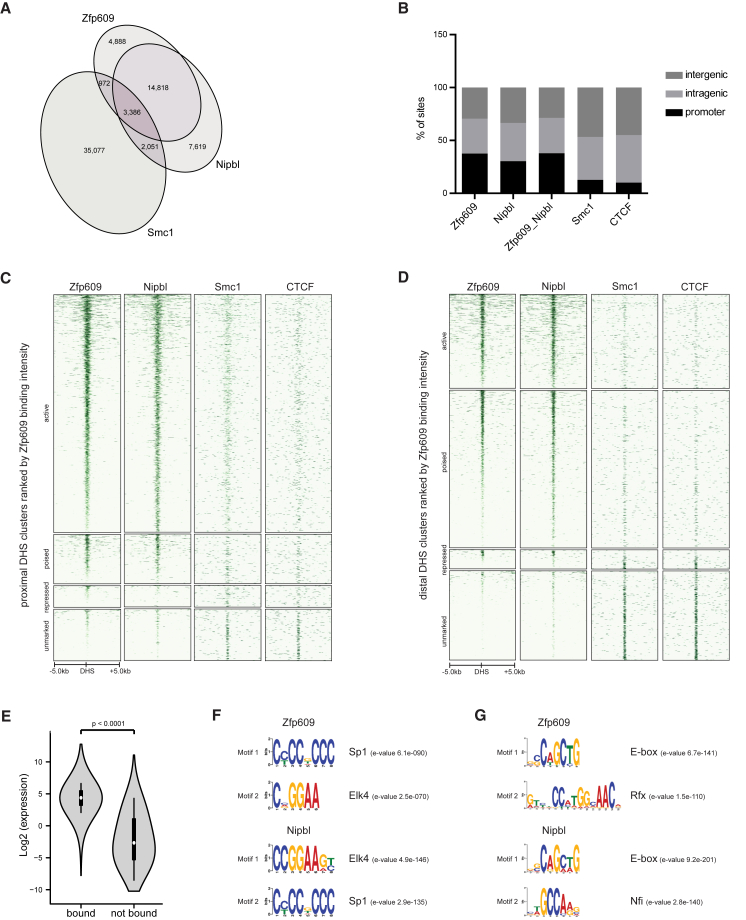
Zfp609 and Nipbl Co-localize to Active Promoter and Enhancer Regions (A) Venn diagram showing overlap of Zfp609, Nipbl, and Smc1 bound regions. (B) Distribution of Zfp609, Nipbl, Zfp609/Nipbl common, Smc1, and CTCF genomic binding sites to promoters (−1 kb to +1 kb)and intra- and intergenic regions. (C) Heatmap of 7,030 active H3K4me3, H3K27ac-marked; 1,498 poised H3K4me1/me2-marked; 690 repressed H3K4me2, H3K27me3-marked; and 1,573 unmarked promoter proximal DNaseI hypersensitive (DHS) sites centered around DHS summits. Regions are ranked by normalized Zfp609 ChIP-seq signal, and mean ChIP-seq counts of indicated factors are plotted. (D) Heatmap of 3,912 active H3K4me1, H3K27ac-marked; 6,487 poised H3K4me1-marked; 866 repressed H3K4me1, H3K27me3-marked; and 3,714 unmarked distal DHSs displaying 10 kb region around DHS summit. Regions are ranked by normalized Zfp609 ChIP-seq signal, and mean ChIP-seq counts of indicated factors are plotted. (E) Violin plot showing distribution of log2 transformed absolute expression values of genes either bound or not bound in their promoter region by Zfp609 and Nipbl. White dot indicates the median and thick black bar represents the interquartile range. p value by Mann-Whitney test is indicated. (F) Top two most significantly enriched motifs in Zfp609 and Nipbl proximal binding peaks. Enrichment values as reported by Centrimo are listed. (G) Top two most significantly enriched motifs in distal Zfp609 and Nipbl peaks. Enrichment values as reported by Centrimo are listed.

To assess if Zfp609 and Nipbl may cause the same phenotype by cooperating in regulating target genes, we first determined genomic binding sites for both factors in cultured NSCs. Chromatin immunoprecipitations of ectopically expressed Zfp609-V5 and endogenous Nipbl (Figures S3A and S3B) were analyzed by high-throughput sequencing of bound DNA (ChIP-seq). Two independent biological replicates correlated well (Pearson r > 0.9) and samples were pooled for further analysis. We identified 24,064 Zfp609 and 27,874 Nipbl genomic binding sites. Zfp609 and Nipbl showed a strikingly high (65%–75%) overlap in binding sites (Figure 3A). Zfp609 binding signal corresponded well to that of Nipbl (Figure S3C).

We reanalyzed published data on genome-wide binding profiles of cohesin subunit Smc1 and CTCF in NSCs (Phillips-Cremins et al., 2013). Previously, cohesin sites in enhancer and promoter regions were demonstrated to also be bound by Nipbl and the Mediator complex in ESCs (Kagey et al., 2010). We found that binding sites of Nipbl and Zfp609 hardly overlap with Smc1, while Smc1 and CTCF binding sites did extensively overlap in NSCs (Figures 3A, S3C, and S3D). A similar discrepancy between the genomic localization of cohesin and its loading factor was recently reported in human mammary epithelial HB2 cells (Zuin et al., 2014). Our results cannot be attributed to differences in antibody epitope or performance, as the antibody was identical to the one used for ChIP-seq in ESCs (Kagey et al., 2010). This suggests that also in NSCs, Nipbl can have a cohesin-independent role in transcription regulation.

Compared to cohesin, CTCF, and other NSC transcription factors (Figure 3B; Mateo et al., 2015), Nipbl and Zfp609 have a preference for promoter regions, with 30%–39% of binding sites mapping in a window from −1 kb to +1 kb around transcription start sites (TSSs). We examined the chromatin landscape surrounding Zfp609 and Nipbl binding sites by profiling their binding intensity to a catalog of NSC regulatory elements compiled based on histone modification patterns surrounding DNaseI hypersensitive sites (DHSs) (Mateo et al., 2015). We found that Nipbl and Zfp609 predominantly localize to active and poised proximal and distal DHS clusters, hallmarked by the presence of H3K27ac and by the absence of H3K27 post-translational modification, respectively (Figures 3C and 3D). Nipbl and Zfp609 promoter-bound genes have an above-average expression level (Figure 3E). In contrast, Smc1 and CTCF binding was mostly detected at DHSs that were not marked by any of the assessed histone modifications (Figures 3C and 3D).

Motif discovery analysis using MEME-ChIP (Machanick and Bailey, 2011) detected enrichment of consensus sites for Sp1 and the Ets family transcription factor Elk4 in proximal DHSs bound by Zfp609 and Nipbl (Figure 3F). These motifs are commonly found in promoter regions (Mateo et al., 2015) and, apart from the bimodal distribution of Elk4 motifs flanking Nipbl sites, are not centrally enriched in Zfp609 or Nipbl peaks (Figure S3E), suggesting that Zfp609 or Nipbl is not targeted to promoter regions by sequence-specific transcription factors. E-box motifs recognized by bHLH transcription factors are highly abundant in active enhancer elements (Mateo et al., 2015) and were significantly enriched at the center of distal Zfp609 and Nipbl peaks (Figures 3G and S3F). In addition, we found a significant central enrichment for nuclear factor I (Nfi) and Rfx motifs. Rfx4 was detected as a Zfp609 co-purifying factor by mass spectrometry (Table 1), and although this approach did not identify bHLH transcription factors, we were able to demonstrate a specific DNA-independent interaction between V5-Ascl1 and FLAG-tagged Zfp609 (Figure S3G). Rfx and bHLH factors therefore constitute candidate-targeting factors for Zfp609 and Nipbl to distal regulatory elements.

### Zfp609 and Nipbl Regulate Neuronal Migration Genes

**Figure 4:**
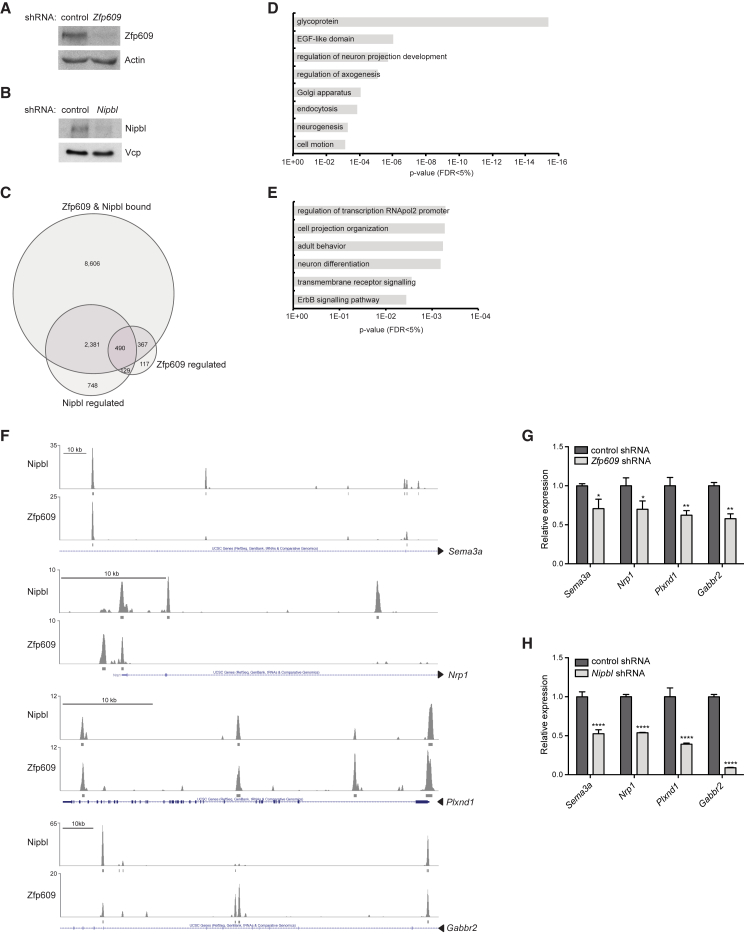
Zfp609 and Nipbl Regulate Neuronal Migration Genes (A) Western blot with Zfp609 antibody on NSC lysates lentivirally transduced with control or *Zfp609*-targeting shRNA. Actin was used as a loading control. (B) Western blot with Nipbl antibody on NSC lysates lentivirally transduced with control or *Nipbl*-targeting shRNA. Vcp was used as a loading control. (C) Venn diagram showing intersection of genes bound in their regulatory region (basal −5 kb to +1 kb plus extension up to 1 Mb, GREAT) and transcriptionally regulated by Zfp609 and Nipbl. (D) GO analysis on genes bound and activated by Zfp609 and Nipbl. DAVID p values are shown, FDR < 5%. (E) GO analysis on genes bound and repressed by Zfp609 and Nipbl. DAVID p values are shown, FDR < 5%. (F) UCSC browser tracks displaying normalized Nipbl and Zfp609 ChIP-seq signal in intragenic and promoter regions of indicated genes. Significant called peaks are indicated on a separate line. (G) qPCR analysis on NSCs lentivirally transduced with control or *Zfp609*-targeting shRNA. Error bars represent SEM, ^∗^p < 0.05, ^∗∗^p < 0.01, unpaired Student’s t test corrected for multiple comparisons using Holm-Sidak method, n = 3. (H) qPCR analysis on NSCs lentivirally transduced with control or *Nipbl*-targeting shRNA. Error bars represent SEM, ^∗∗∗∗^p < 0.0001, unpaired Student’s t test corrected for multiple comparisons using Holm-Sidak method, n = 3.

To identify Nipbl and Zfp609 target genes that could account for the neuronal migration defects observed in vivo, we depleted both factors individually from NSCs by RNAi and identified differentially expressed genes by RNA-seq (Figures 4A, 4B, S4A, and S4B). Nipbl and Zfp609 KD significantly affected the expression of 3,748 and 1,103 genes, respectively. A much higher fraction of genes than would be expected by chance were deregulated in both KD conditions (n = 619, p = 3.10 × 10^−215^, hypergeometric test). Out of these, 83% changed expression in the same direction, suggesting cooperativity between Nipbl and Zfp609 in gene regulation. We subsequently focused our analysis on shared target genes of Nipbl and Zfp609, postulated as bound and regulated by both factors. To associate genes with Nipbl and Zfp609 binding sites, we used GREAT (Genomic Regions Enrichment of Annotations Tool) (McLean et al., 2010), which assigns to each gene a basal regulatory domain from −5 kb to +1 kb, extending up to 1 Mb to the next neighboring basal regulatory domains. Intersection of thus-determined bound genes with deregulated genes resulted in the identification of 490 target genes downstream of both Zfp609 and Nipbl (Figure 4C). A total of 398 target genes were misregulated in parallel, with 244 genes activated and 154 genes repressed by both factors.

Gene ontology (GO) analysis on these two categories of Zfp609/Nipbl target genes revealed enrichment for terms related to cell motion and the extensive cytoskeletal remodeling that occurs during this process (“cell projection organization,” “regulation of neuron projection development,” and “regulation of axogenesis”), in particular among the set of target genes activated by Nipbl and Zfp609 (Figures 4D and 4E). Similar GO terms were enriched among putative Zfp609 target genes that were also deregulated in E13.5 *Nipbl*^+/−^ brains (Figures S4C and S4D) (Kawauchi et al., 2009). We focused in more detail on a subset of genes (i.e., *Sema3a*, *Nrp1*, *Plxnd1*, and *Gabbr2*) whose downregulation is known to cause neuronal migration defects. Sema3a is a secreted chemoattractive guidance molecule present in a descending gradient from the CP that acts through the co-receptor neuropilin-1 (Nrp1) and specific Plexin receptors, including PlexinD1 (Plxnd1), to promote radial migration of cortical neurons (Chen et al., 2008). Disruption of the gradient or downregulation of either Nrp1 or Plxnd1 results in mislocalization of cells to lower cortical regions (VZ/SVZ/IZ). In addition, non-hyperpolarizing signaling through GABA_B_ receptors was shown to be required for cell transition from the IZ to the CP in vitro and in vivo (Behar et al., 2000, Behar et al., 2001, Bony et al., 2013). Indeed, we could detect Zfp609 and Nipbl binding to promoter and intragenic regions of *Sema3a*, *Nrp1*, *Plxnd1*, and *Gabbr2*, and depletion of Nipbl or Zfp609 reduced expression of these targets in NSCs (Figures 4F–4H). We therefore conclude that Zfp609 and Nipbl co-regulate genes required for cortical neuron migration.

### Zfp609 and Nipbl Interact with Integrator to Regulate Migration Genes

**Figure 5:**
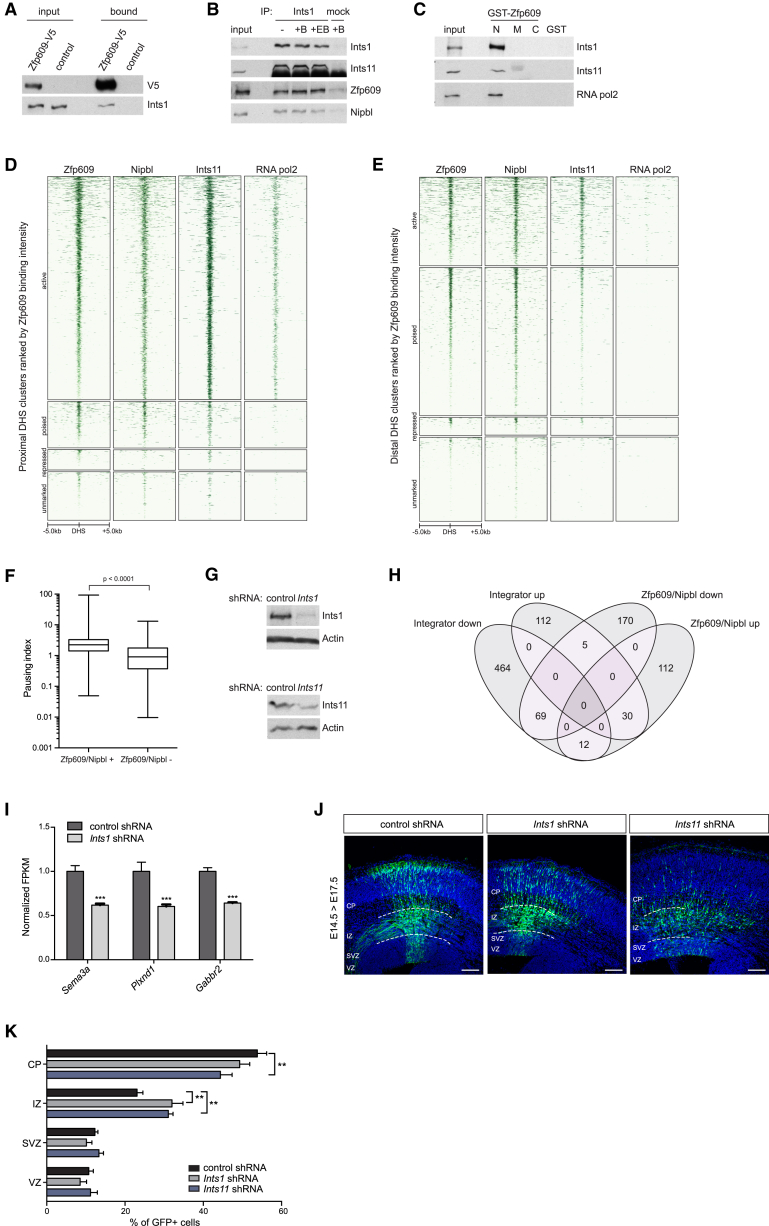
Zfp609 and Nipbl Interact with Integrator to Regulate Cortical Migration (A) Western blot with indicated antibodies of V5 immunoprecipitates on Zfp609-V5-expressing or control NSC nuclear extract. (B) Immunoprecipitation of Ints1 analyzed by western blot with indicated antibodies. Benzonase (B) or ethidium bromide (EB) was added as indicated. Rabbit anti-GFP was used as control. (C) GST pull-down with Zfp609 N-terminal (N), middle (M), and C-terminal (C) fragments or GST control on NSC nuclear extract analyzed by western blot with indicated antibodies. (D) Heatmap of 7,030 active, 1,498 poised, 690 repressed, and 1,573 unmarked promoter proximal DHSs displaying 10 kb around DHS summits. Regions are ranked by normalized Zfp609 ChIP-seq signal, and mean ChIP-seq counts of indicated factors are plotted. (E) Heatmap of 3,912 active, 6,487 poised, 866 repressed, and 3,714 unmarked distal DHSs displaying 10 kb region around DHS summit. Regions are ranked by normalized Zfp609 ChIP-seq signal, and mean ChIP-seq counts of indicated factors are plotted. (F) Boxplot representing distribution of pausing indices of Zfp609 TSS-bound (n = 5,391) versus all other (n = 1,543) expressed (FPKM > 1) genes. Whiskers represent minimum and maximum values. p value by Mann-Whitney test is indicated. (G) Western blot on NSC lysates lentivirally transduced with the indicated shRNAs. Actin was used as a loading control. (H) Venn diagram displaying intersection of deregulated genes in Integrator KD with Zfp609/Nipbl target genes. (I) Normalized expression values from RNA-seq data on control or Ints1-depleted NSCs. Error bars represent SEM, ^∗∗∗^p < 0.001, unpaired Student’s t test corrected for multiple comparisons using Holm-Sidak method, n = 3. (J) Cryosections of mouse embryonic brains in utero electroporated with *Ints1*- and *Ints11 (Cpsf3l)*-targeting shRNAs, stained with GFP to visualize transfected cells. Ventricular (VZ), subventricular (SVZ), and intermediate zones (IZ) and cortical plate (CP) are indicated. Scale bar represents 100 μm. (K) Quantification of (J) showing percentage of GFP-expressing cells in indicated cortical regions. Error bars represent SEM, ^∗∗^p < 0.01, two-tailed unpaired Student’s t test corrected for multiple comparisons using Holm-Sidak method, n = 4 (control) and 6 (*Ints1* and *Ints11* shRNA).

The binding of Zfp609 and Nipbl to active promoter regions suggests they may directly contact the basal transcription machinery to activate transcription. Although we did not consistently detect RNA pol2 subunits in Zfp609 pull-downs, we did find thirteen subunits of the Integrator complex, which associates with the C-terminal domain (CTD) of RNA pol2 (Malovannaya et al., 2010) (Table 1). Specificity of the interaction of Zfp609 with the Ints1 subunit was demonstrated by detection of Ints1 by immunoblotting in the Zfp609-V5 sample and not in the control (Figure 5A).

The interaction of endogenous Zfp609, Nipbl, and Integrator complex was independently verified by their co-immunoprecipitation by an antibody against Ints1. Immunoblotting showed specific co-purification of Zfp609 and Nipbl, independent of DNA (Figure 5B). Western blotting with an antibody against Ints11 (Cpsf3l) was used as a positive control. GST pull-downs mapped the interaction domain with Integrator to the N-terminal region of Zfp609, which also brought down RNA pol2 (Figure 5C).

We determined the genomic distribution of Integrator binding by performing ChIP-seq with an antibody that efficiently brought down chromatin-bound Ints11 (Figure S5A). Three independent replicates correlated well (Pearson r > 0.86) and were pooled for downstream analysis. Consistent with published reports from HeLa cells, we detected widespread binding to promoter and predominantly active enhancer regions, where Integrator strongly co-localizes with Zfp609 and Nipbl (Figures 5D, 5E, and S5C) (Gardini et al., 2014, Lai et al., 2015). For comparison, we included genome-wide ChIP-seq data for RNA pol2, showing its preferential binding to active promoter regions (Figures 5D, 5E, and S5B).

Integrator was recently shown to physically associate with negative elongation factor (NELF) and the DRB-sensitivity inducing factor (DSIF) complex and to affect RNA pol2 pause release at coding genes (Gardini et al., 2014, Skaar et al., 2015, Stadelmayer et al., 2014, Yamamoto et al., 2014). We therefore asked whether paused RNA pol2 also constitutes a key feature of genes containing Zfp609 and Nipbl binding sites in their promoter proximal regions. Indeed, when we compared RNA pol2 pausing indices of Zfp609/Nipbl-bound versus all non-bound expressed genes (fragments per kilobase of transcript per million mapped reads [FPKM] > 1), we could detect significantly increased pausing at Zfp609/Nipbl promoter-bound genes (Figure 5F).

To determine genes co-regulated by Zfp609, Nipbl, and Integrator, we performed RNA-seq analysis on NSCs depleted for Integrator subunits Ints1 and Ints11 by RNAi (Figures 5G and S5D). All of the 142 common putative target genes were deregulated in the same direction by either Ints1 or Ints11 KD, suggesting both factors function agonistically within the Integrator complex (data not shown). Integrator-regulated genes were pooled and compared to the previously classified set of Nipbl and Zfp609 target genes (Figure 5H). In total, 85% of common deregulated genes changed expression in the same direction upon either Zfp609/Nipbl or Integrator KD, suggesting functional cooperativity. Two-thirds of these were downregulated in all KD conditions. Three out of the four genes that we previously implicated in the regulation of cortical neuron migration downstream of Zfp609 and Nipbl, i.e., *Sema3a*, *Plxnd1*, and *Gabbr2*, were downregulated upon Ints1 depletion (Figure 5I). Importantly, compromising Integrator complex function by in utero electroporation of either *Ints1*- or *Ints11*-targeting shRNAs also resulted in an abnormal accumulation of cells in the IZ (Figures 5J and 5K). We therefore conclude that Zfp609 and Nipbl act with the Integrator complex to positively regulate common target genes and thereby promote neuronal migration.

## Discussion

In an unbiased proteomics approach, we identified physical interactions between Zfp609, cohesin, and the cohesin loading factor Nipbl/Mau2. We show that in mice, the two *Drosophila scribbler* homologs *Zfp608* and *Zfp609* are expressed in the embryonic forebrain in a mutually exclusive manner, possibly through direct cross-repression, analogous to what was reported in developing thymocytes (Reed et al., 2013). Indeed, we find that Zfp609 and Nipbl bind to the *Zfp608* promoter in NSCs to silence its transcription. Interestingly, elevated *Zfp608* expression was identified as one of three biomarkers that could accurately distinguish CdLS probands from healthy control individuals (Liu et al., 2009), implicating these factors and their interrelationship in the establishment of CdLS.

We mapped the genomic binding sites of Zfp609 and Nipbl in NSCs to predominantly active promoter and enhancer regions, similar to the genomic distribution of Nipbl in ESCs (Kagey et al., 2010). Contrary to ESCs and despite the significantly higher number of Nipbl peaks identified here, very few Nipbl sites were co-occupied by cohesin. Instead, cohesin almost exclusively localizes to CTCF sites, perhaps as a consequence of cohesin sliding, analogous to reports in yeast (Lengronne et al., 2004). This discrepancy between ESCs and NSCs therefore may reflect a difference in cell-cycle length or cohesin dynamics, which impact the frequency of cohesin complex reloading. Interestingly, a similar disparity in Nipbl and cohesin localization was noted in human breast epithelial cells, which, in combination with differential effects on gene expression, led the authors to propose a cohesin-independent role for Nipbl in gene regulation (Zuin et al., 2014). How Nipbl would act as a transcription factor remained unclear. We now provide evidence that Nipbl contacts the Integrator complex associated with the RNA pol2 CTD, possibly via Zfp609.

Genome-wide ChIP-seq analysis demonstrated that virtually all active and 50% of poised NSC promoters are bound by Integrator, where it often co-localizes with Zfp609/Nipbl. Integrator interacts with the pause-inducing factors NELF and DSIF and occupies promoters containing paused RNA pol2 in HeLa cells (Stadelmayer et al., 2014). Similarly, Zfp609 and Nipbl-bound NSC promoters are characterized by a higher mean RNA pol2 pausing index. RNA pol2 pausing occurs after the first 20–60 nucleotides have been transcribed and provides an additional mode of regulation for nearly 50% of mammalian genes (Kwak and Lis, 2013).

The role of Integrator in the regulation of RNA pol2 pausing appears to be two sided, as it has been shown to be required both for maintenance of pausing and for transition into productive elongation (Gardini et al., 2014, Stadelmayer et al., 2014). Integrator promotes RNA pol2 release at immediate early genes in HeLa cells by recruiting the super elongation complex (SEC), containing AFF4 and the most active form of positive transcription elongation factor P-TEFb (Luo et al., 2012). De novo gain-of-function mutations in *AFF4* were recently identified in three patients with a new syndrome, CHOPS (cognitive development and coarse facies, heart defects, obesity, pulmonary involvement, short stature, and skeletal dysplasia), that displays phenotypic overlap with CdLS (Izumi et al., 2015). We provide biochemical evidence for a link between the most frequently mutated gene in CdLS, *NIPBL*, and the AFF4-containing SEC, implicating the regulation of transcription elongation in the ontogeny of CdLS abnormalities.

We show that depletion of Zfp609, Nipbl, or Integrator in vivo results in aberrant neuronal migration and postulate that the deregulated expression of their target genes involved in semaphorin and GABA signaling is likely to be responsible for this phenotype. *Zfp609* and *Nipbl* transcripts are mostly present in the VZ/SVZ, while the migration arrest occurs in the IZ. This delay could reflect a requirement for Zfp609 and Nipbl to establish and maintain accessibility of genomic binding sites to other regulatory factors. Alternatively, Zfp609 and Nipbl protein expression might be maintained in postmitotic neurons after downregulation of their respective transcripts.

Defects in neuronal migration and the subsequent incorrect neuronal positioning lead to disruption of neural circuit formation and have been causally linked to intellectual disability and seizures, which are both features of CdLS (Liu and Krantz, 2009, Verrotti et al., 2013). Indeed, neuronal heterotopias were reported in autopsy data of CdLS patients (Tekin and Bodurtha, 2015). Abnormal localization of E13.5-born neurons in the IZ was also observed in mice carrying a heterozygous mutation in *Ankrd11*, a chromatin regulator mutated in rare cases of CdLS (Gallagher et al., 2015, Parenti et al., 2016). Furthermore, our data suggest that Zfp609 and Nipbl act through the Integrator complex to contact the basal transcription machinery and regulate gene expression at the level of RNA pol2 pause release. Embryonic brain KD of Phf6 or its interactor Paf1, a recently identified regulator of promoter-proximal RNA pol2 pausing, resulted in aberrant neuronal accumulation in the IZ caused by downregulation of Cspg5, a transmembrane glycoprotein of the neuregulin family (Chen et al., 2015, Zhang et al., 2013). Importantly, mutations in *PHF6* cause Börjeson-Forssman-Lehmann syndrome (BFLS), characterized by moderate-to-severe intellectual disability and seizures (Lower et al., 2002). Together with our data, this suggests a prominent role for the regulation of RNA pol2 pause release in the control of neuronal migration, which ultimately impacts cognitive function.

By studying Nipbl function in neural progenitors in vitro and in vivo, we have generated a deeper understanding of its gene regulatory network and uncovered a role in the control of neuronal migration, which, when perturbed, is likely to contribute to the cognitive impairment of CdLS patients. Our description of the molecular machinery involved in transcription regulation by Nipbl in neural progenitors offers a range of candidates for mutation screening in the 30% of CdLS cases where no causative mutation has been identified.

## Experimental Procedures

### Cell Culture

NS5 NSCs were grown on laminin-coated dishes as previously described (Conti et al., 2005). To generate inducible V5-tagged Zfp609-expressing NSCs, the coding sequence of Zfp609 fused to a C-terminal V5 tag was inserted into lentiviral plasmid Tet-O-FUW-EGFP (kind gift from Marius Wernig, Addgene #30130; Vierbuchen et al., 2010) in place of eGFP. Lentiviral particles were produced by co-transfection with psPax2 and pMD2.G (kind gifts from Didier Trono, Addgene #12260 and #12259) in HEK293T cells and concentrated by ultracentrifugation. NSCs were simultaneously transduced with lentiviruses for Zfp609-V5 and rtTA (kind gift from Rudolf Jaenisch, Addgene #20342; Hockemeyer et al., 2008). Expression of Zfp609-V5 was induced for a minimum of 6 hr by addition of 1 μg/mL doxycycline (Sigma). HEK293T and P19 cells in standard culture conditions were transiently transfected using Lipofectamine 2000 transfection reagent (Invitrogen).

### Antibodies

Zfp609 antibodies were generated in guinea pigs against recombinantly expressed Zfp609 (aa 1–282) fused to GST. Normal mouse IgG (sc-2025), normal rabbit IgG (sc-2027), and antibodies against Lamin B1 (sc-6216 and sc-6217), RNA pol2 (sc-899), and GFP (sc-8334) were obtained from Santa Cruz Biotechnology. Antibodies against Smc1 (A300-055A), Nipbl (A301-779A), Int1 (A300-361A), and Int11 (A301-274A) were obtained from Bethyl Laboratories. Additional antibodies included V5 (R960-25, Invitrogen), Actin (A2066, Sigma), Vcp (ab11433, Abcam), and GFP (4745-1051, AbD Serotec).

### Protein Purification

Control and Zfp609-V5-expressing NSCs were expanded to ten confluent 14 cm diameter dishes (2 × 10^8^ cells) and scraped in ice-cold PBS, and nuclear extracts were prepared (Dignam et al., 1983) and diluted to 100 mM NaCl with C-0 (20 mM HEPES [pH 7.6], 0.2 mM EDTA, 1.5 mM MgCl_2_, and 20% glycerol). Complete EDTA-free protease inhibitors (Roche) were added to all buffers. A total of 40 μL anti-V5 agarose beads (Sigma) were equilibrated in buffer C-100^∗^ (20 mM HEPES [pH 7.6], 100 mM KCl, 0.2 mM EDTA, 1.5 mM MgCl_2_, 0.02% NP-40, and 20% glycerol); blocked in 0.2 mg/mL chicken egg albumin (Sigma), 0.1 mg/mL insulin (Sigma), and 1% fish skin gelatin (Sigma) in C-100^∗^; and added to 1.5 mL nuclear extract in no stick microtubes (Alpha Laboratories) for 3 hr at 4°C in the presence of 225 U Benzonase (Novagen). Beads were washed five times for 5 min with C-100^∗^ and boiled in 30 μL SDS loading buffer. Eluted proteins separated by polyacrylamide gel electrophoresis were stained with Colloidal Coomassie (Invitrogen), and entire gel lanes were analyzed by mass spectrometry as previously described (van den Berg et al., 2010). Criteria for inclusion in Table 1 are presence in both purifications with a Mascot score of at least 50 and both Mascot and emPAI scores at least 3-fold enriched over the corresponding control purification. Cytoskeletal and cytoplasmic proteins were removed from the analysis. For small-scale immunoprecipitations, 2.5 μg antibody, 25 μL protein A or protein G dynabeads, and 200 μL nuclear extract were used. Normal mouse IgG, rabbit IgG, or anti-GFP antibody served as control, and 25 U Benzonase or 25 μg/mL ethidium bromide were added where indicated.

### GST Pull-Down

Zfp609 fragments were cloned into pGEX-2TK. GST-fusion proteins and GST were expressed in BL21-CodonPlus (DE3)-RP competent cells, and GST pull-downs were performed as previously described (van den Berg et al., 2010).

### RNAi and RNA-Seq

Short hairpin sequences (Table S3) were cloned into pSuper (Oligoengine) for transient transfections and in utero electroporation purposes. For RNAi in NSCs, short hairpin sequences were cloned from pENTR/pSUPER+ into pLentiX1 (kind gifts from Eric Campeau, Addgene #17338 and #17297; Campeau et al., 2009). Lentiviral particles were produced in HEK293T cells, concentrated by ultracentrifugation, and used to infect NSCs. Transduced NSCs were selected for 48 hr with 0.5 μg/mL puromycin starting 24 hr after transduction, and RNA was extracted using Trizol reagent (Invitrogen) and purified on RNeasy columns (QIAGEN). Sequencing libraries were prepared according to the TruSeq RNA Sample Preparation v2 kit (Illumina) and sequenced with the HiSeq 2000 (Illumina). Reads were aligned to the mm9 mouse genome with TopHat, and differentially expressed genes listed in Table S2 were identified with Cuffdiff using default parameters (Trapnell et al., 2012). Primer sequences (Table S4) and Taqman probes used for validation by qPCR are listed in the Supplemental Experimental Procedures. DAVID functional clustering webtool (Huang et al., 2009) was used for GO analysis setting a false discovery rate (FDR) < 5%.

### ChIP-Seq Analysis and Data Visualization

For V5, Nipbl, and Ints11 ChIP, NSCs suspended in PBS were crosslinked sequentially for 45 min with 2 mM disuccinimidyl glutarate (DSG) and for 10 min with 1% formaldehyde. Reactions were quenched with 125 mM glycine, chromatin was prepared, and ChIP performed as described (Boyer et al., 2005). RNA pol2 ChIP was performed on formaldehyde-crosslinked chromatin as described (Rahl et al., 2010). Sequencing libraries were prepared from 2–10 ng ChIP or input control DNA according to Illumina standard ChIP-Seq Sample Prep kit and sequenced with a Genome Analyzer IIx or HiSeq 2000 (Illumina). Reads were aligned to the mm9 mouse genome using Bowtie 2 (Langmead and Salzberg, 2012) with default parameters. Aligned reads were subsampled with custom python scripts (Mateo et al., 2015) and peaks called with MACS version 2 (Zhang et al., 2008) setting shift size to 90 and q value to 0.05. ChIP bedGraphs uploaded to UCSC Genome browser mm9 were normalized to control and converted to bigWig using MACS. Venn diagrams were generated using Biovenn (Hulsen et al., 2008) and eulerAPE (Micallef and Rodgers, 2014). Heatmaps were generated from MACS q value bigWig files using the deepTools package (Ramírez et al., 2014). Mean scores of 100 bp bins 10 kb around the peak or DHS summit were displayed. Motif discovery was performed with MEME-ChIP (Machanick and Bailey, 2011) using 400 bases around the peak summit as input. RNA pol2 pausing indices were calculated as the ratio of reads in the TSS proximal region (−250 to +250 bp) over reads in the gene body (+500 bp to +2,500 bp). Genes with no reads in either promoter or gene body region were excluded from the analysis. Primer sequences used for ChIP-qPCR analysis are listed in the Supplemental Experimental Procedures.

### In Utero Electroporation

Mice were housed, bred, and treated according to guidelines approved by the Home Office under the Animal (Scientific Procedures) Act 1986. Experiments were approved by the Crick Animal Welfare and Ethical Review Body and the Home Office (project license 707644).In utero electroporation was performed essentially as described (Azzarelli et al., 2014). Briefly, 1 μg endofree plasmid preparations of pSuper shRNA constructs, pcDNA expression vector, and pCA-b-EGFPm5 silencer3 containing 0.05% Fast Green dye (Sigma) were injected into the lateral ventricle of E14.5 embryos. Five 30 V electric pulses were applied at 1 s intervals across the uterine wall using a 5 mm platinum electrode. Three days after electroporation, embryonic brains were fixed for 1 hr in 4% paraformaldehyde, cryo-protected in 30% sucrose, and embedded in OCT compound (VWR) containing 30% sucrose. Cryosections (14 μm) were stained with anti-GFP (AbD Serotec) and Alexa 488 conjugated secondary antibody and imaged with a confocal microscope. The onset of glia-guided locomotion was used to define the border between IZ and CP (Heng et al., 2010). At least 250 GFP-labeled cells per brain, irrespective of GFP level, were counted blindly; at least four brains were analyzed per condition. The numbers of uni/bipolar (i.e., one to two primary processes) and multipolar (i.e., >two primary processes) were quantified in the upper IZ (n ≥ 20) and CP (n ≥ 70); at least three brains were analyzed per condition.

### In Situ Hybridization

Cryosections were post-fixed in 4% paraformaldehyde, treated for 10 min with 100 mM acetylated triethanolamine (pH 8.0), and pre-hybridized in hybridization buffer (50% formamide, 5× SSC, 5% SDS, and 1 mg/mL yeast tRNA) for 1 hr at 70°C. The Zfp609 probe template was PCR amplified from NSC cDNA (primer sequences in Table S5), in vitro transcribed in the presence of DIG RNA labeling mix (Roche), purified with RNeasy kit (QIAGEN), denatured, and hybridized to cryosections in hybridization buffer overnight at 70°C. Bound probes were detected with an alkaline phosphatase-conjugated anti-DIG antibody (Roche) and NBT/BCIP substrate (Sigma).

## Author Contributions

Conceptualization, D.L.C.v.d.B.; Investigation, D.L.C.v.d.B., R.A., K.O., D.H.W.D., and J.A.D.; Resources, N.U. and B.M.; Writing – Original Draft, D.L.C.v.d.B.; Writing – Review & Editing, D.L.C.v.d.B. and F.G.; Funding Acquisition, D.L.C.v.d.B. and F.G.

## Figures and Tables

**Table 1:** Zfp609-Interacting Proteins as Identified by Mass Spectrometry

Protein Name	Accession Number	Mascot^a^	emPAI^b^	Unique Peptides^c^
Zfp609	UniProt: Q8BZ47	4,314	19.29	71

**Cohesin Complex**				

Nipbl	UniProt: Q6KCD5	2,955	1.57	63
Smc3	UniProt: Q9CW03	2,078	3.66	41
Smc1a	UniProt: Q9CU62	2,048	3.81	44
Stag2	UniProt: A2AFF6	760	0.65	16
Rad21	UniProt: Q61550	732	1.66	17
Mau2	UniProt: Q9D2X5	406	0.86	9

**Integrator Complex**				

Ints1	UniProt: K3W4P2	2,910	2.28	63
Ints6	UniProt: Q6PCM2	1,834	3.99	36
Ints3	UniProt: Q7TPD0	1,714	2.44	32
Ints7	UniProt: Q7TQK1	1,522	2.28	27
Asun	UniProt: Q8QZV7	1,451	5.81	29
Ints5	UniProt: Q8CHT3	993	1.22	19
Ints2	UniProt: Q80UK8	992	0.83	19
Cpsf3l	UniProt: Q9CWS4	869	2.34	19
Vwa9	UniProt: Q8R3P6	712	1.60	14
Ints8	UniProt: Q80V86	746	0.90	17
Ints9	UniProt: Q8K114	579	1.36	15
Ints12	UniProt: Q9D168	575	1.68	11
Nabp2	UniProt: E9Q199	75	0.25	2

**Transcription Factors**				

Rfx4	UniProt: Q7TNK1	760	1.30	18
Zbtb20	UniProt: Q8K0L9	622	0.77	11

**Other**				

Maged1	UniProt: Q9QYH6	824	1.10	16
Hspa2	UniProt: P17156	771	1.40	14
Dnaja2	UniProt: Q9QYJ0	312	0.73	7
Stub1	UniProt: Q9WUD1	287	1.30	8
Akap8l	UniProt: Q9R0L7	235	0.30	5
Bag5	UniProt: Q8CI32	205	0.50	6
Cnp	UniProt: P16330	164	0.34	4
Setx	UniProt: A2AKX3	120	0.04	3
Mlf2	UniProt: Q99KX1	108	0.43	3
Zcchc11	UniProt: A2A8R7	86	0.06	3

a Average Mascot score for the specified protein in two replicate Zfp609-V5 samples.

b Average emPAI score for the specified protein in two replicate Zfp609-V5 samples.

c Average number of unique, non-redundant peptides for the specified protein in the Zfp609-V5 sample.

## Accession Numbers

ChIP- and RNA-seq data have been deposited in the European Nucleotide Archive under ID code ENA: ERP013418.

## Supplemental Information

Document S1. Supplemental Experimental Procedures, Figures S1–S5, and Tables S1 and S3–S5

Table S2. Differentially Expressed Genes Identified by RNA-Seq Analysis in Control, *Zfp609*, *Nipbl*, *Ints1*, or *Ints11* shRNA-Transduced NSCs Related to Experimental Procedures.

Document S2. Article plus Supplemental Information
