# Supplementary material for: Nipbl Interacts with Zfp609 and the Integrator Complex to Regulate Cortical Neuron Migration
Source: Neuron. 2017 Jan 18;93(2):348–61. doi: 10.1016/j.neuron.2016.11.047 (PMC5263256; doi:10.1016/j.neuron.2016.11.047)
Supplement: Document S1. Supplemental Experimental Procedures, Figures S1–S5, and Tables S1 and S3–S5 [file mmc1.pdf]

Neuron, Volume *93*

Supplemental Information

Nipbl Interacts with Zfp609 and the Integrator Complex to Regulate Cortical Neuron Migration

Debbie L.C. van den Berg, Roberta Azzarelli, Koji Oishi, Ben Martynoga, Noelia Urbán, Dick H.W. Dekkers, Jeroen A. Demmers, and François Guillemot

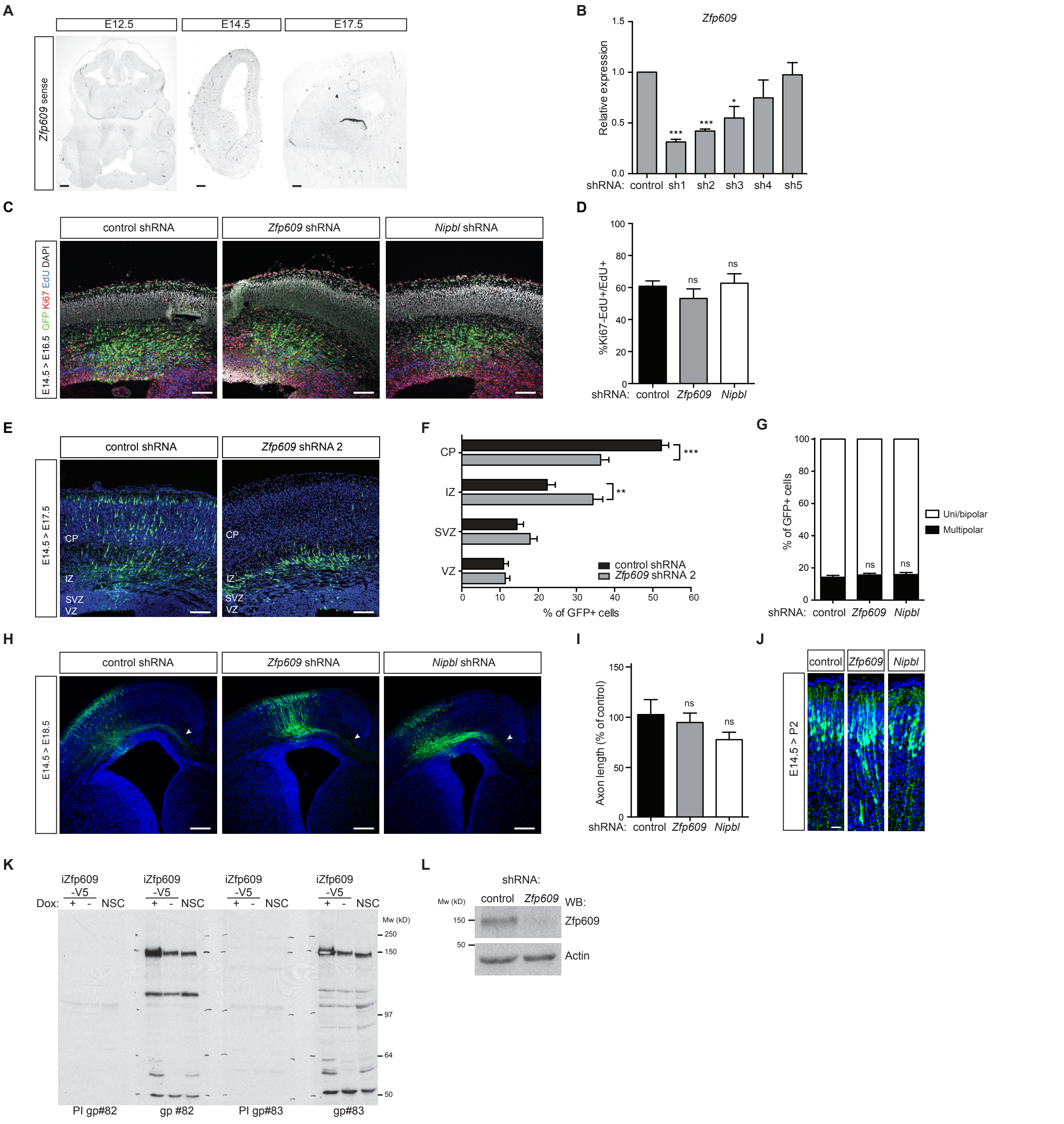

Figure S1, related to Figures 1 and 2.
(A) Composite brightfield images of in situ hybridization with sense Zfp609 probe on mouse embryonic brain sections at indicated developmental stages. (B) Quantitative PCR (qPCR) analysis of transiently transfected P19 cells with *Zfp609* targeting shRNAs. Expression levels were normalized to housekeeping genes (*Tbp* and *Hprt*) and plotted relative to control shRNA transfected cells. * p<0.05, *** p<0.001 unpaired Student's t-test, n=3. (C) Immunohistochemistry on coronal sections of E16.5 mouse embryonic brains electroporated with control, *Zfp609* and *Nipbl* shRNAs and pulse labelled with EdU at E15.5 (D) Quantification of GFP+ cells in (C) showing cell cycle exit rates for different shRNA constructs. Ns, not significant, Student's t-test, n=3 (control) and 4 (*Zfp609* and *Nipbl* shRNA). (E) Immunohistochemistry with GFP antibody on coronal sections of E17.5 mouse embryonic brains electroporated with indicated shRNA constructs. Ventricular (VZ), subventricular (SVZ), intermediate zone (IZ) and cortical plate (CP) are indicated. (F) Quantification of (E) showing percentage of GFP expressing cells in different cortical regions. ** p<0.01, *** p<0.001 unpaired Student's t-test, n=7. (G) Morphological classification of electroporated neurons in cortical plate at E17.5. Ns, not significant, Student's t-test, n=7. (H) Coronal sections of electroporated brains at P0. Arrowheads indicate axons projecting towards the midline. (I) Quantification of axon lengths in primary neuronal cultures of ex vivo electroporated embryonic brains. Ns, not significant, Student's t-test, n=3. (J) Immunohistochemistry with GFP antibody on P2 coronal sections showing apical dendrites in marginal zone. (K) Western blot with pre-immune and anti-Zfp609 sera from two immunized guinea pigs on total cell lysates from control or inducible Zfp609-V5 expressing NS cells. Band at 150 kD represents Zfp609. Doxycycline was added where indicated to induce ectopic expression of Zfp609-V5. (L) Western blot with guinea pig anti-Zfp609 on total cell lysate of control or Zfp609 depleted NS cells demonstrating specificity of the generated antibody. Actin was used as a loading control. Error bars represent SEM. Scale bars represent 200 μm (A,H), 100 μm (C,D) and 20 μm (J).

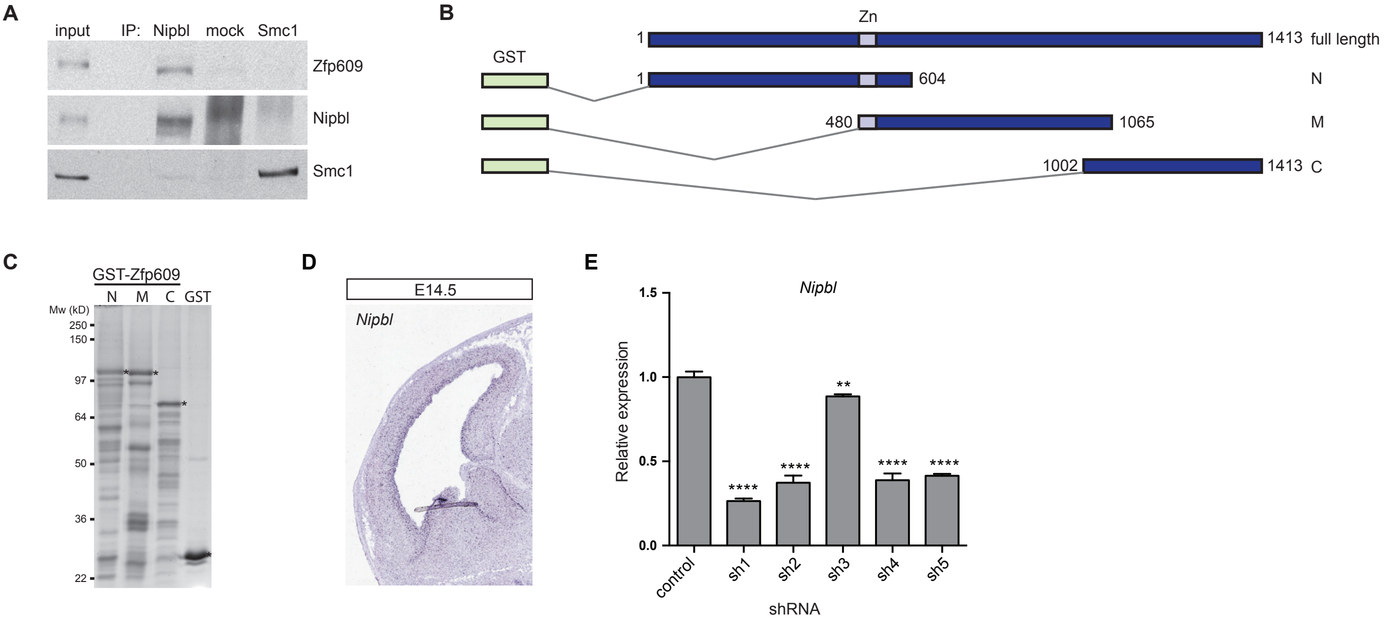

Figure S2, related to Figure 2.

(A) Immunoprecipitates of Nipbl and Smc1 analyzed by Western blotting with the indicated antibodies. Benzonase was added to all samples. Normal rabbit IgG was used as control.

(B) Schematic representation of Zfp609 fragments fused to GST used in GST pull down assays.

(C) Coomassie stained SDS-PAA gel of GST-pull down fractions showing equal loading of GST-fusion proteins, indicated by *.

(D) Genepaint (www.genepaint.org) in situ hybridisation for Nipbl on sagittal section of E14.5 mouse brain showing enriched expression in the ventricular zone.

(E) Quantitative PCR analysis of transiently transfected P19 cells with *Nipbl* targeting shRNAs. Expression levels were normalized to housekeeping genes (*Tbp* and *Hprt*) and plotted relative to control shRNA transfected cells. Error bars represent SEM, ** $p<0.01$, **** $p<0.0001$ unpaired Student's t-test, n=3.

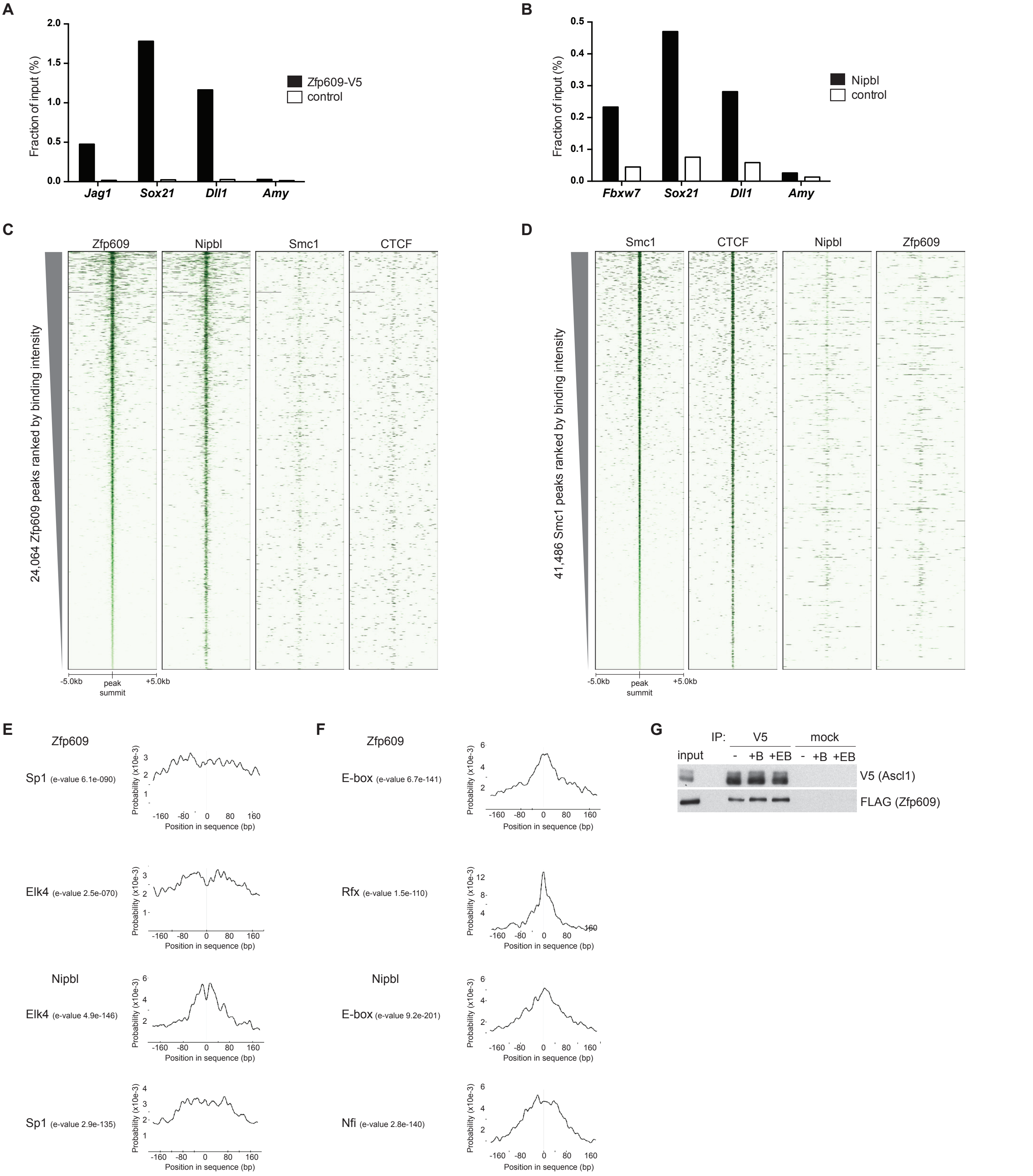

Figure S3, related to Figure 3.

(A) Binding of Zfp609-V5 to *Jag1* (+16 kb), *Sox21* (+6.5 kb) and *Dll1* promoter regions analysed by qPCR of anti-V5 ChIP material. *Amy2a5* promoter region was used as an internal negative control. NS cells not expressing Zfp609-V5 were used as control.

(B) Binding of Nipbl to *Fbxw7* (+125 kb), *Sox21* (+6.5 kb) and *Dll1* promoter regions analysed by qPCR of anti-Nipbl ChIP material. *Amy2a5* promoter region was used as an internal negative control region and rabbit IgG was used as control.

(C) Heatmap of 24,064 Zfp609 bound regions displaying control-normalized mean read counts for indicated factors in a 10 kb window centered around Zfp609 peak summits.

(D) Heatmap of 41,486 Smc1 bound regions displaying control-normalized mean read counts for indicated factors in a 10 kb window centered around Smc1 peak summits

(E) Centrimo enrichment profiles for significantly enriched motifs around proximal Zfp609 (top) and Nipbl (bottom) peak summits.

(F) Centrimo enrichment profiles for significantly enriched motifs around distal Zfp609 (top) and Nipbl (bottom) peak summits.

(G) Western blot analysis with indicated antibodies of V5 immunopreciptates from NS cells ectopically expressing V5-Ascl1 and Zfp609-FLAG. Mouse IgG was used as control. Benzonase (+B) and ethidium bromide (+EB) were added as indicated.

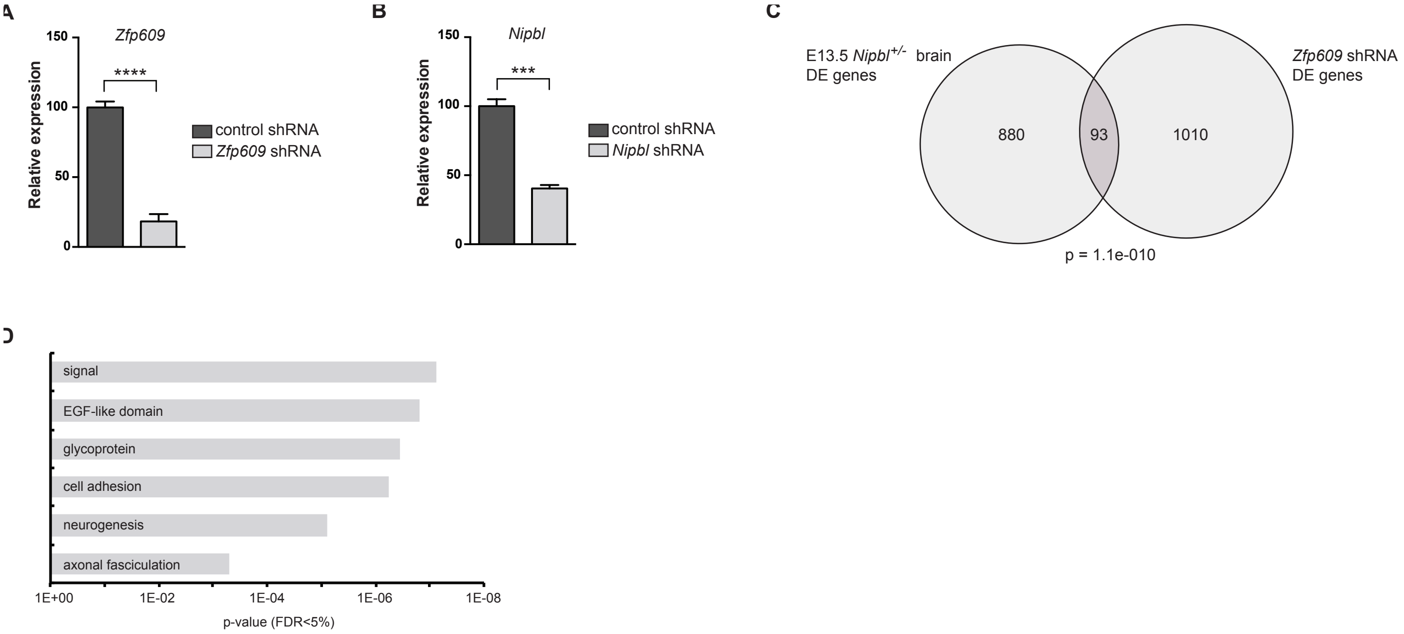

Figure S4, related to Figure 4.

(A) qPCR analysis of NS cells transduced with *Zfp609* or control shRNA expressing lentivirus. *Zfp609* expression levels were normalized to housekeeping genes (*Tbp* and *Hprt*) and plotted relative to control shRNA transduced cells. Error bars represent SEM, ****p<0.0001 unpaired Student's t-test, n=3.

(B) qPCR analysis of NS cells transduced with *Nipbl* or control shRNA expressing lentivirus. *Nipbl* expression levels were normalized to housekeeping genes (*Tbp* and *Hprt*) and plotted relative to control shRNA transduced cells. Error bars represent SEM, ***p<0.001 unpaired Student's t-test, n=3.

(C) Venn diagram showing overlap of differentially expressed (DE) genes between E13.5 *Nipbl*$^{+/-}$ brain and Zfp609 depleted NS cells. P-value from hypergeometric test is shown.

(D) Gene ontology analysis on common DE genes from (C). DAVID p-values are shown, FDR<5%.

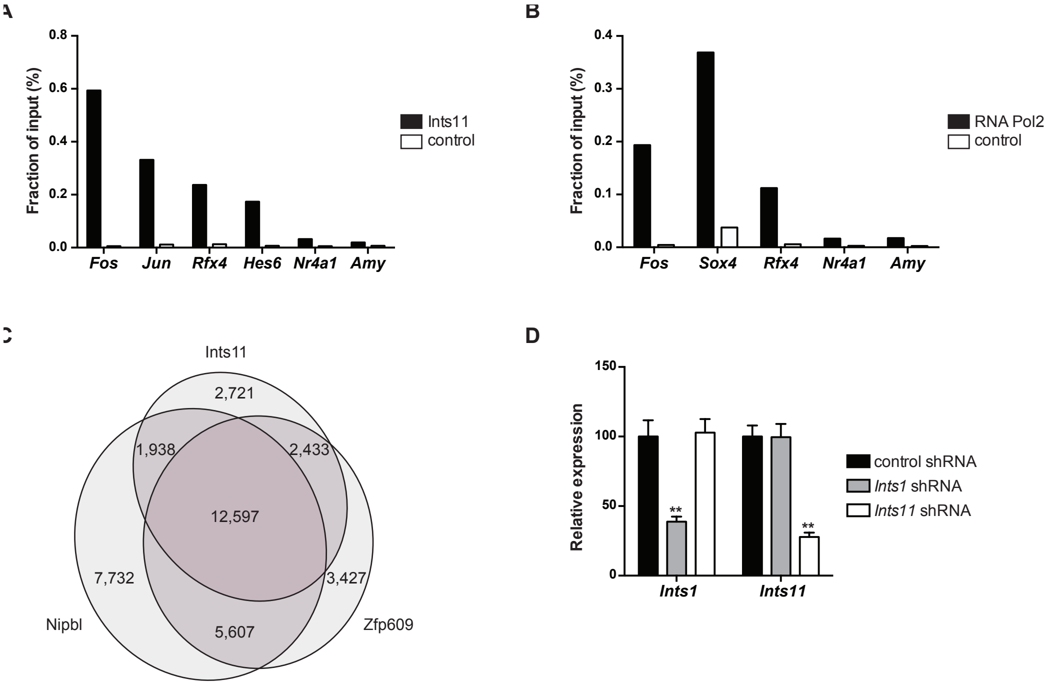

Figure S5, related to Figure 5.
(A) Binding of Ints11 to *Fos*, *Jun*, *Rfx4*, *Hes6* and *Nr4a1* promoter regions analysed by qPCR of Ints11 ChIP. *Amy2a5* promoter was used as internal negative control region. GFP ChIP was used as control.
(B) Binding of RNA pol2 to *Fos*, *Sox4*, *Rfx4* and *Nr4a1* promoter regions analysed by qPCR of Ints11 ChIP. *Amy2a5* promoter was used as internal negative control region. GFP ChIP was used as control.
(C) Venn diagram demonstrating overlap of Ints11, Zfp609 and Nipbl bound regions.
(D) qPCR analysis of NS cells transduced with lentivirus expressing control, *Ints1* or *Ints11* targeting shRNAs. Expression levels were normalized to housekeeping genes (Tbp and Hprt) and plotted relative to control shRNA transduced cells. Error bars represent SEM, **p<0.01 Unpaired Student's t-test corrected for multiple comparisons using the Holm-Sidak method, n=3.

Table S1, related to Table 1.
Zfp609-V5 interacting proteins as identified by mass spectrometry in replicate samples of Zfp609-V5 and control purifications.

		Zfp609-V5 #1			Zfp609-V5 #2			control #1			control #2		
**Protein name**	**Accession**	**Mascot**	**emPAI**	**Pept**	**Mascot**	**emPAI**	**Pept**	**Mascot**	**emPAI**	**pept**	**Mascot**	**emPAI**	**pept**
Zfp609	Q8BZ47	4297	11.6	68	4330	26.98	74	388	0.3	9	735	0.53	17
**Cohesin complex**													
Nipbl	Q6KCD5	1577	0.44	33	4332	2.7	93	nd	nd	nd	1082	0.37	28
Smc3	Q9CW03	881	0.55	18	3275	6.76	63	nd	nd	nd	911	0.73	22
Smc1a	Q9CU62	791	0.54	18	3304	7.08	70	nd	nd	nd	1016	0.88	26
Stag2	A2AFF6	228	0.13	5	1291	1.16	27	nd	nd	nd	137	0.09	4
Rad21	Q61550	287	0.35	6	1177	2.96	27	nd	nd	nd	73	0.09	2
Mau2	Q9D2X5	185	0.23	4	626	1.48	14	nd	nd	nd	161	0.26	5
**Integrator complex**													
Ints1	K3W4P2	2259	1.1	47	3560	3.46	78	nd	nd	nd	813	0.59	20
Ints6	Q6PCM2	1592	2.08	29	2076	5.89	43	nd	nd	nd	406	0.57	12
Ints3	Q7TPD0	1494	1.81	27	1933	3.07	37	nd	nd	nd	566	0.5	13
Ints7	Q7TQK1	1365	1.24	23	1679	3.32	31	nd	nd	nd	192	0.2	5
Asun	Q8QZV7	1282	1.82	24	1620	9.79	33	nd	nd	nd	344	0.41	8
Ints5	Q8CHT3	1015	1.01	19	970	1.42	19	nd	nd	nd	316	0.3	7
Ints2	Q80UK8	650	0.34	11	1333	1.31	27	nd	nd	nd	312	0.21	8
Cpsf3l	Q9CWS4	766	1.4	16	971	3.27	21	nd	nd	nd	247	0.32	6
Vwa9	Q8R3P6	521	0.99	11	902	2.2	17	nd	nd	nd	145	0.25	4
Ints8	Q80V86	541	0.47	12	950	1.32	21	nd	nd	nd	199	0.18	6
Ints9	Q8K114	398	0.54	10	760	2.18	19	nd	nd	nd	122	0.14	3
Ints12	Q9D168	410	0.67	7	739	2.68	15	nd	nd	nd	218	0.48	5
**Transcription factors**													
Rfx4	Q7TNK1	584	0.68	13	935	1.92	23	nd	nd	nd	279	0.36	8
Zbtb20	Q8K0L9	762	0.94	13	481	0.6	8	nd	nd	nd	108	0.12	3
**Other**													
Maged1	Q9QYH6	672	0.73	12	975	1.46	20	nd	nd	nd	281	0.3	7
Hspa2	P17156	684	1.06	10	858	1.74	17	nd	nd	nd	nd	nd	nd
Dnaja2	Q9QYJ0	313	0.72	7	311	0.73	7	nd	nd	nd	92	0.23	3
Stub1	Q9WUD1	223	0.66	6	350	1.94	9	nd	nd	nd	nd	nd	nd
Akap8l	Q9R0L7	166	0.16	3	303	0.43	7	nd	nd	nd	nd	nd	nd
Bag5	Q8CI32	253	0.63	6	157	0.36	5	nd	nd	nd	nd	nd	nd
Cnp	P16330	63	0.08	1	265	0.6	7	nd	nd	nd	nd	nd	nd
Setx	A2AKX3	51	0.01	1	189	0.06	5	nd	nd	nd	nd	nd	nd
Mlf2	Q99KX1	103	0.29	2	113	0.57	3	nd	nd	nd	nd	nd	nd
Zcchc11	A2A8R7	54	0.04	2	117	0.07	4	nd	nd	nd	nd	nd	nd
Nabp2	E9Q199	53	0.17	1	96	0.32	2	nd	nd	nd	nd	nd	nd

Mascot score, emPAI score and number of unique, non-redundant peptides for indicated samples as in Table 1. Nd, not detected.

Table S2, related to Experimental Procedures.
Differentially expressed genes identified by RNA-Seq analysis in control, *Zfp609*, *Nipbl*, *Ints1* or *Ints11* shRNA transduced NS cells.

SUPPLEMENTAL EXPERIMENTAL PROCEDURES

Antibody Research Resource Identifiers

Normal mouse IgG Santa Cruz Biotechnology Cat# sc-2025 RRID:AB_737182; normal rabbit IgG Santa Cruz Biotechnology Cat# sc-2027 RRID:AB_737197; Lamin B1 Santa Cruz Biotechnology Cat# sc-6216 RRID:AB_648156 and Santa Cruz Biotechnology Cat# sc-6217 RRID:AB_648158; RNA pol2 Santa Cruz Biotechnology Cat# sc-899 RRID:AB_632359; Smc1 Bethyl Cat# A300-055A RRID:AB_2192467; Nipbl Bethyl Cat# A301-779A RRID:AB_1211232; Int1 Bethyl Cat# A300-361A RRID:AB_2127258; Int11 Bethyl Cat# A301-274A RRID:AB_937779; V5 Thermo Fisher Scientific Cat# R960-25 RRID:AB_2556564; Actin Sigma-Aldrich Cat# A2066 RRID:AB_476693; Vcp Abcam Cat# ab11433 RRID:AB_298039; GFP AbD Serotec Cat# 4745-1051 RRID:AB_619712; GFP Santa Cruz Biotechnology Cat# sc-8334 RRID:AB_641123; GFP Abcam Cat# ab13970 RRID:AB_300798; Ki67 BD Biosciences Cat# 550609 RRID:AB_393778.

Nuclear extract preparation

Control and Zfp609-V5 expressing NS cells were grown to near confluency in 14 cm diameter dishes, scraped in 2 ml ice cold PBS supplemented with 1x complete EDTA-free protease inhibitors (Roche) and spun down at 200g at 4°C. Cell pellet volumes (PV) determined at this point were used as a reference throughout the procedure. Cells were resuspended in 5 PV buffer A (10 mM Hepes pH7.6, 1.5 mM $MgCl_2$, 10 mM KCl), incubated for 10 min on ice, harvested by centrifugation at 1400g and lysed in 2 PV buffer A using 10 strokes with pestle A in a dounce homogenizer. Nuclei were harvested by centrifugation at 1400g and proteins were extracted in 1.5 PV buffer C (20mM Hepes pH7.6, 0.2 mM EDTA, 1.5 mM $MgCl_2$, 420 mM NaCl, 20% glycerol) with 10 strokes of pestle B in a dounce homogenizer. Extracts were incubated for 30 min at 4°C and debris was removed by high speed centrifugation. All buffers were precooled on ice and supplemented with 1x complete EDTA-free protease inhibitors (Roche). Nuclear extracts were diluted two-fold to approximately 100 mM NaCl with buffer C-0 (20 mM Hepes pH7.6, 0.2 mM EDTA, 1.5 mM $MgCl_2$, 20% glycerol).

Mass spectrometric analysis

Gel lanes were cut into 2 mm slices using an automatic gel slicer and subjected to in-gel reduction with dithiothreitol, alkylation with iodoacetamide and digestion with trypsin (Promega, sequencing grade), essentially as described by (Wilm et al., 1996). Nanoflow LCMS/MS was performed on an 1100 series capillary LC system (Agilent Technologies) coupled to an LTQ-Orbitrap mass spectrometer (Thermo) operating in positive mode and equipped with a nanospray source. Peptide mixtures were trapped on a ReproSil C18 reversed phase column (Dr Maisch GmbH; column dimensions 1.5 cm × 100 µm, packed in-house) at a flow rate of 8 µl/min. Peptide separation was performed on ReproSil C18 reversed phase column (Dr Maisch GmbH; column dimensions 15 cm × 50 µm, packed in-house) using a linear gradient from 0 to 80% B (A = 0.1 % formic acid; B = 80% (v/v) acetonitrile, 0.1 % formic acid) in 70 min and at a constant flow rate of 200 nl/min using a splitter. The column eluent was directly sprayed into the ESI source of the mass spectrometer. Mass spectra were acquired in continuum mode; fragmentation of the peptides was performed in data-dependent mode. Peak lists were automatically created from raw data files using the Mascot Distiller software (version 2.1; MatrixScience). The Mascot search algorithm (version 2.2, MatrixScience) was used for searching against the NCBInr database (release NCBInr_20090222; taxonomy: Mus musculus) or the IPI_mouse_database (release 20090924). The peptide tolerance was typically set to 10ppm and the fragment ion tolerance to 0.8 Da. A maximum number of 2 missed cleavages by trypsin were allowed and carbamidomethylated cysteine and oxidized methionine were set as fixed and variable modifications, respectively. The Mascot score cut-off value for a positive protein hit was set to 60, based on at least two peptides. In case of protein identifications with Mascot scores between 50 and 60, or that were based on only one peptide, individual peptide MS/MS spectra were checked manually and either interpreted as valid identifications or discarded. We also show a more quantitative measure of our identified proteins, emPAI score (Ishihama et al., 2005). emPAI score incorporates the number of peptides identified per protein (spectral counts) normalized by the theoretical number of peptides. This is a superior method over just counting the number of identified peptides, because it takes account of the fact that, for the same number of molecules, larger proteins and proteins with many peptides in the preferred mass range for mass spectrometry will generate more observed peptides.

GST pull down

BL21-CodonPlus (DE3)-RP competent cells containing GST expression constructs were grown in the presence of 50 µM $ZnCl_2$ to an OD_{600} = 0.3-0.6. Expression of GST-fusion proteins and GST was induced for 3 hours at 25°C by the addition of 0.4 mM IPTG. Cells were lysed in lysis buffer (25 mM Hepes pH7.6, 5 mM $MgCl_2$, 150 mM NaCl, 10% glycerol, 0.1% NP-40, 50 µM $ZnCl_2$), sonicated and lysates were incubated with glutathione sepharose beads (GE Healthcare) for 2 hours at 4°C. Beads were equilibrated in buffer C-100* and incubated with 200 µl NS cell nuclear extract in the presence of 25 units Benzonase. Bound proteins were analysed by Western blotting.

Chromatin immunoprecipitation

For dual crosslinking NS cells were scraped and washed several times in RT PBS, crosslinked for 45 min at RT with 2 mM disuccinimidyl glutarate (DSG), washed four times with RT PBS and crosslinked for 10 min at RT by the addition of 1/10 volume of 11% buffered formaldehyde solution (50 mM Hepes pH7.6, 100 mM NaCl, 1 mM EDTA, 0.5 mM EGTA, 11% formaldehyde). For RNA polymerase 2 ChIP 1/10 volume of 11% buffered formaldehyde was added directly to the medium. Reactions were quenched for 10 min at RT by the addition of glycine to a final concentration of 125 mM and cells were washed twice with ice cold PBS before being flash frozen in liquid nitrogen. Cell pellets were thawed and incubated for 10 min at 4°C in 5 ml LB1 (50 mM Hepes pH7.6, 140 mM NaCl, 1 mM EDTA, 10% glycerol, 0.5% NP-40, 0.25% Triton X-100) per $1x10^8$ cells. Cell nuclei were collected by centrifugation at 1400g for 5 min at 4°C. Nuclei were incubated for 10 min at 4°C in 5 ml LB2 (10 mM Tris pH8.0, 200 mM NaCl, 1 mM EDTA, 0.5 mM EGTA) per $1x10^8$ cells and collected by centrifugation at 1400g for 5 min at 4°C. Nuclei from $1x10^8$ cells

were then resuspended in 3 ml LB3 (10 mM Tris pH8.0, 100 mM NaCl, 1 mM EDTA, 0.5 mM EGTA, 0.1% Na-deoxycholate, 0.5% N-lauroylsarcosine) and chromatin sonicated to 200 bp fragments in a cooled water bath Bioruptor sonicator (Diagenode). Triton X-100 was added to a final concentration of 1%. For RNA pol2 ChIP cells were sonicated in Pol2 sonication buffer (50 mM Tris-HCl pH 7.5, 140 mM NaCl, 1 mM EDTA, 1 mM EGTA, 1% Triton X-100, 0.1% Na-deoxycholate, 0.1% SDS). 1.5 ml chromatin was precleared with 60 µl protein A or protein G dynabeads equilibrated in LB3 or RNA pol2 sonication buffer and blocked with 0.5 mg/ml BSA. Chromatin was incubated o/n at 4°C with 10 µg antibody and antibody-chromatin complexes were bound to 100 µl equilibrated and BSA-blocked protein A or protein G dynabeads for 1 hour at 4°C. Beads were washed 5 times with RIPA buffer (50 mM Hepes pH7.6, 500 mM LiCl, 1 mM EDTA, 1% NP-40, 0.7% Na-deoxycholate) and once with TE containing 50 mM NaCl. RNA pol2 ChIP was washed 3 times in Pol2 sonication buffer, once in Pol2 sonication buffer containing 500 mM NaCl, once in LiCl wash buffer (20 mM Tris pH 8.0, 1 mM EDTA, 250 mM LiCl, 0.5% NP-40, 0.5% Na-deoxycholate) and once in TE. All lysis and wash buffers were precooled on ice and supplemented with 1x complete EDTA-free protease inhibitors. Chromatin was eluted with 200 µl elution buffer (50 mM Tris pH8.0, 10 mM EDTA, 1% SDS) in a shaking heatblock at 65°C. Crosslinks of 1% input and eluted chromatin were reversed by incubation at 65°C o/n. The elution buffer was diluted two-fold with $T_{50}E_{10}$ and incubated with 0.2 µg/ml RNaseA for 1 hour at 37°C and 0.2 µg/ml proteinase K for 2 hours at 45°C. Samples were extracted twice with phenol:chloroform:isoamyl-alcohol and ethanol precipitated for 30 min at -80°C in the presence of 200 mM NaCl and 30 µg glycogen. DNA pellets were dissolved in 20 µl H_2O, DNA content was quantified by Qubit (Thermo Fisher Scientific) and 2-10 ng was used to prepare sequencing libraries as described in the main text.

Statistical analysis
Statistical tests described in the Figure Legends were performed using Prism software.

Proliferation and axonogenesis
For determination of cell cycle exit rates E14.5 electroporated pregnant mice were injected with 30 µg per gram of bodyweight EdU at E15.5. Embryos were collected at E16.5 and processed as described in the main text. Coronal cryosections were treated with 10 mM Na-citrate pH6.0 for 10 minutes at 90°C and stained with antibodies against GFP (Abcam, ab13970) and Ki67 (BD Pharmingen, #550609). EdU was detected with a Click-iT assay kit (Thermo Fisher Scientific). At least 130 cells were counted per embryo and 3-4 embryos per condition were analysed. To measure the length of axonal projections E14.5 embryonic heads were used for ex vivo electroporation with five 50V pulses at 1s intervals. Brains were dissected and electroporated cortical regions dissociated in L-15 medium supplemented with 10mM Hepes. Cells were seeded on poly-D-lysine and laminin precoated coverslips in Neurobasal medium supplemented with N2, B27, glutamine and Penicillin-Streptomycin. After 2 days in vitro cells were fixed with 4% paraformaldehyde, treated with 10mM Na-citrate pH6.0 at 90°C and stained with GFP and Smi-312 (Biolegend, #837904) antibodies. Axonal lengths of at least 150 cells per experiment were measured using Image J.

Table S3, related to Experimental Procedures.
Targeting sequences of shRNAs used in this study.

Zfp609 shRNA1	GGGAGGAACTGGAACATAA
Zfp609 shRNA2	GCTACAGTGACCAGAGTTA
Nipbl shRNA1	GGAGGGTTATTAAGTTCAA
Nipbl shRNA2	CGCTTCTCAAAGGAAGTTCAA
Ints1 shRNA	GTGCTCCTCAACCACTATA
Ints11 shRNA	GCAGCCATGTTCCAGATTAAA
control shRNA	GCGCGCTTTGTAGGATTCG

Table S4, related to Experimental Procedures.
Primer sequences used for qPCR analysis.

**ChIP**	**Forward**	**Reverse**
Jag1 (+16 kb)	AAGGACAACTCCCTGGAGAA	CTGTTAGTGCCCTGTCTGGA
Sox21 (+6.5 kb)	TTATTTATGCGCCTGCTCCT	GGAATGAATAGCGGTGCAAT
Dll1 (TSS)	GCGTGGCTGTCATTAAGG	GGTGCTGTCTGCATTACC
Fbxw7 (+125 kb)	CAGCTATGTTCCTGCTGTGC	CAACTTCTGCCTGCTTCCTC
Rfx4 (TSS)	TAATGAGTGCCGGGCTAAGG	CCATTGTGGGCCTGTTTTGA
Fos (TSS)	AGCGAGCAACTGAGAAGACT	TCATGGTCGAAGTTTGGGGA
Hes6 (TSS)	GCTCCAGCGTTCTTGGATTT	GCTCCTTGTCCTGGCTCTAA
Sox4 (TSS)	TGCAAGGTAGGAAGCCAAGA	GGTAACCAGCTCCCCTTCTT
Nr4a1 (TSS)	TAGTGGGCGCTTGTTTAGGA	AACCCAGAGTACAGAGTGCC

Amy2a5 (TSS)	CTCCTTGTACGGGTTGGT	AATGATGTGCACAGCTGAA
**cDNA**		
Zfp609	CCTGCTGTGATGATGCAGA	GCCATACGGGGAGAAAGAAT
Nipbl	TGAATCTACCATGCCACTTTGT	CTTCTTCTGTCGCGCTCTTC
Sema3a	TTGCCTGTCTTTTCTGGGGT	AGTCTACTCCGTTCTTCATCCA
Nrp1	ACCTCACATCTCCCGGTTAC	AGAGAAAGGGCCCTGAAGAC
Gabbr2	TCACTCTCTGCCTGGTGTTT	GTAGGCGGTGGTTTTCTGAC
Ints1	GAGAAGCGAGCCATTTCTCC	CTTCAATCTCGTCCAGCAGC
Ints11	AGTGAAATGGTGGGCTACGA	CACGTGGCCTGCATAGTATG
Hprt	AGCCTAAGATGAGCGCAAGT	ATGGCCACAGGACTAGAACA
Tbp	GGGGAGCTGTGATGTGAAGT	CCAGGAAATAATTCTGGCTCA

The following Taqman probes from Applied Biosystems were used: *Plxnd1* Mm01184367_m1, *Actb* 4352933E.

Table S5, related to Experimental Procedures.
Primer sequences used for in situ probe template.

	**Forward**	**Reverse**
Zfp609	CCTCCTTATGGCTACAGTGACC	TTCCTTGGGAACAGAGTCCTTA

SUPPLEMENTAL REFERENCE

Wilm, M., Shevchenko, A., Houthaeve, T., Breit, S., Schweigerer, L., Fotsis, T., and Mann, M. (1996). Femtomole sequencing of proteins from polyacrylamide gels by nano-electrospray mass spectrometry. Nature *379*, 466-469.
