# Supplementary material for: Nipbl Interacts with Zfp609 and the Integrator Complex to Regulate Cortical Neuron Migration
Source: Neuron. 2017 Jan 18;93(2):348–61. doi: 10.1016/j.neuron.2016.11.047 (PMC5263256; doi:10.1016/j.neuron.2016.11.047)
Supplement: Document S2. Article plus Supplemental Information [file mmc3.pdf]

Neuron

Article

Nipbl Interacts with Zfp609 and the Integrator Complex to Regulate Cortical Neuron Migration

Highlights

- Nipbl interacts with the transcription factor Zfp609 and the Integrator complex
- Nipbl, Zfp609, and Integrator are required for cortical neuron migration
- Nipbl, Zfp609, and Integrator co-occupy genomic binding sites independently of cohesin
- Nipbl, Zfp609, and Integrator directly regulate neuronal migration genes

Authors

Debbie L.C. van den Berg, Roberta Azzarelli, Koji Oishi, ..., Dick H.W. Dekkers, Jeroen A. Demmers, François Guillemot

Correspondence

debbie.vandenberg@crick.ac.uk (D.L.C.v.d.B.), francois.guillemot@crick.ac.uk (F.G.)

In Brief

NIPBL mutations cause Cornelia de Lange syndrome, but Nipbl function in brain development is not well understood. Van den Berg et al. show that Nipbl interacts with Zfp609 and the Integrator complex to transcriptionally regulate cortical neuron migration.

van den Berg et al., 2017, Neuron *93*, 348–361
January 18, 2017 © 2017 The Authors. Published by Elsevier Inc.
http://dx.doi.org/10.1016/j.neuron.2016.11.047

CellPress

Nipbl Interacts with Zfp609 and the Integrator Complex to Regulate Cortical Neuron Migration

Debbie L.C. van den Berg,[1,*] Roberta Azzarelli,[1] Koji Oishi,[1] Ben Martynoga,[1] Noelia Urbán,[1] Dick H.W. Dekkers,[2] Jeroen A. Demmers,[2] and François Guillemot[1,3,*]
[1]The Francis Crick Institute, Mill Hill Laboratory, The Ridgeway, London NW7 1AA, UK
[2]Center for Proteomics, Erasmus MC, Wytemaweg 80, 3015 CN Rotterdam, the Netherlands
[3]Lead Contact
*Correspondence: debbie.vandenberg@crick.ac.uk (D.L.C.v.d.B.), francois.guillemot@crick.ac.uk (F.G.)
http://dx.doi.org/10.1016/j.neuron.2016.11.047

SUMMARY

Mutations in *NIPBL* are the most frequent cause of Cornelia de Lange syndrome (CdLS), a developmental disorder encompassing several neurological defects, including intellectual disability and seizures. How *NIPBL* mutations affect brain development is not understood. Here we identify Nipbl as a functional interaction partner of the neural transcription factor Zfp609 in brain development. Depletion of Zfp609 or Nipbl from cortical neural progenitors in vivo is detrimental to neuronal migration. Zfp609 and Nipbl overlap at genomic binding sites independently of cohesin and regulate genes that control cortical neuron migration. We find that Zfp609 and Nipbl interact with the Integrator complex, which functions in RNA polymerase 2 pause release. Indeed, Zfp609 and Nipbl co-localize at gene promoters containing paused RNA polymerase 2, and Integrator similarly regulates neuronal migration. Our data provide a rationale and mechanistic insights for the role of Nipbl in the neurological defects associated with CdLS.

INTRODUCTION

The cerebral cortex, responsible for higher cognitive function, is generated from a pool of progenitor cells that will give rise to the neuronal and glial lineages of the adult brain. Unperturbed migration of newly born neurons across the expanding cortex to their final destination in specific cortical layers ensures accurate connectivity and neuronal circuit formation. Cell-intrinsic transcription factors play key roles in orchestrating the underlying molecular processes, as was recently shown for the proneural transcription factors Neurog2 and Ascl1 (Heng et al., 2008; Pacary et al., 2011). Developmental disturbance of neuronal migration affects shaping of the neuronal network and has been linked to a variety of neurological disorders, including epilepsy, schizophrenia, autism spectrum disorder (ASD), and intellectual disability (Guerrini and Parrini, 2010; Muraki and Tanigaki, 2015; Reiner et al., 2016; Verrotti et al., 2010).

Cornelia de Lange syndrome (CdLS) is one particular example of a developmental disorder highlighted by neurological defects, including seizures and intellectual disability. Other characteristics include facial dysmorphism, growth retardation, and upper limb defects. Heterozygous mutations in the cohesin loading factor Nipped-B-like (*NIPBL*) have been identified in 50%–60% of cases and are associated with a more severe clinical presentation, while mutations in cohesin complex subunits *SMC1A*, *SMC3*, and *RAD21* and in the SMC3-targeting deacetylase *HDAC8* account for a further 10% of mostly mildly affected cases (Braunholz et al., 2015). A genetic cause for the remaining 30% of clinically diagnosed CdLS patients remains unknown.

Despite cohesin complex subunits originally having been identified for their role in sister chromatid cohesion (Michaelis et al., 1997), studies have failed to detect overt chromosome segregation defects in CdLS patients, and instead, deregulated gene expression is thought to be the prime cause of the observed developmental abnormalities (Castronovo et al., 2009; Deardorff et al., 2012; Kawauchi et al., 2009; Liu et al., 2009; Remeseiro et al., 2013). This likely relates to the ability of cohesin to mediate long-range chromosome interactions in *cis*, thereby facilitating enhancer-promoter looping (Kagey et al., 2010; Nativio et al., 2009; Seitan et al., 2013).

How Nipbl acts in gene regulatory networks and developmental pathways in brain development is poorly understood. In this study, we set out to identify new regulators of cortical development by studying a mouse ortholog of *Drosophila scribbler (sbb)*, the single zinc-finger protein Zfp609. We identified Nipbl as a binding partner of Zfp609, which is specifically expressed in neural progenitors in the developing mouse cortex. Zfp609 and Nipbl interact and co-bind genomic regions with the RNA polymerase 2 (RNA pol2)-associated Integrator complex to directly regulate neuronal migration genes. Accordingly, depletion of Zfp609, Nipbl, or Integrator from cortical progenitors in vivo results in neuronal migration defects. Our findings define a Nipbl transcriptional pathway relevant to CdLS.

RESULTS

Zfp609 Is Expressed in Neural Progenitors and Regulates Cortical Neuron Migration

Zfp608 and Zfp609 are vertebrate homologs of *Drosophila* scribbler, a single zinc-finger protein that is highly expressed in the larval CNS, where it is proposed to act as a transcription factor

This is an open access article under the CC BY license (http://creativecommons.org/licenses/by/4.0/).

(Haecker et al., 2007; Yang et al., 2000). *Zfp608* is specifically expressed in the mouse forebrain subventricular (SVZ) and intermediate zone (IZ) at embryonic day (E)14.5 (Ayoub et al., 2011). To delineate the expression domain of *Zfp609*, we performed in situ hybridization on brain sections at different developmental stages (Figures 1A and S1A, available online). *Zfp609* transcripts are enriched in and subsequently become restricted to the progenitor population as cortical neurogenesis peaks at E14.5. The absence of *Zfp609* transcripts from cells in the SVZ/IZ at this stage could be attributed to direct repression by Zfp608, as occurs in developing thymocytes (Reed et al., 2013). At later stages of development, *Zfp609* expression is detected in the neurons of the cortical plate (CP) and in stem cells near the ventricular surface.

Because *scribbler* mutations affect axon targeting and larval locomotion (Rao et al., 2000; Suster et al., 2004; Yang et al., 2000), we decided to assess the role of its vertebrate homologs in brain development. Based on their expression pattern and the assumption that any disruption to the progenitor population would affect downstream lineages, we decided to focus our initial analysis on Zfp609. To address the importance of *Zfp609* expression in mouse neural progenitor cells (NPCs) in vivo, we electroporated short hairpin RNA (shRNA) constructs into E14.5 mouse embryonic brains (Tabata and Nakajima, 2001). We designed two independent shRNAs that efficiently deplete Zfp609 at the transcript and protein level (Figures 1B and S1B). Each shRNA construct was injected along with a GFP expression vector into the lateral ventricles of E14.5 mouse embryos and transduced into NPCs near the ventricular surface by a series of electric pulses. We first analyzed the effect of Zfp609 depletion on progenitor proliferation by labeling dividing cells by EdU incorporation at E15.5. The fraction of transduced cells that had exited the cell cycle 24 hr later, identified as labeled by EdU and negative for Ki67, did not significantly differ between the two populations (Figures S1C and S1D).

At E14.5, NPCs give rise to upper-layer cortical neurons and, consistent with this, the majority of control shRNA transduced neurons were found in superficial positions in the CP at E17.5 (Figures 1C and 1D). In contrast, the neuronal progeny of Zfp609-depleted NPCs had an abnormal multipolar morphology and accumulated in the IZ, a phenotype that was confirmed by an independent *Zfp609*-targeting shRNA (Figures 1C–1F, S1E, and S1F). Zfp609-deficient neurons that reached the CP acquired a bipolar morphology, and neither apical dendrites nor axonal length or projection toward the midline were affected (Figures S1G–S1J). Strikingly, heterotopic cell clusters were observed in the white matter of postnatal Zfp609 knockdown (KD) mice (Zfp609 KD, 2/3 mice; control, 0/3 mice; Figure 1G). To further rule out that the aberrant neuronal positioning is due to off-target effects of the shRNAs, we generated an shRNA-resistant Zfp609 construct harboring three silent mutations, designated Zfp609* (Figure 1B). Co-electroporation of Zfp609* fully rescued the IZ accumulation observed for Zfp609-depleted cells (Figures 1C and 1D). Taken together, these data suggest that Zfp609 plays a crucial role in the regulation of cortical neuron migration, which cannot be compensated for by Zfp608.

Neural progenitors can be adherently cultured in vitro and form a valuable model system to study molecular mechanisms of neural stem cell (NSC) identity and differentiation, as their limitless expansion enables generation of sufficient material for proteomics studies and other genome-wide approaches. To explore whether they could be used as a tool to study Zfp609 function, we analyzed Zfp609 transcript and protein levels in embryonic stem cell (ESC)-derived NSCs (Conti et al., 2005). Consistent with the in vivo expression pattern, RNA sequencing (RNA-seq) data showed preferential expression of *Zfp609* over *Zfp608* (Figure 1H). We generated an antibody that specifically recognizes Zfp609 (Figures S1K and S1L) and could detect Zfp609 protein in NSC lysates (Figure 1H). Immunocytochemistry on NSCs expressing V5-tagged Zfp609 showed an exclusively nuclear localization, fitting with its proposed role as a transcription factor (Figure 1I).

Nipbl Is an Interaction Partner of Zfp609 and Regulates Neuronal Migration

To gain insight into the molecular environment of Zfp609, we purified Zfp609 from NSCs and identified its interaction partners by mass spectrometry. Nuclear extracts from NSCs expressing doxycycline-inducible V5-tagged Zfp609 were subjected to V5 affinity purification, and Zfp609-containing protein complexes were separated by SDS-PAA gel electrophoresis (Figure 2A). NSCs not expressing Zfp609-V5 were used as a control and benzonase nuclease was added to eliminate DNA-mediated interactions. Colloidal Coomassie staining of Zfp609-V5 immunoprecipitates showed a prominent band at around 150 kD that reacts with V5 antibody (Figure 2B) and many additional bands not detected in the control purification, probably representing Zfp609-interacting proteins.

Gel lanes of Zfp609-V5 and control purifications were analyzed by mass spectrometry, and interaction partners present in two Zfp609 purifications are listed in Table 1. Mascot scores, emPAI (exponentially modified protein abundance index) scores, a semiquantitative measure (Ishihama et al., 2005), and numbers of identified unique peptides of the replicate samples are shown in Table S1. Interestingly, the cohesin complex, comprised of Smc1a, Smc3, Rad21, and Stag2, and its loading factor Nipbl/Mau2 were highly enriched in Zfp609-V5 fractions. Western blot analysis on Zfp609-V5 immunoprecipitates indeed confirmed co-purification of Smc1 and Nipbl (Figure 2C). Neither benzonase nor ethidium bromide affected this interaction, suggesting it occurs independently of DNA. Furthermore, by antibody immunoprecipitation we could show the interaction of endogenous Nipbl and Zfp609 (Figure 2D). Very little Smc1 was detected in these Nipbl immunoprecipitations, indicating that soluble cohesin at endogenous levels is a substoichiometric interactor of Nipbl and Zfp609 (Figure S2A). Finally, to identify the protein domains involved in a direct interaction between Zfp609 and Nipbl, we expressed GST-fusion proteins representing partially overlapping domains of Zfp609 in bacteria (Figures S2B and S2C). GST pull-downs on NSC nuclear extract mapped the interaction with Nipbl to the N-terminal part of Zfp609, which also includes the most highly conserved region. The C2H2 zinc-finger domain by itself was not sufficient for Nipbl binding.

Analogous to *Zfp609*, *Nipbl* transcripts are enriched in the ventricular zone at E14.5 (Figure S2D). Our identified direct physical association between Zfp609 and Nipbl/cohesin may suggest

CellPress

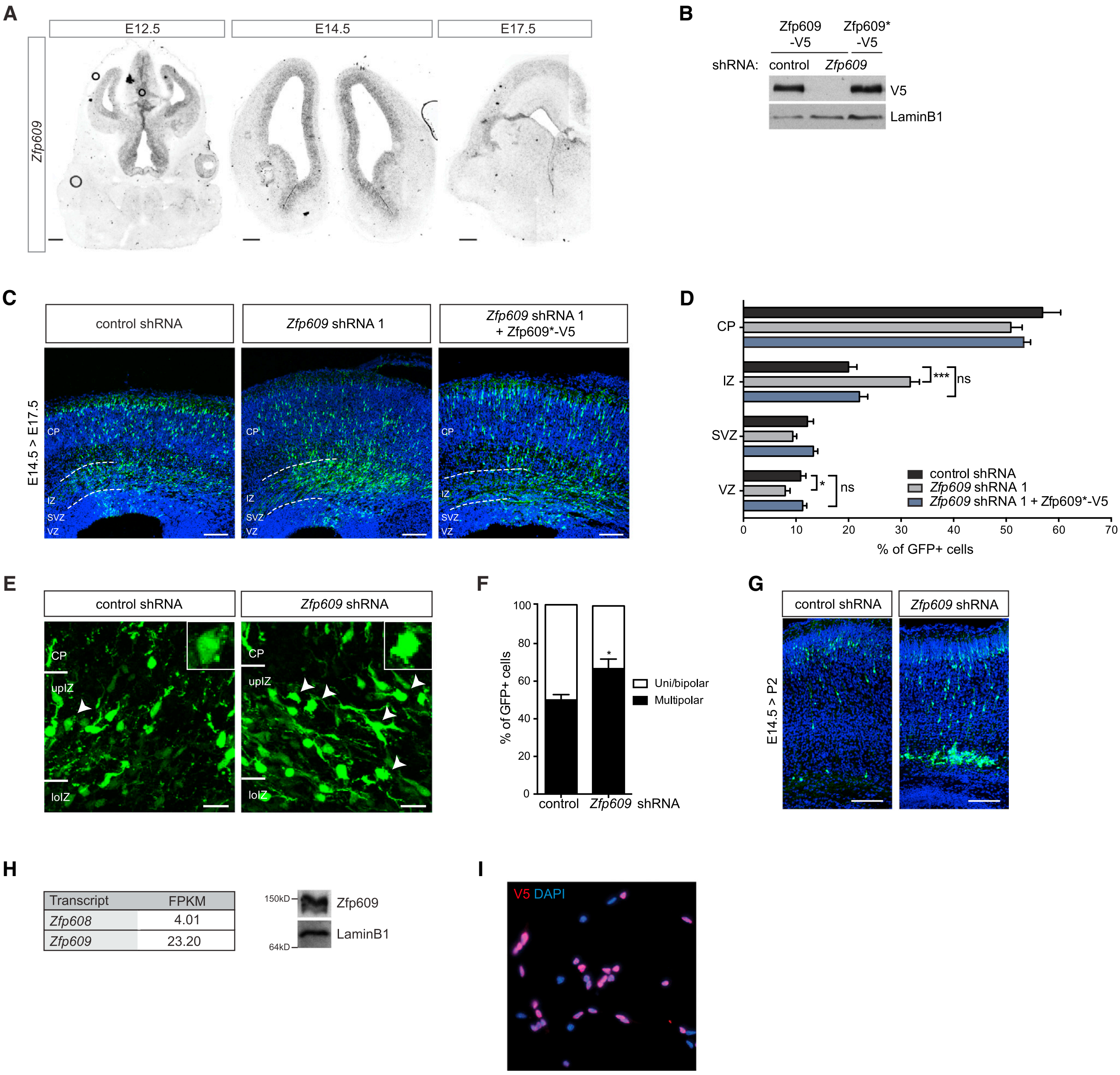

Figure 1. Zfp609 Is Expressed in Neural Progenitors and Regulates Cortical Neuron Migration

(A) Composite bright field images of in situ hybridization on cortical cryosections at indicated stages of mouse development. Scale bar represents 200 μm.

(B) Western blot with indicated antibodies on HEK293T lysates transiently transfected with wild-type or shRNA-resistant (*) Zfp609-V5 expression constructs and control or Zfp609-targeting shRNA. Lamin B1 was used as a loading control.

(C) Cryosections of mouse embryonic brains in utero electroporated with *Zfp609*-targeting shRNAs and Zfp609*-V5 rescue construct, stained with GFP to visualize transfected cells. Ventricular (VZ), subventricular (SVZ), and intermediate zones (IZ) and cortical plate (CP) are indicated. Scale bar represents 100 μm.

(D) Quantification of (C) showing percentage of GFP-expressing cells in indicated cortical regions. Error bars represent SEM, *$p < 0.05$, ***$p < 0.001$; ns, non significant; two-tailed unpaired Student's t test, n = 7.

(E) Representative images showing morphology of electroporated neurons at E17.5 near the border between IZ and CP. Arrowheads point to multipolar cells; higher magnification in inset. Scale bar represents 20 μm.

(F) Quantification of cell morphology in upper IZ. Error bars represent SEM, *$p < 0.05$, two-tailed unpaired Student's t test, n = 7 (control shRNA) and 8 (*Zfp609* shRNA).

(G) Representative images of cryosections of electroporated mouse embryonic brains at postnatal day 2, stained with GFP antibody. Scale bar represents 100 μm.

(H) Normalized expression levels in fragments per kilobase of exon per million mapped reads (FPKM) of *Zfp608* and *Zfp609* transcripts in NSCs. Western blot analysis of NSC lysate with Zfp609 antibody. Lamin B1 was used as a loading control.

(I) Immunocytochemistry with V5 antibody on NSCs showing nuclear localization of ectopically expressed Zfp609-V5.

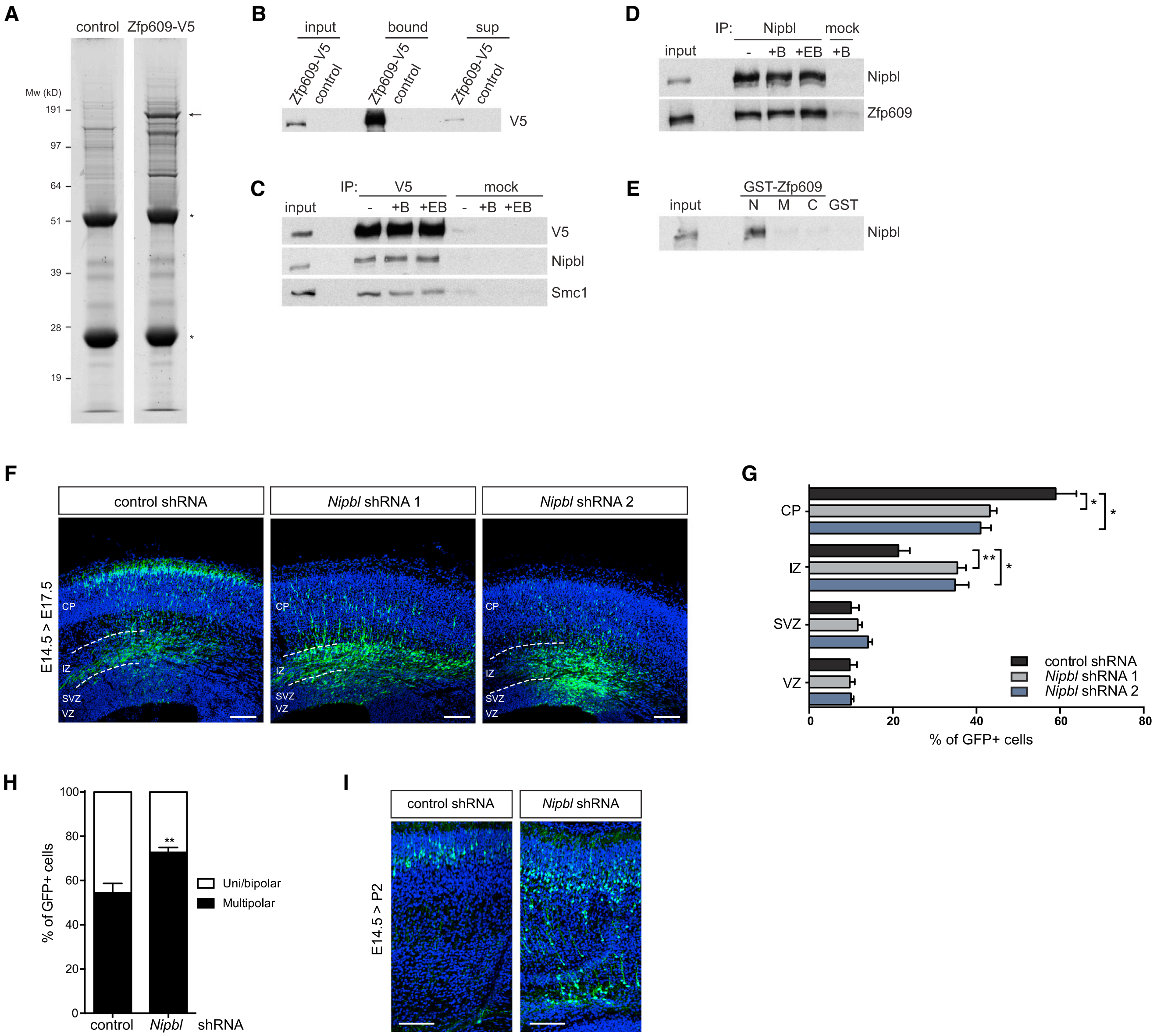

Figure 2. Nipbl Interacts with Zfp609 and Regulates Neuronal Migration

(A) Colloidal Coomassie-stained SDS-PAA gel of Zfp609-V5 and control purification. Zfp609-V5 band is indicated by an arrow. Bands representing antibody heavy and light chain are indicated by an asterisk.

(B) Western blot with V5 antibody on input, supernatant, and bound fractions of V5 affinity purification.

(C) Western blot with indicated antibodies on V5 immunoprecipitates from Zfp609-V5-expressing NSCs. Benzonase (B) or ethidium bromide (EB) was added as indicated. Normal mouse IgG was used as control.

(D) Western blot with indicated antibodies on Nipbl immunoprecipitates. Benzonase or ethidium bromide was added as indicated. Normal mouse IgG was used as control.

(E) Western blot analysis with Nipbl antibody on GST pull-down fractions from NSC nuclear extract using GST-Zfp609 N-terminal (N), middle (M), and C-terminal (C) fragments or GST control.

(F) Cryosections of mouse embryonic brains in utero electroporated with indicated *Nipbl*-targeting shRNAs, stained with GFP to visualize transfected cells. Ventricular (VZ), subventricular (SVZ), and intermediate zones (IZ) and cortical plate (CP) are indicated. Scale bar represents 100 μm.

(G) Quantification of (F) showing percentage of GFP-expressing cells in indicated cortical regions. Error bars represent SEM, *$p < 0.05$, **$p < 0.01$, two-tailed unpaired Student's t test, n = 4.

(H) Quantification of cell morphology in upper IZ. Error bars represent SEM, **$p < 0.01$, two-tailed unpaired Student's t test, n = 4 (control, *Nipbl* shRNA 1) and 5 (*Nipbl* shRNA 2).

(I) Representative images of cryosections of electroporated mouse embryonic brains at postnatal day 2, stained with GFP antibody. Scale bar represents 100 μm.

Table 1. Zfp609-Interacting Proteins as Identified by Mass Spectrometry

Protein Name	Accession Number	Mascot[a]	emPAI[b]	Unique Peptides[c]
Zfp609	UniProt: Q8BZ47	4,314	19.29	71
Cohesin Complex				
Nipbl	UniProt: Q6KCD5	2,955	1.57	63
Smc3	UniProt: Q9CW03	2,078	3.66	41
Smc1a	UniProt: Q9CU62	2,048	3.81	44
Stag2	UniProt: A2AFF6	760	0.65	16
Rad21	UniProt: Q61550	732	1.66	17
Mau2	UniProt: Q9D2X5	406	0.86	9
Integrator Complex				
Ints1	UniProt: K3W4P2	2,910	2.28	63
Ints6	UniProt: Q6PCM2	1,834	3.99	36
Ints3	UniProt: Q7TPD0	1,714	2.44	32
Ints7	UniProt: Q7TQK1	1,522	2.28	27
Asun	UniProt: Q8QZV7	1,451	5.81	29
Ints5	UniProt: Q8CHT3	993	1.22	19
Ints2	UniProt: Q80UK8	992	0.83	19
Cpsf3l	UniProt: Q9CWS4	869	2.34	19
Vwa9	UniProt: Q8R3P6	712	1.60	14
Ints8	UniProt: Q80V86	746	0.90	17
Ints9	UniProt: Q8K114	579	1.36	15
Ints12	UniProt: Q9D168	575	1.68	11
Nabp2	UniProt: E9Q199	75	0.25	2
Transcription Factors				
Rfx4	UniProt: Q7TNK1	760	1.30	18
Zbtb20	UniProt: Q8K0L9	622	0.77	11
Other				
Maged1	UniProt: Q9QYH6	824	1.10	16
Hspa2	UniProt: P17156	771	1.40	14
Dnaja2	UniProt: Q9QYJ0	312	0.73	7
Stub1	UniProt: Q9WUD1	287	1.30	8
Akap8l	UniProt: Q9R0L7	235	0.30	5
Bag5	UniProt: Q8CI32	205	0.50	6
Cnp	UniProt: P16330	164	0.34	4
Setx	UniProt: A2AKX3	120	0.04	3
Mlf2	UniProt: Q99KX1	108	0.43	3
Zcchc11	UniProt: A2A8R7	86	0.06	3

[a]Average Mascot score for the specified protein in two replicate Zfp609-V5 samples.

[b]Average emPAI score for the specified protein in two replicate Zfp609-V5 samples.

[c]Average number of unique, non-redundant peptides for the specified protein in the Zfp609-V5 sample.

they act together in the cell and therefore mediate the same phenotype. Accordingly, we assessed the effect of Nipbl depletion on neuronal migration in E14.5 NPCs. Each of two independent shRNA constructs that resulted in over 50% reduction in *Nipbl* transcript levels caused significant accumulation of targeted cells in the IZ 3 days after electroporation, accompanied by a reduction of cell numbers in the CP (Figures 2F, 2G, and S2E). Arrested neurons had an atypical multipolar morphology and resulted in white matter heterotopias at postnatal stages (3/5 mice; Figures 2H and 2I). Other aspects of neurogenesis were not notably affected by Nipbl depletion (Figures S1G–S1I). We conclude that Zfp609 and Nipbl physically interact and regulate the same cellular process during mouse forebrain development.

Zfp609 and Nipbl Co-occupy Active Promoter and Enhancer Regions

To assess if Zfp609 and Nipbl may cause the same phenotype by cooperating in regulating target genes, we first determined genomic binding sites for both factors in cultured NSCs. Chromatin immunoprecipitations of ectopically expressed Zfp609-V5 and endogenous Nipbl (Figures S3A and S3B) were analyzed by high-throughput sequencing of bound DNA (ChIP-seq). Two independent biological replicates correlated well (Pearson $r > 0.9$) and samples were pooled for further analysis. We identified 24,064 Zfp609 and 27,874 Nipbl genomic binding sites. Zfp609 and Nipbl showed a strikingly high (65%–75%) overlap in binding sites (Figure 3A). Zfp609 binding signal corresponded well to that of Nipbl (Figure S3C).

We reanalyzed published data on genome-wide binding profiles of cohesin subunit Smc1 and CTCF in NSCs (Phillips-Cremins et al., 2013). Previously, cohesin sites in enhancer and promoter regions were demonstrated to also be bound by Nipbl and the Mediator complex in ESCs (Kagey et al., 2010). We found that binding sites of Nipbl and Zfp609 hardly overlap with Smc1, while Smc1 and CTCF binding sites did extensively overlap in NSCs (Figures 3A, S3C, and S3D). A similar discrepancy between the genomic localization of cohesin and its loading factor was recently reported in human mammary epithelial HB2 cells (Zuin et al., 2014). Our results cannot be attributed to differences in antibody epitope or performance, as the antibody was identical to the one used for ChIP-seq in ESCs (Kagey et al., 2010). This suggests that also in NSCs, Nipbl can have a cohesin-independent role in transcription regulation.

Compared to cohesin, CTCF, and other NSC transcription factors (Figure 3B; Mateo et al., 2015), Nipbl and Zfp609 have a preference for promoter regions, with 30%–39% of binding sites mapping in a window from −1 kb to +1 kb around transcription start sites (TSSs). We examined the chromatin landscape surrounding Zfp609 and Nipbl binding sites by profiling their binding intensity to a catalog of NSC regulatory elements compiled based on histone modification patterns surrounding DNaseI hypersensitive sites (DHSs) (Mateo et al., 2015). We found that Nipbl and Zfp609 predominantly localize to active and poised proximal and distal DHS clusters, hallmarked by the presence of H3K27ac and by the absence of H3K27 post-translational modification, respectively (Figures 3C and 3D). Nipbl and Zfp609 promoter-bound genes have an above-average expression level (Figure 3E). In contrast, Smc1 and CTCF binding was mostly detected at DHSs that were not marked by any of the assessed histone modifications (Figures 3C and 3D).

Motif discovery analysis using MEME-ChIP (Machanick and Bailey, 2011) detected enrichment of consensus sites for Sp1

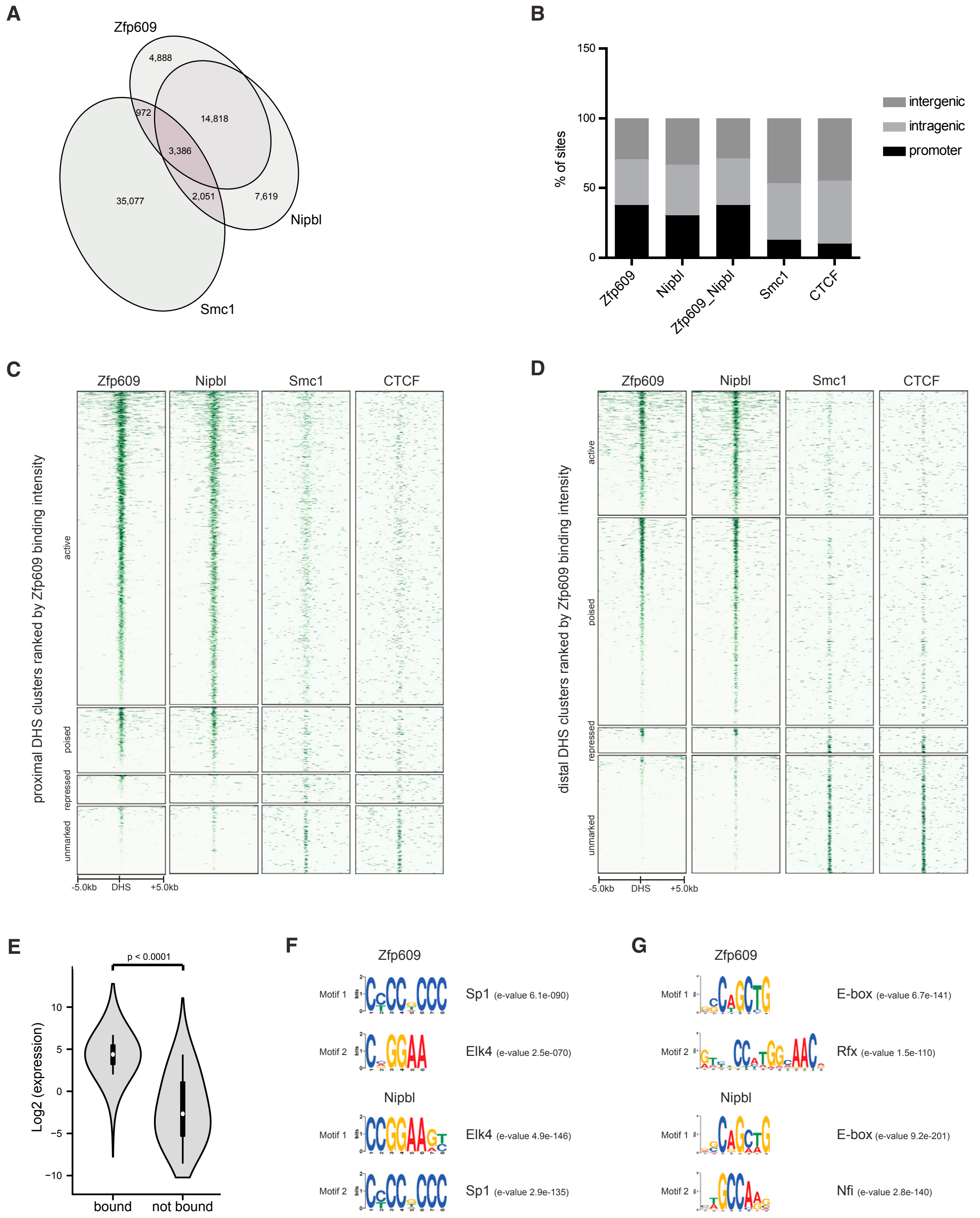

Figure 3. Zfp609 and Nipbl Co-localize to Active Promoter and Enhancer Regions

(A) Venn diagram showing overlap of Zfp609, Nipbl, and Smc1 bound regions.

(B) Distribution of Zfp609, Nipbl, Zfp609/Nipbl common, Smc1, and CTCF genomic binding sites to promoters (−1 kb to +1 kb)and intra- and intergenic regions.

(C) Heatmap of 7,030 active H3K4me3, H3K27ac-marked; 1,498 poised H3K4me1/me2-marked; 690 repressed H3K4me2, H3K27me3-marked; and 1,573 unmarked promoter proximal DNaseI hypersensitive (DHS) sites centered around DHS summits. Regions are ranked by normalized Zfp609 ChIP-seq signal, and mean ChIP-seq counts of indicated factors are plotted.

(D) Heatmap of 3,912 active H3K4me1, H3K27ac-marked; 6,487 poised H3K4me1-marked; 866 repressed H3K4me1, H3K27me3-marked; and 3,714 unmarked distal DHSs displaying 10 kb region around DHS summit. Regions are ranked by normalized Zfp609 ChIP-seq signal, and mean ChIP-seq counts of indicated factors are plotted.

(legend continued on next page)

and the Ets family transcription factor Elk4 in proximal DHSs bound by Zfp609 and Nipbl (Figure 3F). These motifs are commonly found in promoter regions (Mateo et al., 2015) and, apart from the bimodal distribution of Elk4 motifs flanking Nipbl sites, are not centrally enriched in Zfp609 or Nipbl peaks (Figure S3E), suggesting that Zfp609 or Nipbl is not targeted to promoter regions by sequence-specific transcription factors. E-box motifs recognized by bHLH transcription factors are highly abundant in active enhancer elements (Mateo et al., 2015) and were significantly enriched at the center of distal Zfp609 and Nipbl peaks (Figures 3G and S3F). In addition, we found a significant central enrichment for nuclear factor I (Nfi) and Rfx motifs. Rfx4 was detected as a Zfp609 co-purifying factor by mass spectrometry (Table 1), and although this approach did not identify bHLH transcription factors, we were able to demonstrate a specific DNA-independent interaction between V5-Ascl1 and FLAG-tagged Zfp609 (Figure S3G). Rfx and bHLH factors therefore constitute candidate-targeting factors for Zfp609 and Nipbl to distal regulatory elements.

Zfp609 and Nipbl Regulate Neuronal Migration Genes

To identify Nipbl and Zfp609 target genes that could account for the neuronal migration defects observed in vivo, we depleted both factors individually from NSCs by RNAi and identified differentially expressed genes by RNA-seq (Figures 4A, 4B, S4A, and S4B). Nipbl and Zfp609 KD significantly affected the expression of 3,748 and 1,103 genes, respectively. A much higher fraction of genes than would be expected by chance were deregulated in both KD conditions ($n = 619$, $p = 3.10 \times 10^{-215}$, hypergeometric test). Out of these, 83% changed expression in the same direction, suggesting cooperativity between Nipbl and Zfp609 in gene regulation. We subsequently focused our analysis on shared target genes of Nipbl and Zfp609, postulated as bound and regulated by both factors. To associate genes with Nipbl and Zfp609 binding sites, we used GREAT (Genomic Regions Enrichment of Annotations Tool) (McLean et al., 2010), which assigns to each gene a basal regulatory domain from −5 kb to +1 kb, extending up to 1 Mb to the next neighboring basal regulatory domains. Intersection of thus-determined bound genes with deregulated genes resulted in the identification of 490 target genes downstream of both Zfp609 and Nipbl (Figure 4C). A total of 398 target genes were misregulated in parallel, with 244 genes activated and 154 genes repressed by both factors.

Gene ontology (GO) analysis on these two categories of Zfp609/Nipbl target genes revealed enrichment for terms related to cell motion and the extensive cytoskeletal remodeling that occurs during this process (“cell projection organization,” “regulation of neuron projection development,” and “regulation of axogenesis”), in particular among the set of target genes activated by Nipbl and Zfp609 (Figures 4D and 4E). Similar GO terms were enriched among putative Zfp609 target genes that were also deregulated in E13.5 *Nipbl*$^{+/-}$ brains (Figures S4C and S4D) (Kawauchi et al., 2009). We focused in more detail on a subset of genes (i.e., *Sema3a*, *Nrp1*, *Plxnd1*, and *Gabbr2*) whose downregulation is known to cause neuronal migration defects. Sema3a is a secreted chemoattractive guidance molecule present in a descending gradient from the CP that acts through the co-receptor neuropilin-1 (Nrp1) and specific Plexin receptors, including PlexinD1 (Plxnd1), to promote radial migration of cortical neurons (Chen et al., 2008). Disruption of the gradient or downregulation of either Nrp1 or Plxnd1 results in mislocalization of cells to lower cortical regions (VZ/SVZ/IZ). In addition, non-hyperpolarizing signaling through $GABA_B$ receptors was shown to be required for cell transition from the IZ to the CP in vitro and in vivo (Behar et al., 2000, 2001; Bony et al., 2013). Indeed, we could detect Zfp609 and Nipbl binding to promoter and intragenic regions of *Sema3a*, *Nrp1*, *Plxnd1*, and *Gabbr2*, and depletion of Nipbl or Zfp609 reduced expression of these targets in NSCs (Figures 4F–4H). We therefore conclude that Zfp609 and Nipbl co-regulate genes required for cortical neuron migration.

Zfp609 and Nipbl Interact with Integrator to Regulate Migration Genes

The binding of Zfp609 and Nipbl to active promoter regions suggests they may directly contact the basal transcription machinery to activate transcription. Although we did not consistently detect RNA pol2 subunits in Zfp609 pull-downs, we did find thirteen subunits of the Integrator complex, which associates with the C-terminal domain (CTD) of RNA pol2 (Malovannaya et al., 2010) (Table 1). Specificity of the interaction of Zfp609 with the Ints1 subunit was demonstrated by detection of Ints1 by immunoblotting in the Zfp609-V5 sample and not in the control (Figure 5A).

The interaction of endogenous Zfp609, Nipbl, and Integrator complex was independently verified by their co-immunoprecipitation by an antibody against Ints1. Immunoblotting showed specific co-purification of Zfp609 and Nipbl, independent of DNA (Figure 5B). Western blotting with an antibody against Ints11 (Cpsf3l) was used as a positive control. GST pull-downs mapped the interaction domain with Integrator to the N-terminal region of Zfp609, which also brought down RNA pol2 (Figure 5C).

We determined the genomic distribution of Integrator binding by performing ChIP-seq with an antibody that efficiently brought down chromatin-bound Ints11 (Figure S5A). Three independent replicates correlated well (Pearson $r > 0.86$) and were pooled for downstream analysis. Consistent with published reports from HeLa cells, we detected widespread binding to promoter and predominantly active enhancer regions, where Integrator strongly co-localizes with Zfp609 and Nipbl (Figures 5D, 5E, and S5C) (Gardini et al., 2014; Lai et al., 2015). For comparison, we included genome-wide ChIP-seq data for RNA pol2, showing its preferential binding to active promoter regions (Figures 5D, 5E, and S5B).

Integrator was recently shown to physically associate with negative elongation factor (NELF) and the DRB-sensitivity

(E) Violin plot showing distribution of log2 transformed absolute expression values of genes either bound or not bound in their promoter region by Zfp609 and Nipbl. White dot indicates the median and thick black bar represents the interquartile range. p value by Mann-Whitney test is indicated.
(F) Top two most significantly enriched motifs in Zfp609 and Nipbl proximal binding peaks. Enrichment values as reported by Centrimo are listed.
(G) Top two most significantly enriched motifs in distal Zfp609 and Nipbl peaks. Enrichment values as reported by Centrimo are listed.

CellPress

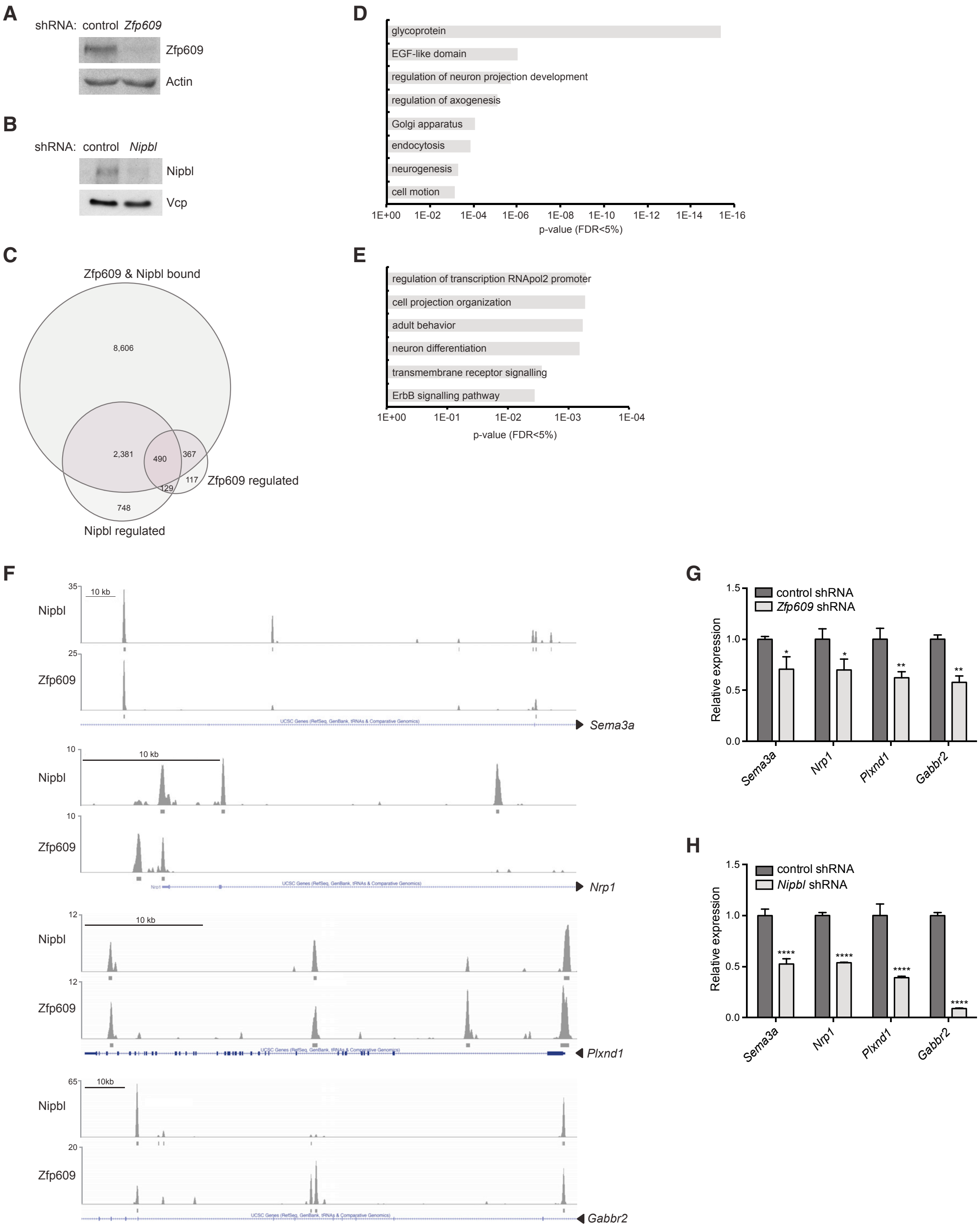

Figure 4. Zfp609 and Nipbl Regulate Neuronal Migration Genes

(A) Western blot with Zfp609 antibody on NSC lysates lentivirally transduced with control or *Zfp609*-targeting shRNA. Actin was used as a loading control.

(B) Western blot with Nipbl antibody on NSC lysates lentivirally transduced with control or *Nipbl*-targeting shRNA. Vcp was used as a loading control.

(C) Venn diagram showing intersection of genes bound in their regulatory region (basal −5 kb to +1 kb plus extension up to 1 Mb, GREAT) and transcriptionally regulated by Zfp609 and Nipbl.

(D) GO analysis on genes bound and activated by Zfp609 and Nipbl. DAVID p values are shown, FDR < 5%.

(E) GO analysis on genes bound and repressed by Zfp609 and Nipbl. DAVID p values are shown, FDR < 5%.

(legend continued on next page)

inducing factor (DSIF) complex and to affect RNA pol2 pause release at coding genes (Gardini et al., 2014; Skaar et al., 2015; Stadelmayer et al., 2014; Yamamoto et al., 2014). We therefore asked whether paused RNA pol2 also constitutes a key feature of genes containing Zfp609 and Nipbl binding sites in their promoter proximal regions. Indeed, when we compared RNA pol2 pausing indices of Zfp609/Nipbl-bound versus all non-bound expressed genes (fragments per kilobase of transcript per million mapped reads [FPKM] > 1), we could detect significantly increased pausing at Zfp609/Nipbl promoter-bound genes (Figure 5F).

To determine genes co-regulated by Zfp609, Nipbl, and Integrator, we performed RNA-seq analysis on NSCs depleted for Integrator subunits Ints1 and Ints11 by RNAi (Figures 5G and S5D). All of the 142 common putative target genes were deregulated in the same direction by either Ints1 or Ints11 KD, suggesting both factors function agonistically within the Integrator complex (data not shown). Integrator-regulated genes were pooled and compared to the previously classified set of Nipbl and Zfp609 target genes (Figure 5H). In total, 85% of common deregulated genes changed expression in the same direction upon either Zfp609/Nipbl or Integrator KD, suggesting functional cooperativity. Two-thirds of these were downregulated in all KD conditions. Three out of the four genes that we previously implicated in the regulation of cortical neuron migration downstream of Zfp609 and Nipbl, i.e., *Sema3a*, *Plxnd1*, and *Gabbr2*, were downregulated upon Ints1 depletion (Figure 5I). Importantly, compromising Integrator complex function by in utero electroporation of either *Ints1*- or *Ints11*-targeting shRNAs also resulted in an abnormal accumulation of cells in the IZ (Figures 5J and 5K). We therefore conclude that Zfp609 and Nipbl act with the Integrator complex to positively regulate common target genes and thereby promote neuronal migration.

DISCUSSION

In an unbiased proteomics approach, we identified physical interactions between Zfp609, cohesin, and the cohesin loading factor Nipbl/Mau2. We show that in mice, the two *Drosophila scribbler* homologs *Zfp608* and *Zfp609* are expressed in the embryonic forebrain in a mutually exclusive manner, possibly through direct cross-repression, analogous to what was reported in developing thymocytes (Reed et al., 2013). Indeed, we find that Zfp609 and Nipbl bind to the *Zfp608* promoter in NSCs to silence its transcription. Interestingly, elevated *Zfp608* expression was identified as one of three biomarkers that could accurately distinguish CdLS probands from healthy control individuals (Liu et al., 2009), implicating these factors and their interrelationship in the establishment of CdLS.

We mapped the genomic binding sites of Zfp609 and Nipbl in NSCs to predominantly active promoter and enhancer regions, similar to the genomic distribution of Nipbl in ESCs (Kagey et al., 2010). Contrary to ESCs and despite the significantly higher number of Nipbl peaks identified here, very few Nipbl sites were co-occupied by cohesin. Instead, cohesin almost exclusively localizes to CTCF sites, perhaps as a consequence of cohesin sliding, analogous to reports in yeast (Lengronne et al., 2004). This discrepancy between ESCs and NSCs therefore may reflect a difference in cell-cycle length or cohesin dynamics, which impact the frequency of cohesin complex reloading. Interestingly, a similar disparity in Nipbl and cohesin localization was noted in human breast epithelial cells, which, in combination with differential effects on gene expression, led the authors to propose a cohesin-independent role for Nipbl in gene regulation (Zuin et al., 2014). How Nipbl would act as a transcription factor remained unclear. We now provide evidence that Nipbl contacts the Integrator complex associated with the RNA pol2 CTD, possibly via Zfp609.

Genome-wide ChIP-seq analysis demonstrated that virtually all active and 50% of poised NSC promoters are bound by Integrator, where it often co-localizes with Zfp609/Nipbl. Integrator interacts with the pause-inducing factors NELF and DSIF and occupies promoters containing paused RNA pol2 in HeLa cells (Stadelmayer et al., 2014). Similarly, Zfp609 and Nipbl-bound NSC promoters are characterized by a higher mean RNA pol2 pausing index. RNA pol2 pausing occurs after the first 20–60 nucleotides have been transcribed and provides an additional mode of regulation for nearly 50% of mammalian genes (Kwak and Lis, 2013).

The role of Integrator in the regulation of RNA pol2 pausing appears to be two sided, as it has been shown to be required both for maintenance of pausing and for transition into productive elongation (Gardini et al., 2014; Stadelmayer et al., 2014). Integrator promotes RNA pol2 release at immediate early genes in HeLa cells by recruiting the super elongation complex (SEC), containing AFF4 and the most active form of positive transcription elongation factor P-TEFb (Luo et al., 2012). De novo gain-of-function mutations in *AFF4* were recently identified in three patients with a new syndrome, CHOPS (cognitive development and coarse facies, heart defects, obesity, pulmonary involvement, short stature, and skeletal dysplasia), that displays phenotypic overlap with CdLS (Izumi et al., 2015). We provide biochemical evidence for a link between the most frequently mutated gene in CdLS, *NIPBL*, and the AFF4-containing SEC, implicating the regulation of transcription elongation in the ontogeny of CdLS abnormalities.

We show that depletion of Zfp609, Nipbl, or Integrator in vivo results in aberrant neuronal migration and postulate that the deregulated expression of their target genes involved in semaphorin and GABA signaling is likely to be responsible for this phenotype. *Zfp609* and *Nipbl* transcripts are mostly present in the VZ/SVZ, while the migration arrest occurs in the IZ. This delay

(F) UCSC browser tracks displaying normalized Nipbl and Zfp609 ChIP-seq signal in intragenic and promoter regions of indicated genes. Significant called peaks are indicated on a separate line.

(G) qPCR analysis on NSCs lentivirally transduced with control or *Zfp609*-targeting shRNA. Error bars represent SEM, *p < 0.05, **p < 0.01, unpaired Student's t test corrected for multiple comparisons using Holm-Sidak method, n = 3.

(H) qPCR analysis on NSCs lentivirally transduced with control or *Nipbl*-targeting shRNA. Error bars represent SEM, ****p < 0.0001, unpaired Student's t test corrected for multiple comparisons using Holm-Sidak method, n = 3.

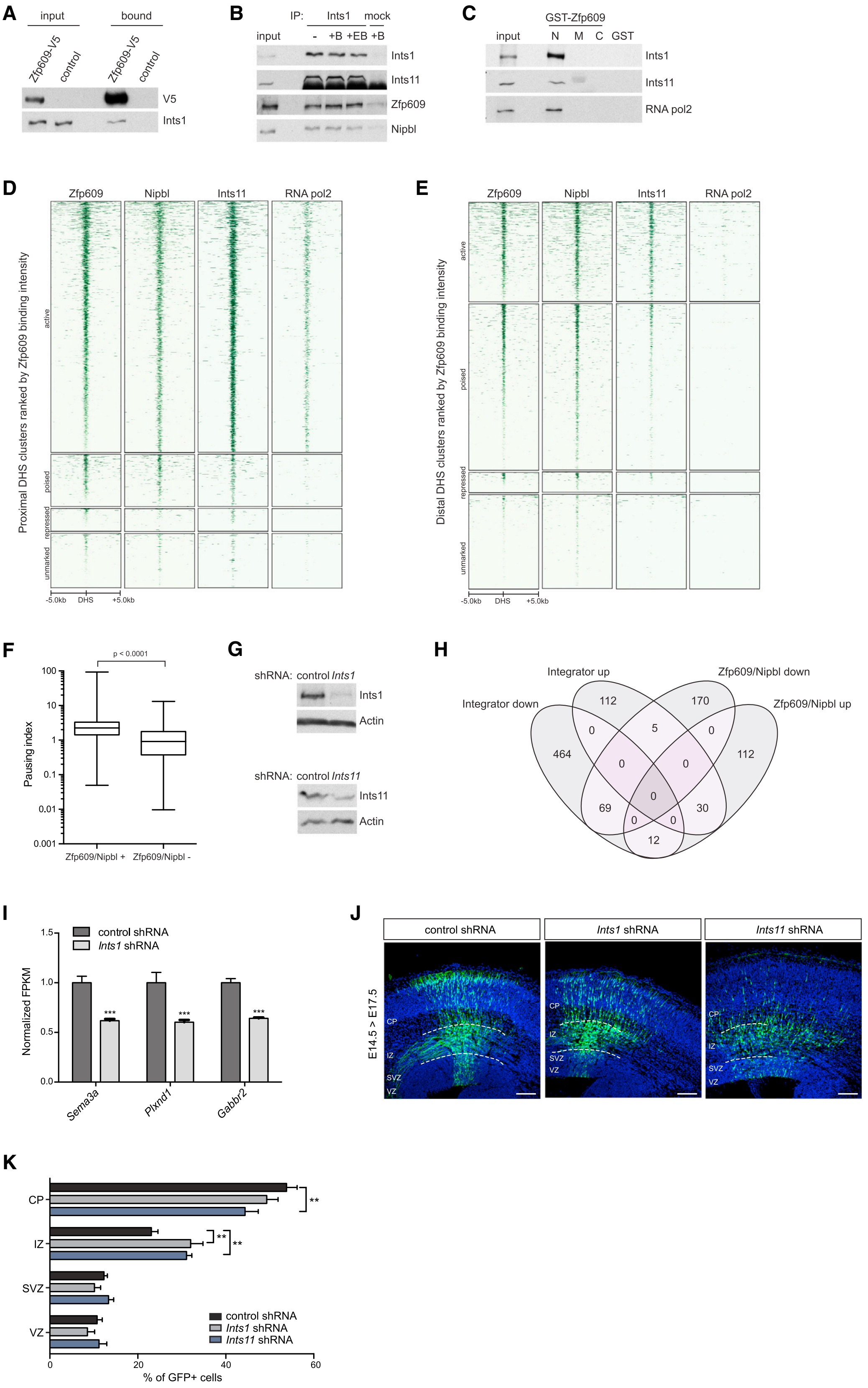

(legend on next page)

could reflect a requirement for Zfp609 and Nipbl to establish and maintain accessibility of genomic binding sites to other regulatory factors. Alternatively, Zfp609 and Nipbl protein expression might be maintained in postmitotic neurons after downregulation of their respective transcripts.

Defects in neuronal migration and the subsequent incorrect neuronal positioning lead to disruption of neural circuit formation and have been causally linked to intellectual disability and seizures, which are both features of CdLS (Liu and Krantz, 2009; Verrotti et al., 2013). Indeed, neuronal heterotopias were reported in autopsy data of CdLS patients (Tekin and Bodurtha, 2015). Abnormal localization of E13.5-born neurons in the IZ was also observed in mice carrying a heterozygous mutation in *Ankrd11*, a chromatin regulator mutated in rare cases of CdLS (Gallagher et al., 2015; Parenti et al., 2016). Furthermore, our data suggest that Zfp609 and Nipbl act through the Integrator complex to contact the basal transcription machinery and regulate gene expression at the level of RNA pol2 pause release. Embryonic brain KD of Phf6 or its interactor Paf1, a recently identified regulator of promoter-proximal RNA pol2 pausing, resulted in aberrant neuronal accumulation in the IZ caused by downregulation of Cspg5, a transmembrane glycoprotein of the neuregulin family (Chen et al., 2015; Zhang et al., 2013). Importantly, mutations in *PHF6* cause Börjeson-Forssman-Lehmann syndrome (BFLS), characterized by moderate-to-severe intellectual disability and seizures (Lower et al., 2002). Together with our data, this suggests a prominent role for the regulation of RNA pol2 pause release in the control of neuronal migration, which ultimately impacts cognitive function.

By studying Nipbl function in neural progenitors in vitro and in vivo, we have generated a deeper understanding of its gene regulatory network and uncovered a role in the control of neuronal migration, which, when perturbed, is likely to contribute to the cognitive impairment of CdLS patients. Our description of the molecular machinery involved in transcription regulation by Nipbl in neural progenitors offers a range of candidates for mutation screening in the 30% of CdLS cases where no causative mutation has been identified.

EXPERIMENTAL PROCEDURES

Cell Culture

NS5 NSCs were grown on laminin-coated dishes as previously described (Conti et al., 2005). To generate inducible V5-tagged Zfp609-expressing NSCs, the coding sequence of Zfp609 fused to a C-terminal V5 tag was inserted into lentiviral plasmid Tet-O-FUW-EGFP (kind gift from Marius Wernig, Addgene #30130; Vierbuchen et al., 2010) in place of eGFP. Lentiviral particles were produced by co-transfection with psPax2 and pMD2.G (kind gifts from Didier Trono, Addgene #12260 and #12259) in HEK293T cells and concentrated by ultracentrifugation. NSCs were simultaneously transduced with lentiviruses for Zfp609-V5 and rtTA (kind gift from Rudolf Jaenisch, Addgene #20342; Hockemeyer et al., 2008). Expression of Zfp609-V5 was induced for a minimum of 6 hr by addition of 1 μg/mL doxycycline (Sigma). HEK293T and P19 cells in standard culture conditions were transiently transfected using Lipofectamine 2000 transfection reagent (Invitrogen).

Antibodies

Zfp609 antibodies were generated in guinea pigs against recombinantly expressed Zfp609 (aa 1–282) fused to GST. Normal mouse IgG (sc-2025), normal rabbit IgG (sc-2027), and antibodies against Lamin B1 (sc-6216 and sc-6217), RNA pol2 (sc-899), and GFP (sc-8334) were obtained from Santa Cruz Biotechnology. Antibodies against Smc1 (A300-055A), Nipbl (A301-779A), Int1 (A300-361A), and Int11 (A301-274A) were obtained from Bethyl Laboratories. Additional antibodies included V5 (R960-25, Invitrogen), Actin (A2066, Sigma), Vcp (ab11433, Abcam), and GFP (4745-1051, AbD Serotec).

Protein Purification

Control and Zfp609-V5-expressing NSCs were expanded to ten confluent 14 cm diameter dishes (2×10^8 cells) and scraped in ice-cold PBS, and nuclear extracts were prepared (Dignam et al., 1983) and diluted to 100 mM NaCl with C-0 (20 mM HEPES [pH 7.6], 0.2 mM EDTA, 1.5 mM $MgCl_2$, and 20% glycerol). Complete EDTA-free protease inhibitors (Roche) were added to all buffers. A total of 40 μL anti-V5 agarose beads (Sigma) were equilibrated in buffer C-100* (20 mM HEPES [pH 7.6], 100 mM KCl, 0.2 mM EDTA, 1.5 mM $MgCl_2$, 0.02% NP-40, and 20% glycerol); blocked in 0.2 mg/mL chicken egg albumin (Sigma), 0.1 mg/mL insulin (Sigma), and 1% fish skin gelatin (Sigma) in C-100*; and added to 1.5 mL nuclear extract in no stick microtubes (Alpha Laboratories) for 3 hr at 4°C in the presence of 225 U Benzonase (Novagen). Beads were washed five times for 5 min with C-100* and boiled in 30 μL SDS loading buffer. Eluted proteins separated by polyacrylamide gel electrophoresis were stained with Colloidal Coomassie (Invitrogen), and entire gel lanes were analyzed by mass spectrometry as previously described (van den Berg et al., 2010). Criteria for inclusion in Table 1 are presence in both purifications with a Mascot score of at least 50 and both Mascot and emPAI

Figure 5. Zfp609 and Nipbl Interact with Integrator to Regulate Cortical Migration

(A) Western blot with indicated antibodies of V5 immunoprecipitates on Zfp609-V5-expressing or control NSC nuclear extract.

(B) Immunoprecipitation of Ints1 analyzed by western blot with indicated antibodies. Benzonase (B) or ethidium bromide (EB) was added as indicated. Rabbit anti-GFP was used as control.

(C) GST pull-down with Zfp609 N-terminal (N), middle (M), and C-terminal (C) fragments or GST control on NSC nuclear extract analyzed by western blot with indicated antibodies.

(D) Heatmap of 7,030 active, 1,498 poised, 690 repressed, and 1,573 unmarked promoter proximal DHSs displaying 10 kb around DHS summits. Regions are ranked by normalized Zfp609 ChIP-seq signal, and mean ChIP-seq counts of indicated factors are plotted.

(E) Heatmap of 3,912 active, 6,487 poised, 866 repressed, and 3,714 unmarked distal DHSs displaying 10 kb region around DHS summit. Regions are ranked by normalized Zfp609 ChIP-seq signal, and mean ChIP-seq counts of indicated factors are plotted.

(F) Boxplot representing distribution of pausing indices of Zfp609 TSS-bound (n = 5,391) versus all other (n = 1,543) expressed (FPKM > 1) genes. Whiskers represent minimum and maximum values. p value by Mann-Whitney test is indicated.

(G) Western blot on NSC lysates lentivirally transduced with the indicated shRNAs. Actin was used as a loading control.

(H) Venn diagram displaying intersection of deregulated genes in Integrator KD with Zfp609/Nipbl target genes.

(I) Normalized expression values from RNA-seq data on control or Ints1-depleted NSCs. Error bars represent SEM, ***$p < 0.001$, unpaired Student's t test corrected for multiple comparisons using Holm-Sidak method, n = 3.

(J) Cryosections of mouse embryonic brains in utero electroporated with *Ints1*- and *Ints11 (Cpsf3l)*-targeting shRNAs, stained with GFP to visualize transfected cells. Ventricular (VZ), subventricular (SVZ), and intermediate zones (IZ) and cortical plate (CP) are indicated. Scale bar represents 100 μm.

(K) Quantification of (J) showing percentage of GFP-expressing cells in indicated cortical regions. Error bars represent SEM, **$p < 0.01$, two-tailed unpaired Student's t test corrected for multiple comparisons using Holm-Sidak method, n = 4 (control) and 6 (*Ints1* and *Ints11* shRNA).

scores at least 3-fold enriched over the corresponding control purification. Cytoskeletal and cytoplasmic proteins were removed from the analysis. For small-scale immunoprecipitations, 2.5 μg antibody, 25 μL protein A or protein G dynabeads, and 200 μL nuclear extract were used. Normal mouse IgG, rabbit IgG, or anti-GFP antibody served as control, and 25 U Benzonase or 25 μg/mL ethidium bromide were added where indicated.

GST Pull-Down

Zfp609 fragments were cloned into pGEX-2TK. GST-fusion proteins and GST were expressed in BL21-CodonPlus (DE3)-RP competent cells, and GST pull-downs were performed as previously described (van den Berg et al., 2010).

RNAi and RNA-Seq

Short hairpin sequences (Table S3) were cloned into pSuper (Oligoengine) for transient transfections and in utero electroporation purposes. For RNAi in NSCs, short hairpin sequences were cloned from pENTR/pSUPER+ into pLentiX1 (kind gifts from Eric Campeau, Addgene #17338 and #17297; Campeau et al., 2009). Lentiviral particles were produced in HEK293T cells, concentrated by ultracentrifugation, and used to infect NSCs. Transduced NSCs were selected for 48 hr with 0.5 μg/mL puromycin starting 24 hr after transduction, and RNA was extracted using Trizol reagent (Invitrogen) and purified on RNeasy columns (QIAGEN). Sequencing libraries were prepared according to the TruSeq RNA Sample Preparation v2 kit (Illumina) and sequenced with the HiSeq 2000 (Illumina). Reads were aligned to the mm9 mouse genome with TopHat, and differentially expressed genes listed in Table S2 were identified with Cuffdiff using default parameters (Trapnell et al., 2012). Primer sequences (Table S4) and Taqman probes used for validation by qPCR are listed in the Supplemental Experimental Procedures. DAVID functional clustering webtool (Huang et al., 2009) was used for GO analysis setting a false discovery rate (FDR) $< 5\%$.

ChIP-Seq Analysis and Data Visualization

For V5, Nipbl, and Ints11 ChIP, NSCs suspended in PBS were crosslinked sequentially for 45 min with 2 mM disuccinimidyl glutarate (DSG) and for 10 min with 1% formaldehyde. Reactions were quenched with 125 mM glycine, chromatin was prepared, and ChIP performed as described (Boyer et al., 2005). RNA pol2 ChIP was performed on formaldehyde-crosslinked chromatin as described (Rahl et al., 2010). Sequencing libraries were prepared from 2–10 ng ChIP or input control DNA according to Illumina standard ChIP-Seq Sample Prep kit and sequenced with a Genome Analyzer IIx or HiSeq 2000 (Illumina). Reads were aligned to the mm9 mouse genome using Bowtie 2 (Langmead and Salzberg, 2012) with default parameters. Aligned reads were subsampled with custom python scripts (Mateo et al., 2015) and peaks called with MACS version 2 (Zhang et al., 2008) setting shift size to 90 and q value to 0.05. ChIP bedGraphs uploaded to UCSC Genome browser mm9 were normalized to control and converted to bigWig using MACS. Venn diagrams were generated using Biovenn (Hulsen et al., 2008) and eulerAPE (Micallef and Rodgers, 2014). Heatmaps were generated from MACS q value bigWig files using the deepTools package (Ramírez et al., 2014). Mean scores of 100 bp bins 10 kb around the peak or DHS summit were displayed. Motif discovery was performed with MEME-ChIP (Machanick and Bailey, 2011) using 400 bases around the peak summit as input. RNA pol2 pausing indices were calculated as the ratio of reads in the TSS proximal region (−250 to +250 bp) over reads in the gene body (+500 bp to +2,500 bp). Genes with no reads in either promoter or gene body region were excluded from the analysis. Primer sequences used for ChIP-qPCR analysis are listed in the Supplemental Experimental Procedures.

In Utero Electroporation

Mice were housed, bred, and treated according to guidelines approved by the Home Office under the Animal (Scientific Procedures) Act 1986. Experiments were approved by the Crick Animal Welfare and Ethical Review Body and the Home Office (project license 707644).In utero electroporation was performed essentially as described (Azzarelli et al., 2014). Briefly, 1 μg endofree plasmid preparations of pSuper shRNA constructs, pcDNA expression vector, and pCA-b-EGFPm5 silencer3 containing 0.05% Fast Green dye (Sigma) were injected into the lateral ventricle of E14.5 embryos. Five 30 V electric pulses were applied at 1 s intervals across the uterine wall using a 5 mm platinum electrode. Three days after electroporation, embryonic brains were fixed for 1 hr in 4% paraformaldehyde, cryo-protected in 30% sucrose, and embedded in OCT compound (VWR) containing 30% sucrose. Cryosections (14 μm) were stained with anti-GFP (AbD Serotec) and Alexa 488 conjugated secondary antibody and imaged with a confocal microscope. The onset of glia-guided locomotion was used to define the border between IZ and CP (Heng et al., 2010). At least 250 GFP-labeled cells per brain, irrespective of GFP level, were counted blindly; at least four brains were analyzed per condition. The numbers of uni/bipolar (i.e., one to two primary processes) and multipolar (i.e., >two primary processes) were quantified in the upper IZ ($n \geq 20$) and CP ($n \geq 70$); at least three brains were analyzed per condition.

In Situ Hybridization

Cryosections were post-fixed in 4% paraformaldehyde, treated for 10 min with 100 mM acetylated triethanolamine (pH 8.0), and pre-hybridized in hybridization buffer (50% formamide, 5× SSC, 5% SDS, and 1 mg/mL yeast tRNA) for 1 hr at 70°C. The Zfp609 probe template was PCR amplified from NSC cDNA (primer sequences in Table S5), in vitro transcribed in the presence of DIG RNA labeling mix (Roche), purified with RNeasy kit (QIAGEN), denatured, and hybridized to cryosections in hybridization buffer overnight at 70°C. Bound probes were detected with an alkaline phosphatase-conjugated anti-DIG antibody (Roche) and NBT/BCIP substrate (Sigma).

ACCESSION NUMBERS

ChIP- and RNA-seq data have been deposited in the European Nucleotide Archive under ID code ENA: ERP013418.

SUPPLEMENTAL INFORMATION

Supplemental Information includes Supplemental Experimental Procedures, five figures, and five tables and can be found with this article online at http://dx.doi.org/10.1016/j.neuron.2016.11.047.

AUTHOR CONTRIBUTIONS

Conceptualization, D.L.C.v.d.B.; Investigation, D.L.C.v.d.B., R.A., K.O., D.H.W.D., and J.A.D.; Resources, N.U. and B.M.; Writing – Original Draft, D.L.C.v.d.B.; Writing – Review & Editing, D.L.C.v.d.B. and F.G.; Funding Acquisition, D.L.C.v.d.B. and F.G.

ACKNOWLEDGMENTS

We thank Abdul K. Sesay and the high-throughput sequencing facility for assistance with ChIP-seq and RNA-seq; Juan L. Mateo, Aengus Stewart, and Richard Mittel for advice on bioinformatics analysis; Lan Chen for technical assistance; and members of the F.G. laboratory for their comments and suggestions. We thank Raymond Poot and Frank Uhlmann for suggestions that improved the manuscript. D.L.C.v.d.B. was supported by a FEBS Long-Term Fellowship. This work is part of the project Proteins at Work, a program of the Netherlands Proteomics Centre financed by the Netherlands Organization for Scientific Research (NWO) as part of the National Roadmap Large-Scale Research Facilities of the Netherlands (project number 184.032.201). The work was supported by the Francis Crick Institute, which receives its core funding from Cancer Research UK (FC0010089), the UK Medical Research Council (FC0010089), and the Wellcome Trust (FC0010089); by the UK Biotechnology and Biological Sciences Research Council (project grant BB/K005316/1 to F.G.); by the UK Medical Research Council (project grant U117570528 to F.G.); and by the Wellcome Trust (investigator award 106187/Z/14/Z to F.G.).

Received: March 14, 2016
Revised: October 10, 2016
Accepted: November 17, 2016
Published: December 29, 2016

REFERENCES

Ayoub, A.E., Oh, S., Xie, Y., Leng, J., Cotney, J., Dominguez, M.H., Noonan, J.P., and Rakic, P. (2011). Transcriptional programs in transient embryonic zones of the cerebral cortex defined by high-resolution mRNA sequencing. Proc. Natl. Acad. Sci. USA *108*, 14950–14955.

Azzarelli, R., Pacary, E., Garg, R., Garcez, P., van den Berg, D., Riou, P., Ridley, A.J., Friedel, R.H., Parsons, M., and Guillemot, F. (2014). An antagonistic interaction between PlexinB2 and Rnd3 controls RhoA activity and cortical neuron migration. Nat. Commun. *5*, 3405.

Behar, T.N., Schaffner, A.E., Scott, C.A., Greene, C.L., and Barker, J.L. (2000). GABA receptor antagonists modulate postmitotic cell migration in slice cultures of embryonic rat cortex. Cereb. Cortex *10*, 899–909.

Behar, T.N., Smith, S.V., Kennedy, R.T., McKenzie, J.M., Maric, I., and Barker, J.L. (2001). GABA(B) receptors mediate motility signals for migrating embryonic cortical cells. Cereb. Cortex *11*, 744–753.

Bony, G., Szczurkowska, J., Tamagno, I., Shelly, M., Contestabile, A., and Cancedda, L. (2013). Non-hyperpolarizing GABAB receptor activation regulates neuronal migration and neurite growth and specification by cAMP/LKB1. Nat. Commun. *4*, 1800.

Boyer, L.A., Lee, T.I., Cole, M.F., Johnstone, S.E., Levine, S.S., Zucker, J.P., Guenther, M.G., Kumar, R.M., Murray, H.L., Jenner, R.G., et al. (2005). Core transcriptional regulatory circuitry in human embryonic stem cells. Cell *122*, 947–956.

Braunholz, D., Obieglo, C., Parenti, I., Pozojevic, J., Eckhold, J., Reiz, B., Braenne, I., Wendt, K.S., Watrin, E., Vodopiutz, J., et al. (2015). Hidden mutations in Cornelia de Lange syndrome limitations of sanger sequencing in molecular diagnostics. Hum. Mutat. *36*, 26–29.

Campeau, E., Ruhl, V.E., Rodier, F., Smith, C.L., Rahmberg, B.L., Fuss, J.O., Campisi, J., Yaswen, P., Cooper, P.K., and Kaufman, P.D. (2009). A versatile viral system for expression and depletion of proteins in mammalian cells. PLoS ONE *4*, e6529.

Castronovo, P., Gervasini, C., Cereda, A., Masciadri, M., Milani, D., Russo, S., Selicorni, A., and Larizza, L. (2009). Premature chromatid separation is not a useful diagnostic marker for Cornelia de Lange syndrome. Chromosome Res. *17*, 763–771.

Chen, G., Sima, J., Jin, M., Wang, K.Y., Xue, X.J., Zheng, W., Ding, Y.Q., and Yuan, X.B. (2008). Semaphorin-3A guides radial migration of cortical neurons during development. Nat. Neurosci. *11*, 36–44.

Chen, F.X., Woodfin, A.R., Gardini, A., Rickels, R.A., Marshall, S.A., Smith, E.R., Shiekhattar, R., and Shilatifard, A. (2015). PAF1, a molecular regulator of promoter-proximal pausing by RNA polymerase II. Cell *162*, 1003–1015.

Conti, L., Pollard, S.M., Gorba, T., Reitano, E., Toselli, M., Biella, G., Sun, Y., Sanzone, S., Ying, Q.L., Cattaneo, E., and Smith, A. (2005). Niche-independent symmetrical self-renewal of a mammalian tissue stem cell. PLoS Biol. *3*, e283.

Deardorff, M.A., Bando, M., Nakato, R., Watrin, E., Itoh, T., Minamino, M., Saitoh, K., Komata, M., Katou, Y., Clark, D., et al. (2012). HDAC8 mutations in Cornelia de Lange syndrome affect the cohesin acetylation cycle. Nature *489*, 313–317.

Dignam, J.D., Lebovitz, R.M., and Roeder, R.G. (1983). Accurate transcription initiation by RNA polymerase II in a soluble extract from isolated mammalian nuclei. Nucleic Acids Res. *11*, 1475–1489.

Gallagher, D., Voronova, A., Zander, M.A., Cancino, G.I., Bramall, A., Krause, M.P., Abad, C., Tekin, M., Neilsen, P.M., Callen, D.F., et al. (2015). Ankrd11 is a chromatin regulator involved in autism that is essential for neural development. Dev. Cell *32*, 31–42.

Gardini, A., Baillat, D., Cesaroni, M., Hu, D., Marinis, J.M., Wagner, E.J., Lazar, M.A., Shilatifard, A., and Shiekhattar, R. (2014). Integrator regulates transcriptional initiation and pause release following activation. Mol. Cell *56*, 128–139.

Guerrini, R., and Parrini, E. (2010). Neuronal migration disorders. Neurobiol. Dis. *38*, 154–166.

Haecker, A., Qi, D., Lilja, T., Moussian, B., Andrioli, L.P., Luschnig, S., and Mannervik, M. (2007). Drosophila brakeless interacts with atrophin and is required for tailless-mediated transcriptional repression in early embryos. PLoS Biol. *5*, e145.

Heng, J.I., Nguyen, L., Castro, D.S., Zimmer, C., Wildner, H., Armant, O., Skowronska-Krawczyk, D., Bedogni, F., Matter, J.M., Hevner, R., and Guillemot, F. (2008). Neurogenin 2 controls cortical neuron migration through regulation of Rnd2. Nature *455*, 114–118.

Heng, J.I., Chariot, A., and Nguyen, L. (2010). Molecular layers underlying cytoskeletal remodelling during cortical development. Trends Neurosci. *33*, 38–47.

Hockemeyer, D., Soldner, F., Cook, E.G., Gao, Q., Mitalipova, M., and Jaenisch, R. (2008). A drug-inducible system for direct reprogramming of human somatic cells to pluripotency. Cell Stem Cell *3*, 346–353.

Huang, W., Sherman, B.T., and Lempicki, R.A. (2009). Systematic and integrative analysis of large gene lists using DAVID bioinformatics resources. Nat. Protoc. *4*, 44–57.

Hulsen, T., de Vlieg, J., and Alkema, W. (2008). BioVenn—a web application for the comparison and visualization of biological lists using area-proportional Venn diagrams. BMC Genomics *9*, 488.

Ishihama, Y., Oda, Y., Tabata, T., Sato, T., Nagasu, T., Rappsilber, J., and Mann, M. (2005). Exponentially modified protein abundance index (emPAI) for estimation of absolute protein amount in proteomics by the number of sequenced peptides per protein. Mol. Cell. Proteomics *4*, 1265–1272.

Izumi, K., Nakato, R., Zhang, Z., Edmondson, A.C., Noon, S., Dulik, M.C., Rajagopalan, R., Venditti, C.P., Gripp, K., Samanich, J., et al. (2015). Germline gain-of-function mutations in AFF4 cause a developmental syndrome functionally linking the super elongation complex and cohesin. Nat. Genet. *47*, 338–344.

Kagey, M.H., Newman, J.J., Bilodeau, S., Zhan, Y., Orlando, D.A., van Berkum, N.L., Ebmeier, C.C., Goossens, J., Rahl, P.B., Levine, S.S., et al. (2010). Mediator and cohesin connect gene expression and chromatin architecture. Nature *467*, 430–435.

Kawauchi, S., Calof, A.L., Santos, R., Lopez-Burks, M.E., Young, C.M., Hoang, M.P., Chua, A., Lao, T., Lechner, M.S., Daniel, J.A., et al. (2009). Multiple organ system defects and transcriptional dysregulation in the Nipbl(+/−) mouse, a model of Cornelia de Lange syndrome. PLoS Genet. *5*, e1000650.

Kwak, H., and Lis, J.T. (2013). Control of transcriptional elongation. Annu. Rev. Genet. *47*, 483–508.

Lai, F., Gardini, A., Zhang, A., and Shiekhattar, R. (2015). Integrator mediates the biogenesis of enhancer RNAs. Nature *525*, 399–403.

Langmead, B., and Salzberg, S.L. (2012). Fast gapped-read alignment with Bowtie 2. Nat. Methods *9*, 357–359.

Lengronne, A., Katou, Y., Mori, S., Yokobayashi, S., Kelly, G.P., Itoh, T., Watanabe, Y., Shirahige, K., and Uhlmann, F. (2004). Cohesin relocation from sites of chromosomal loading to places of convergent transcription. Nature *430*, 573–578.

Liu, J., and Krantz, I.D. (2009). Cornelia de Lange syndrome, cohesin, and beyond. Clin. Genet. *76*, 303–314.

Liu, J., Zhang, Z., Bando, M., Itoh, T., Deardorff, M.A., Clark, D., Kaur, M., Tandy, S., Kondoh, T., Rappaport, E., et al. (2009). Transcriptional dysregulation in NIPBL and cohesin mutant human cells. PLoS Biol. *7*, e1000119.

Lower, K.M., Turner, G., Kerr, B.A., Mathews, K.D., Shaw, M.A., Gedeon, A.K., Schelley, S., Hoyme, H.E., White, S.M., Delatycki, M.B., et al. (2002). Mutations in PHF6 are associated with Börjeson-Forssman-Lehmann syndrome. Nat. Genet. *32*, 661–665.

Luo, Z., Lin, C., Guest, E., Garrett, A.S., Mohaghegh, N., Swanson, S., Marshall, S., Florens, L., Washburn, M.P., and Shilatifard, A. (2012). The super elongation complex family of RNA polymerase II elongation factors: gene target specificity and transcriptional output. Mol. Cell. Biol. *32*, 2608–2617.

Machanick, P., and Bailey, T.L. (2011). MEME-ChIP: motif analysis of large DNA datasets. Bioinformatics *27*, 1696–1697.

Malovannaya, A., Li, Y., Bulynko, Y., Jung, S.Y., Wang, Y., Lanz, R.B., O'Malley, B.W., and Qin, J. (2010). Streamlined analysis schema for

high-throughput identification of endogenous protein complexes. Proc. Natl. Acad. Sci. USA *107*, 2431–2436.

Mateo, J.L., van den Berg, D.L., Haeussler, M., Drechsel, D., Gaber, Z.B., Castro, D.S., Robson, P., Crawford, G.E., Flicek, P., Ettwiller, L., et al. (2015). Characterization of the neural stem cell gene regulatory network identifies OLIG2 as a multifunctional regulator of self-renewal. Genome Res. *25*, 41–56.

McLean, C.Y., Bristor, D., Hiller, M., Clarke, S.L., Schaar, B.T., Lowe, C.B., Wenger, A.M., and Bejerano, G. (2010). GREAT improves functional interpretation of cis-regulatory regions. Nat. Biotechnol. *28*, 495–501.

Micallef, L., and Rodgers, P. (2014). eulerAPE: drawing area-proportional 3-Venn diagrams using ellipses. PLoS ONE *9*, e101717.

Michaelis, C., Ciosk, R., and Nasmyth, K. (1997). Cohesins: chromosomal proteins that prevent premature separation of sister chromatids. Cell *91*, 35–45.

Muraki, K., and Tanigaki, K. (2015). Neuronal migration abnormalities and its possible implications for schizophrenia. Front. Neurosci. *9*, 74.

Nativio, R., Wendt, K.S., Ito, Y., Huddleston, J.E., Uribe-Lewis, S., Woodfine, K., Krueger, C., Reik, W., Peters, J.M., and Murrell, A. (2009). Cohesin is required for higher-order chromatin conformation at the imprinted IGF2-H19 locus. PLoS Genet. *5*, e1000739.

Pacary, E., Heng, J., Azzarelli, R., Riou, P., Castro, D., Lebel-Potter, M., Parras, C., Bell, D.M., Ridley, A.J., Parsons, M., and Guillemot, F. (2011). Proneural transcription factors regulate different steps of cortical neuron migration through Rnd-mediated inhibition of RhoA signaling. Neuron *69*, 1069–1084.

Parenti, I., Gervasini, C., Pozojevic, J., Graul-Neumann, L., Azzollini, J., Braunholz, D., Watrin, E., Wendt, K.S., Cereda, A., Cittaro, D., et al. (2016). Broadening of cohesinopathies: exome sequencing identifies mutations in ANKRD11 in two patients with Cornelia de Lange-overlapping phenotype. Clin. Genet. *89*, 74–81.

Phillips-Cremins, J.E., Sauria, M.E., Sanyal, A., Gerasimova, T.I., Lajoie, B.R., Bell, J.S., Ong, C.T., Hookway, T.A., Guo, C., Sun, Y., et al. (2013). Architectural protein subclasses shape 3D organization of genomes during lineage commitment. Cell *153*, 1281–1295.

Rahl, P.B., Lin, C.Y., Seila, A.C., Flynn, R.A., McCuine, S., Burge, C.B., Sharp, P.A., and Young, R.A. (2010). c-Myc regulates transcriptional pause release. Cell *141*, 432–445.

Ramírez, F., Dündar, F., Diehl, S., Grüning, B.A., and Manke, T. (2014). deepTools: a flexible platform for exploring deep-sequencing data. Nucleic Acids Res. *42*, W187–W191.

Rao, Y., Pang, P., Ruan, W., Gunning, D., and Zipursky, S.L. (2000). brakeless is required for photoreceptor growth-cone targeting in Drosophila. Proc. Natl. Acad. Sci. USA *97*, 5966–5971.

Reed, N.P., Henderson, M.A., Oltz, E.M., and Aune, T.M. (2013). Reciprocal regulation of Rag expression in thymocytes by the zinc-finger proteins, Zfp608 and Zfp609. Genes Immun. *14*, 7–12.

Reiner, O., Karzbrun, E., Kshirsagar, A., and Kaibuchi, K. (2016). Regulation of neuronal migration, an emerging topic in autism spectrum disorders. J. Neurochem. *136*, 440–456.

Remeseiro, S., Cuadrado, A., Kawauchi, S., Calof, A.L., Lander, A.D., and Losada, A. (2013). Reduction of Nipbl impairs cohesin loading locally and affects transcription but not cohesion-dependent functions in a mouse model of Cornelia de Lange syndrome. Biochim. Biophys. Acta *1832*, 2097–2102.

Seitan, V.C., Faure, A.J., Zhan, Y., McCord, R.P., Lajoie, B.R., Ing-Simmons, E., Lenhard, B., Giorgetti, L., Heard, E., Fisher, A.G., et al. (2013). Cohesin-based chromatin interactions enable regulated gene expression within preexisting architectural compartments. Genome Res. *23*, 2066–2077.

Skaar, J.R., Ferris, A.L., Wu, X., Saraf, A., Khanna, K.K., Florens, L., Washburn, M.P., Hughes, S.H., and Pagano, M. (2015). The Integrator complex controls the termination of transcription at diverse classes of gene targets. Cell Res. *25*, 288–305.

Stadelmayer, B., Micas, G., Gamot, A., Martin, P., Malirat, N., Koval, S., Raffel, R., Sobhian, B., Severac, D., Rialle, S., et al. (2014). Integrator complex regulates NELF-mediated RNA polymerase II pause/release and processivity at coding genes. Nat. Commun. *5*, 5531.

Suster, M.L., Karunanithi, S., Atwood, H.L., and Sokolowski, M.B. (2004). Turning behavior in Drosophila larvae: a role for the small scribbler transcript. Genes Brain Behav. *3*, 273–286.

Tabata, H., and Nakajima, K. (2001). Efficient in utero gene transfer system to the developing mouse brain using electroporation: visualization of neuronal migration in the developing cortex. Neuroscience *103*, 865–872.

Tekin, M., and Bodurtha, J. (2015). Cornelia de Lange syndrome. Medscape, http://emedicine.medscape.com/article/942792-overview.

Trapnell, C., Roberts, A., Goff, L., Pertea, G., Kim, D., Kelley, D.R., Pimentel, H., Salzberg, S.L., Rinn, J.L., and Pachter, L. (2012). Differential gene and transcript expression analysis of RNA-seq experiments with TopHat and Cufflinks. Nat. Protoc. *7*, 562–578.

van den Berg, D.L., Snoek, T., Mullin, N.P., Yates, A., Bezstarosti, K., Demmers, J., Chambers, I., and Poot, R.A. (2010). An Oct4-centered protein interaction network in embryonic stem cells. Cell Stem Cell *6*, 369–381.

Verrotti, A., Spalice, A., Ursitti, F., Papetti, L., Mariani, R., Castronovo, A., Mastrangelo, M., and Iannetti, P. (2010). New trends in neuronal migration disorders. Eur. J. Paediatr. Neurol. *14*, 1–12.

Verrotti, A., Agostinelli, S., Prezioso, G., Coppola, G., Capovilla, G., Romeo, A., Striano, P., Parisi, P., Grosso, S., Spalice, A., et al. (2013). Epilepsy in patients with Cornelia de Lange syndrome: a clinical series. Seizure *22*, 356–359.

Vierbuchen, T., Ostermeier, A., Pang, Z.P., Kokubu, Y., Südhof, T.C., and Wernig, M. (2010). Direct conversion of fibroblasts to functional neurons by defined factors. Nature *463*, 1035–1041.

Yamamoto, J., Hagiwara, Y., Chiba, K., Isobe, T., Narita, T., Handa, H., and Yamaguchi, Y. (2014). DSIF and NELF interact with Integrator to specify the correct post-transcriptional fate of snRNA genes. Nat. Commun. *5*, 4263.

Yang, P., Shaver, S.A., Hilliker, A.J., and Sokolowski, M.B. (2000). Abnormal turning behavior in Drosophila larvae. Identification and molecular analysis of scribbler (sbb). Genetics *155*, 1161–1174.

Zhang, Y., Liu, T., Meyer, C.A., Eeckhoute, J., Johnson, D.S., Bernstein, B.E., Nusbaum, C., Myers, R.M., Brown, M., Li, W., and Liu, X.S. (2008). Model-based analysis of ChIP-seq (MACS). Genome Biol. *9*, R137.

Zhang, C., Mejia, L.A., Huang, J., Valnegri, P., Bennett, E.J., Anckar, J., Jahani-Asl, A., Gallardo, G., Ikeuchi, Y., Yamada, T., et al. (2013). The X-linked intellectual disability protein PHF6 associates with the PAF1 complex and regulates neuronal migration in the mammalian brain. Neuron *78*, 986–993.

Zuin, J., Franke, V., van Ijcken, W.F., van der Sloot, A., Krantz, I.D., van der Reijden, M.I., Nakato, R., Lenhard, B., and Wendt, K.S. (2014). A cohesin-independent role for NIPBL at promoters provides insights in CdLS. PLoS Genet. *10*, e1004153.

Neuron, Volume *93*

Supplemental Information

Nipbl Interacts with Zfp609 and the Integrator Complex to Regulate Cortical Neuron Migration

Debbie L.C. van den Berg, Roberta Azzarelli, Koji Oishi, Ben Martynoga, Noelia Urbán, Dick H.W. Dekkers, Jeroen A. Demmers, and François Guillemot

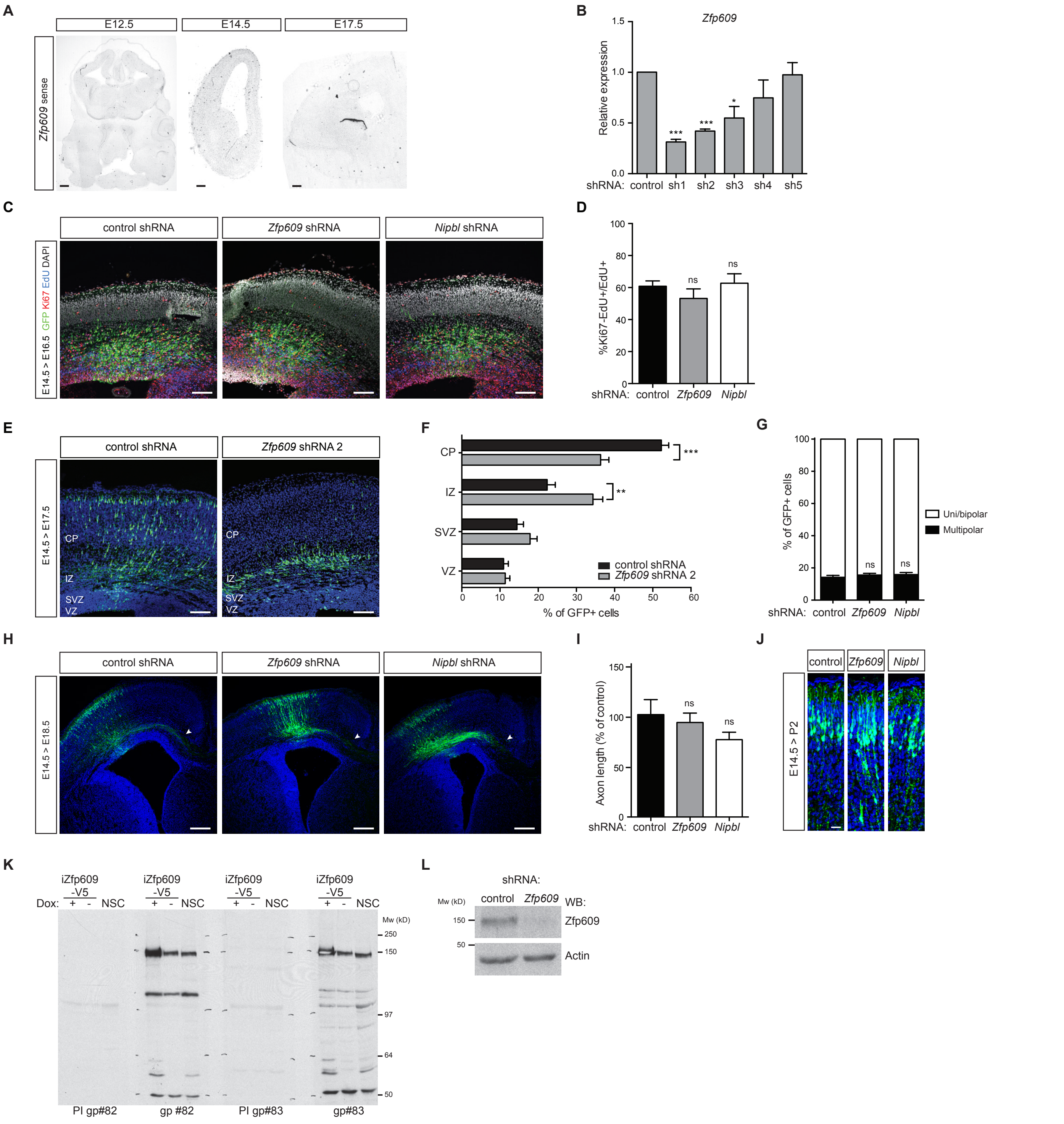

Figure S1, related to Figures 1 and 2.
(A) Composite brightfield images of in situ hybridization with sense Zfp609 probe on mouse embryonic brain sections at indicated developmental stages. (B) Quantitative PCR (qPCR) analysis of transiently transfected P19 cells with *Zfp609* targeting shRNAs. Expression levels were normalized to housekeeping genes (*Tbp* and *Hprt*) and plotted relative to control shRNA transfected cells. * p<0.05, *** p<0.001 unpaired Student's t-test, n=3. (C) Immunohistochemistry on coronal sections of E16.5 mouse embryonic brains electroporated with control, *Zfp609* and *Nipbl* shRNAs and pulse labelled with EdU at E15.5 (D) Quantification of GFP+ cells in (C) showing cell cycle exit rates for different shRNA constructs. Ns, not significant, Student's t-test, n=3 (control) and 4 (*Zfp609* and *Nipbl* shRNA). (E) Immunohistochemistry with GFP antibody on coronal sections of E17.5 mouse embryonic brains electroporated with indicated shRNA constructs. Ventricular (VZ), subventricular (SVZ), intermediate zone (IZ) and cortical plate (CP) are indicated. (F) Quantification of (E) showing percentage of GFP expressing cells in different cortical regions. ** p<0.01, *** p<0.001 unpaired Student's t-test, n=7. (G) Morphological classification of electroporated neurons in cortical plate at E17.5. Ns, not significant, Student's t-test, n=7. (H) Coronal sections of electroporated brains at P0. Arrowheads indicate axons projecting towards the midline. (I) Quantification of axon lengths in primary neuronal cultures of ex vivo electroporated embryonic brains. Ns, not significant, Student's t-test, n=3. (J) Immunohistochemistry with GFP antibody on P2 coronal sections showing apical dendrites in marginal zone. (K) Western blot with pre-immune and anti-Zfp609 sera from two immunized guinea pigs on total cell lysates from control or inducible Zfp609-V5 expressing NS cells. Band at 150 kD represents Zfp609. Doxycycline was added where indicated to induce ectopic expression of Zfp609-V5. (L) Western blot with guinea pig anti-Zfp609 on total cell lysate of control or Zfp609 depleted NS cells demonstrating specificity of the generated antibody. Actin was used as a loading control. Error bars represent SEM. Scale bars represent 200 µm (A,H), 100 µm (C,D) and 20 µm (J).

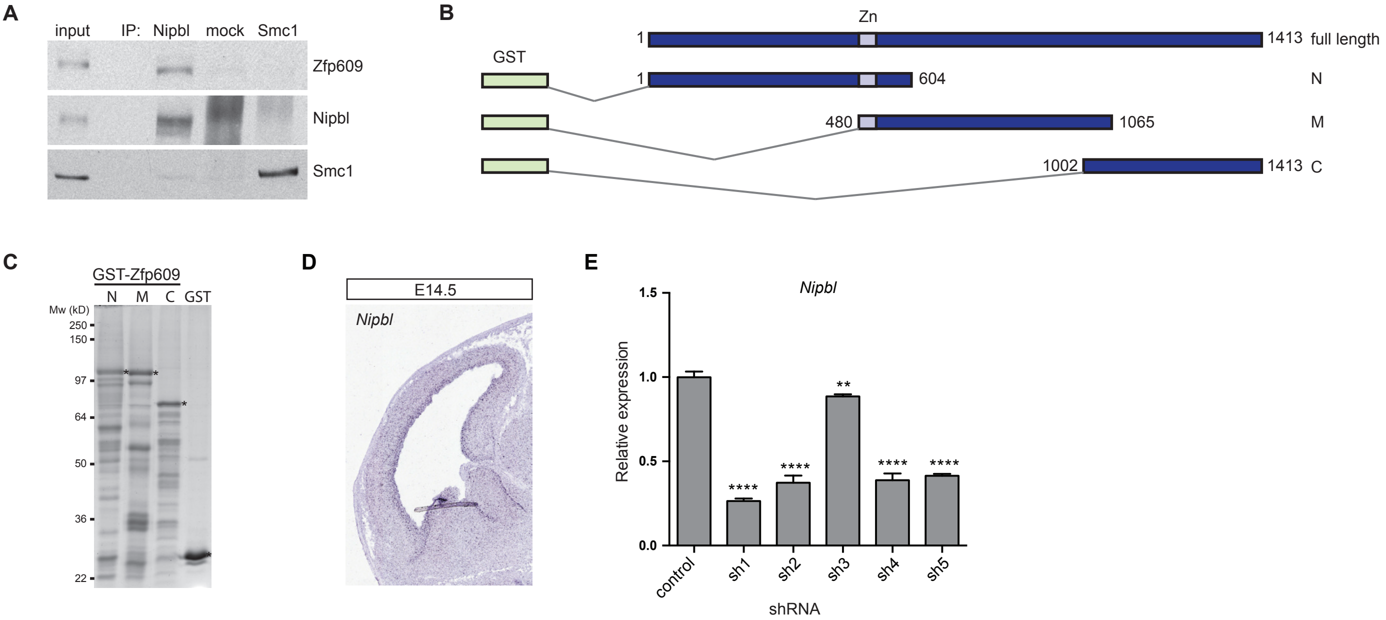

Figure S2, related to Figure 2.

(A) Immunoprecipitates of Nipbl and Smc1 analyzed by Western blotting with the indicated antibodies. Benzonase was added to all samples. Normal rabbit IgG was used as control.

(B) Schematic representation of Zfp609 fragments fused to GST used in GST pull down assays.

(C) Coomassie stained SDS-PAA gel of GST-pull down fractions showing equal loading of GST-fusion proteins, indicated by *.

(D) Genepaint (www.genepaint.org) in situ hybridisation for Nipbl on sagittal section of E14.5 mouse brain showing enriched expression in the ventricular zone.

(E) Quantitative PCR analysis of transiently transfected P19 cells with *Nipbl* targeting shRNAs. Expression levels were normalized to housekeeping genes (*Tbp* and *Hprt*) and plotted relative to control shRNA transfected cells. Error bars represent SEM, ** $p<0.01$, **** $p<0.0001$ unpaired Student's t-test, n=3.

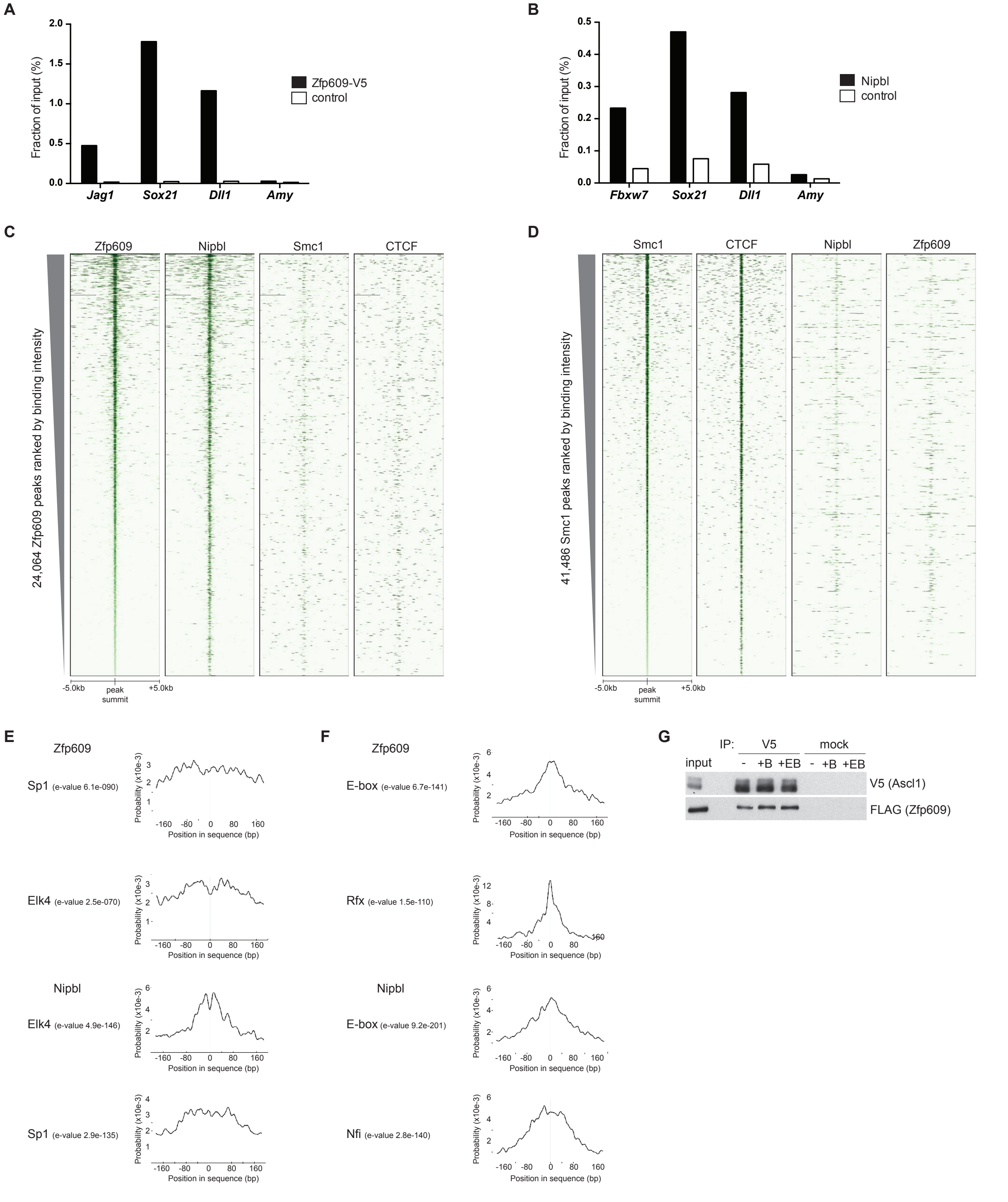

Figure S3, related to Figure 3.

(A) Binding of Zfp609-V5 to *Jag1* (+16 kb), *Sox21* (+6.5 kb) and *Dll1* promoter regions analysed by qPCR of anti-V5 ChIP material. *Amy2a5* promoter region was used as an internal negative control. NS cells not expressing Zfp609-V5 were used as control.

(B) Binding of Nipbl to *Fbxw7* (+125 kb), *Sox21* (+6.5 kb) and *Dll1* promoter regions analysed by qPCR of anti-Nipbl ChIP material. *Amy2a5* promoter region was used as an internal negative control region and rabbit IgG was used as control.

(C) Heatmap of 24,064 Zfp609 bound regions displaying control-normalized mean read counts for indicated factors in a 10 kb window centered around Zfp609 peak summits.

(D) Heatmap of 41,486 Smc1 bound regions displaying control-normalized mean read counts for indicated factors in a 10 kb window centered around Smc1 peak summits

(E) Centrimo enrichment profiles for significantly enriched motifs around proximal Zfp609 (top) and Nipbl (bottom) peak summits.

(F) Centrimo enrichment profiles for significantly enriched motifs around distal Zfp609 (top) and Nipbl (bottom) peak summits.

(G) Western blot analysis with indicated antibodies of V5 immunopreciptates from NS cells ectopically expressing V5-Ascl1 and Zfp609-FLAG. Mouse IgG was used as control. Benzonase (+B) and ethidium bromide (+EB) were added as indicated.

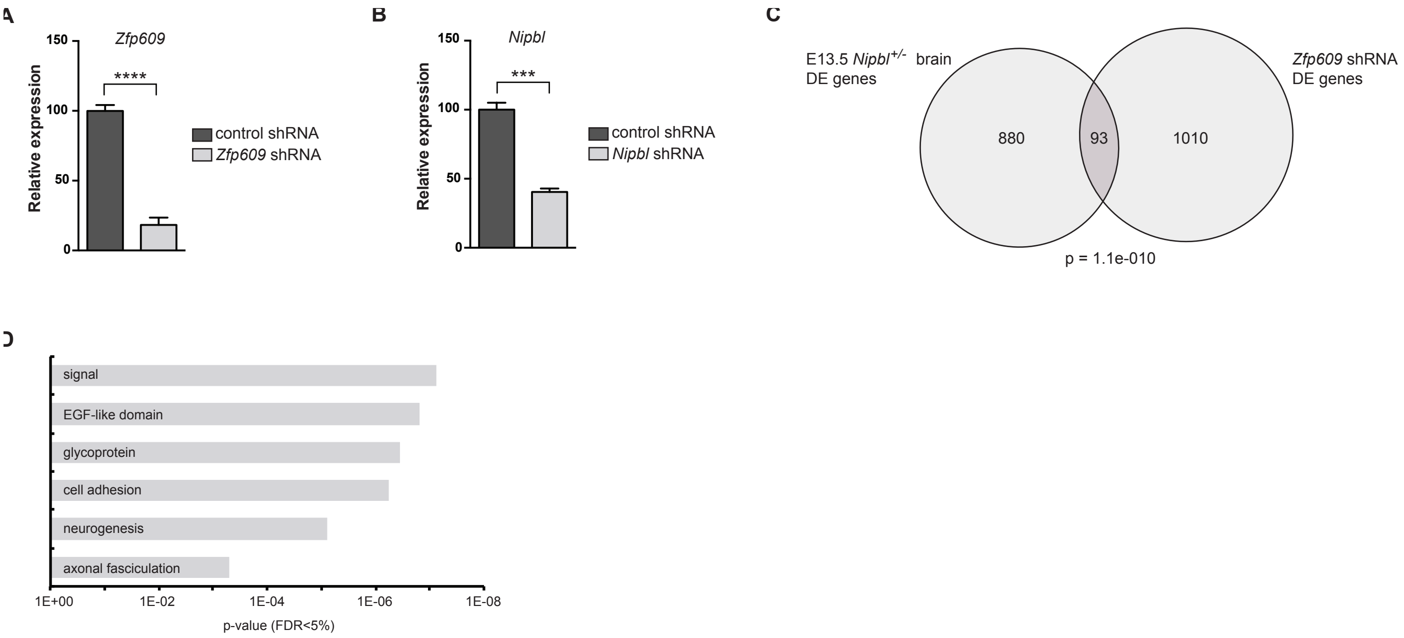

Figure S4, related to Figure 4.

(A) qPCR analysis of NS cells transduced with *Zfp609* or control shRNA expressing lentivirus. *Zfp609* expression levels were normalized to housekeeping genes (*Tbp* and *Hprt*) and plotted relative to control shRNA transduced cells. Error bars represent SEM, ****p<0.0001 unpaired Student's t-test, n=3.

(B) qPCR analysis of NS cells transduced with *Nipbl* or control shRNA expressing lentivirus. *Nipbl* expression levels were normalized to housekeeping genes (*Tbp* and *Hprt*) and plotted relative to control shRNA transduced cells. Error bars represent SEM, ***p<0.001 unpaired Student's t-test, n=3.

(C) Venn diagram showing overlap of differentially expressed (DE) genes between E13.5 *Nipbl*$^{+/-}$ brain and Zfp609 depleted NS cells. P-value from hypergeometric test is shown.

(D) Gene ontology analysis on common DE genes from (C). DAVID p-values are shown, FDR<5%.

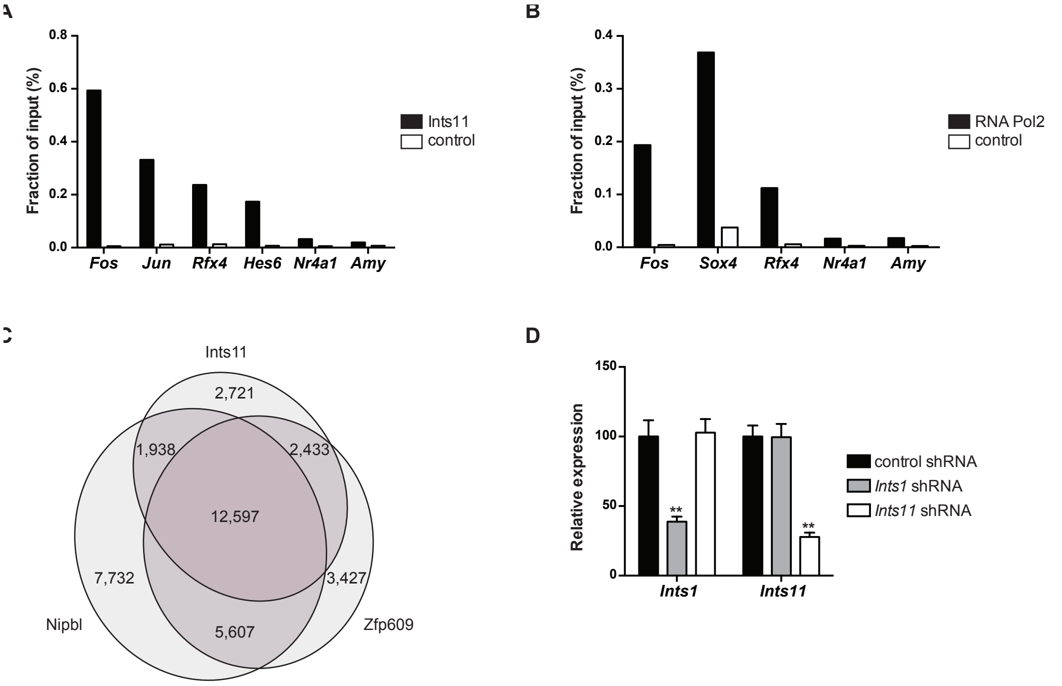

Figure S5, related to Figure 5.

(A) Binding of Ints11 to *Fos*, *Jun*, *Rfx4*, *Hes6* and *Nr4a1* promoter regions analysed by qPCR of Ints11 ChIP. *Amy2a5* promoter was used as internal negative control region. GFP ChIP was used as control.

(B) Binding of RNA pol2 to *Fos*, *Sox4*, *Rfx4* and *Nr4a1* promoter regions analysed by qPCR of Ints11 ChIP. *Amy2a5* promoter was used as internal negative control region. GFP ChIP was used as control.

(C) Venn diagram demonstrating overlap of Ints11, Zfp609 and Nipbl bound regions.

(D) qPCR analysis of NS cells transduced with lentivirus expressing control, *Ints1* or *Ints11* targeting shRNAs. Expression levels were normalized to housekeeping genes (Tbp and Hprt) and plotted relative to control shRNA transduced cells. Error bars represent SEM, $**p<0.01$ Unpaired Student's t-test corrected for multiple comparisons using the Holm-Sidak method, n=3.

Table S1, related to Table 1.
Zfp609-V5 interacting proteins as identified by mass spectrometry in replicate samples of Zfp609-V5 and control purifications.

		Zfp609-V5 #1			Zfp609-V5 #2			control #1			control #2		
**Protein name**	**Accession**	**Mascot**	**emPAI**	**Pept**	**Mascot**	**emPAI**	**Pept**	**Mascot**	**emPAI**	**pept**	**Mascot**	**emPAI**	**pept**
Zfp609	Q8BZ47	4297	11.6	68	4330	26.98	74	388	0.3	9	735	0.53	17
**Cohesin complex**													
Nipbl	Q6KCD5	1577	0.44	33	4332	2.7	93	nd	nd	nd	1082	0.37	28
Smc3	Q9CW03	881	0.55	18	3275	6.76	63	nd	nd	nd	911	0.73	22
Smc1a	Q9CU62	791	0.54	18	3304	7.08	70	nd	nd	nd	1016	0.88	26
Stag2	A2AFF6	228	0.13	5	1291	1.16	27	nd	nd	nd	137	0.09	4
Rad21	Q61550	287	0.35	6	1177	2.96	27	nd	nd	nd	73	0.09	2
Mau2	Q9D2X5	185	0.23	4	626	1.48	14	nd	nd	nd	161	0.26	5
**Integrator complex**													
Ints1	K3W4P2	2259	1.1	47	3560	3.46	78	nd	nd	nd	813	0.59	20
Ints6	Q6PCM2	1592	2.08	29	2076	5.89	43	nd	nd	nd	406	0.57	12
Ints3	Q7TPD0	1494	1.81	27	1933	3.07	37	nd	nd	nd	566	0.5	13
Ints7	Q7TQK1	1365	1.24	23	1679	3.32	31	nd	nd	nd	192	0.2	5
Asun	Q8QZV7	1282	1.82	24	1620	9.79	33	nd	nd	nd	344	0.41	8
Ints5	Q8CHT3	1015	1.01	19	970	1.42	19	nd	nd	nd	316	0.3	7
Ints2	Q80UK8	650	0.34	11	1333	1.31	27	nd	nd	nd	312	0.21	8
Cpsf3l	Q9CWS4	766	1.4	16	971	3.27	21	nd	nd	nd	247	0.32	6
Vwa9	Q8R3P6	521	0.99	11	902	2.2	17	nd	nd	nd	145	0.25	4
Ints8	Q80V86	541	0.47	12	950	1.32	21	nd	nd	nd	199	0.18	6
Ints9	Q8K114	398	0.54	10	760	2.18	19	nd	nd	nd	122	0.14	3
Ints12	Q9D168	410	0.67	7	739	2.68	15	nd	nd	nd	218	0.48	5
**Transcription factors**													
Rfx4	Q7TNK1	584	0.68	13	935	1.92	23	nd	nd	nd	279	0.36	8
Zbtb20	Q8K0L9	762	0.94	13	481	0.6	8	nd	nd	nd	108	0.12	3
**Other**													
Maged1	Q9QYH6	672	0.73	12	975	1.46	20	nd	nd	nd	281	0.3	7
Hspa2	P17156	684	1.06	10	858	1.74	17	nd	nd	nd	nd	nd	nd
Dnaja2	Q9QYJ0	313	0.72	7	311	0.73	7	nd	nd	nd	92	0.23	3
Stub1	Q9WUD1	223	0.66	6	350	1.94	9	nd	nd	nd	nd	nd	nd
Akap8l	Q9R0L7	166	0.16	3	303	0.43	7	nd	nd	nd	nd	nd	nd
Bag5	Q8CI32	253	0.63	6	157	0.36	5	nd	nd	nd	nd	nd	nd
Cnp	P16330	63	0.08	1	265	0.6	7	nd	nd	nd	nd	nd	nd
Setx	A2AKX3	51	0.01	1	189	0.06	5	nd	nd	nd	nd	nd	nd
Mlf2	Q99KX1	103	0.29	2	113	0.57	3	nd	nd	nd	nd	nd	nd
Zcchc11	A2A8R7	54	0.04	2	117	0.07	4	nd	nd	nd	nd	nd	nd
Nabp2	E9Q199	53	0.17	1	96	0.32	2	nd	nd	nd	nd	nd	nd

Mascot score, emPAI score and number of unique, non-redundant peptides for indicated samples as in Table 1. Nd, not detected.

Table S2, related to Experimental Procedures.
Differentially expressed genes identified by RNA-Seq analysis in control, *Zfp609*, *Nipbl*, *Ints1* or *Ints11* shRNA transduced NS cells.

SUPPLEMENTAL EXPERIMENTAL PROCEDURES

Antibody Research Resource Identifiers

Normal mouse IgG Santa Cruz Biotechnology Cat# sc-2025 RRID:AB_737182; normal rabbit IgG Santa Cruz Biotechnology Cat# sc-2027 RRID:AB_737197; Lamin B1 Santa Cruz Biotechnology Cat# sc-6216 RRID:AB_648156 and Santa Cruz Biotechnology Cat# sc-6217 RRID:AB_648158; RNA pol2 Santa Cruz Biotechnology Cat# sc-899 RRID:AB_632359; Smc1 Bethyl Cat# A300-055A RRID:AB_2192467; Nipbl Bethyl Cat# A301-779A RRID:AB_1211232; Int1 Bethyl Cat# A300-361A RRID:AB_2127258; Int11 Bethyl Cat# A301-274A RRID:AB_937779; V5 Thermo Fisher Scientific Cat# R960-25 RRID:AB_2556564; Actin Sigma-Aldrich Cat# A2066 RRID:AB_476693; Vcp Abcam Cat# ab11433 RRID:AB_298039; GFP AbD Serotec Cat# 4745-1051 RRID:AB_619712; GFP Santa Cruz Biotechnology Cat# sc-8334 RRID:AB_641123; GFP Abcam Cat# ab13970 RRID:AB_300798; Ki67 BD Biosciences Cat# 550609 RRID:AB_393778.

Nuclear extract preparation

Control and Zfp609-V5 expressing NS cells were grown to near confluency in 14 cm diameter dishes, scraped in 2 ml ice cold PBS supplemented with 1x complete EDTA-free protease inhibitors (Roche) and spun down at 200g at 4°C. Cell pellet volumes (PV) determined at this point were used as a reference throughout the procedure. Cells were resuspended in 5 PV buffer A (10 mM Hepes pH7.6, 1.5 mM $MgCl_2$, 10 mM KCl), incubated for 10 min on ice, harvested by centrifugation at 1400g and lysed in 2 PV buffer A using 10 strokes with pestle A in a dounce homogenizer. Nuclei were harvested by centrifugation at 1400g and proteins were extracted in 1.5 PV buffer C (20mM Hepes pH7.6, 0.2 mM EDTA, 1.5 mM $MgCl_2$, 420 mM NaCl, 20% glycerol) with 10 strokes of pestle B in a dounce homogenizer. Extracts were incubated for 30 min at 4°C and debris was removed by high speed centrifugation. All buffers were precooled on ice and supplemented with 1x complete EDTA-free protease inhibitors (Roche). Nuclear extracts were diluted two-fold to approximately 100 mM NaCl with buffer C-0 (20 mM Hepes pH7.6, 0.2 mM EDTA, 1.5 mM $MgCl_2$, 20% glycerol).

Mass spectrometric analysis

Gel lanes were cut into 2 mm slices using an automatic gel slicer and subjected to in-gel reduction with dithiothreitol, alkylation with iodoacetamide and digestion with trypsin (Promega, sequencing grade), essentially as described by (Wilm et al., 1996). Nanoflow LCMS/MS was performed on an 1100 series capillary LC system (Agilent Technologies) coupled to an LTQ-Orbitrap mass spectrometer (Thermo) operating in positive mode and equipped with a nanospray source. Peptide mixtures were trapped on a ReproSil C18 reversed phase column (Dr Maisch GmbH; column dimensions 1.5 cm × 100 µm, packed in-house) at a flow rate of 8 µl/min. Peptide separation was performed on ReproSil C18 reversed phase column (Dr Maisch GmbH; column dimensions 15 cm × 50 µm, packed in-house) using a linear gradient from 0 to 80% B (A = 0.1 % formic acid; B = 80% (v/v) acetonitrile, 0.1 % formic acid) in 70 min and at a constant flow rate of 200 nl/min using a splitter. The column eluent was directly sprayed into the ESI source of the mass spectrometer. Mass spectra were acquired in continuum mode; fragmentation of the peptides was performed in data-dependent mode. Peak lists were automatically created from raw data files using the Mascot Distiller software (version 2.1; MatrixScience). The Mascot search algorithm (version 2.2, MatrixScience) was used for searching against the NCBInr database (release NCBInr_20090222; taxonomy: Mus musculus) or the IPI_mouse_database (release 20090924). The peptide tolerance was typically set to 10ppm and the fragment ion tolerance to 0.8 Da. A maximum number of 2 missed cleavages by trypsin were allowed and carbamidomethylated cysteine and oxidized methionine were set as fixed and variable modifications, respectively. The Mascot score cut-off value for a positive protein hit was set to 60, based on at least two peptides. In case of protein identifications with Mascot scores between 50 and 60, or that were based on only one peptide, individual peptide MS/MS spectra were checked manually and either interpreted as valid identifications or discarded. We also show a more quantitative measure of our identified proteins, emPAI score (Ishihama et al., 2005). emPAI score incorporates the number of peptides identified per protein (spectral counts) normalized by the theoretical number of peptides. This is a superior method over just counting the number of identified peptides, because it takes account of the fact that, for the same number of molecules, larger proteins and proteins with many peptides in the preferred mass range for mass spectrometry will generate more observed peptides.

GST pull down

BL21-CodonPlus (DE3)-RP competent cells containing GST expression constructs were grown in the presence of 50 µM $ZnCl_2$ to an OD_{600} = 0.3-0.6. Expression of GST-fusion proteins and GST was induced for 3 hours at 25°C by the addition of 0.4 mM IPTG. Cells were lysed in lysis buffer (25 mM Hepes pH7.6, 5 mM $MgCl_2$, 150 mM NaCl, 10% glycerol, 0.1% NP-40, 50 µM $ZnCl_2$), sonicated and lysates were incubated with glutathione sepharose beads (GE Healthcare) for 2 hours at 4°C. Beads were equilibrated in buffer C-100* and incubated with 200 µl NS cell nuclear extract in the presence of 25 units Benzonase. Bound proteins were analysed by Western blotting.

Chromatin immunoprecipitation

For dual crosslinking NS cells were scraped and washed several times in RT PBS, crosslinked for 45 min at RT with 2 mM disuccinimidyl glutarate (DSG), washed four times with RT PBS and crosslinked for 10 min at RT by the addition of 1/10 volume of 11% buffered formaldehyde solution (50 mM Hepes pH7.6, 100 mM NaCl, 1 mM EDTA, 0.5 mM EGTA, 11% formaldehyde). For RNA polymerase 2 ChIP 1/10 volume of 11% buffered formaldehyde was added directly to the medium. Reactions were quenched for 10 min at RT by the addition of glycine to a final concentration of 125 mM and cells were washed twice with ice cold PBS before being flash frozen in liquid nitrogen. Cell pellets were thawed and incubated for 10 min at 4°C in 5 ml LB1 (50 mM Hepes pH7.6, 140 mM NaCl, 1 mM EDTA, 10% glycerol, 0.5% NP-40, 0.25% Triton X-100) per $1x10^8$ cells. Cell nuclei were collected by centrifugation at 1400g for 5 min at 4°C. Nuclei were incubated for 10 min at 4°C in 5 ml LB2 (10 mM Tris pH8.0, 200 mM NaCl, 1 mM EDTA, 0.5 mM EGTA) per $1x10^8$ cells and collected by centrifugation at 1400g for 5 min at 4°C. Nuclei from $1x10^8$ cells

were then resuspended in 3 ml LB3 (10 mM Tris pH8.0, 100 mM NaCl, 1 mM EDTA, 0.5 mM EGTA, 0.1% Na-deoxycholate, 0.5% N-lauroylsarcosine) and chromatin sonicated to 200 bp fragments in a cooled water bath Bioruptor sonicator (Diagenode). Triton X-100 was added to a final concentration of 1%. For RNA pol2 ChIP cells were sonicated in Pol2 sonication buffer (50 mM Tris-HCl pH 7.5, 140 mM NaCl, 1 mM EDTA, 1 mM EGTA, 1% Triton X-100, 0.1% Na-deoxycholate, 0.1% SDS). 1.5 ml chromatin was precleared with 60 µl protein A or protein G dynabeads equilibrated in LB3 or RNA pol2 sonication buffer and blocked with 0.5 mg/ml BSA. Chromatin was incubated o/n at 4°C with 10 µg antibody and antibody-chromatin complexes were bound to 100 µl equilibrated and BSA-blocked protein A or protein G dynabeads for 1 hour at 4°C. Beads were washed 5 times with RIPA buffer (50 mM Hepes pH7.6, 500 mM LiCl, 1 mM EDTA, 1% NP-40, 0.7% Na-deoxycholate) and once with TE containing 50 mM NaCl. RNA pol2 ChIP was washed 3 times in Pol2 sonication buffer, once in Pol2 sonication buffer containing 500 mM NaCl, once in LiCl wash buffer (20 mM Tris pH 8.0, 1 mM EDTA, 250 mM LiCl, 0.5% NP-40, 0.5% Na-deoxycholate) and once in TE. All lysis and wash buffers were precooled on ice and supplemented with 1x complete EDTA-free protease inhibitors. Chromatin was eluted with 200 µl elution buffer (50 mM Tris pH8.0, 10 mM EDTA, 1% SDS) in a shaking heatblock at 65°C. Crosslinks of 1% input and eluted chromatin were reversed by incubation at 65°C o/n. The elution buffer was diluted two-fold with $T_{50}E_{10}$ and incubated with 0.2 µg/ml RNaseA for 1 hour at 37°C and 0.2 µg/ml proteinase K for 2 hours at 45°C. Samples were extracted twice with phenol:chloroform:isoamyl-alcohol and ethanol precipitated for 30 min at -80°C in the presence of 200 mM NaCl and 30 µg glycogen. DNA pellets were dissolved in 20 µl H_2O, DNA content was quantified by Qubit (Thermo Fisher Scientific) and 2-10 ng was used to prepare sequencing libraries as described in the main text.

Statistical analysis
Statistical tests described in the Figure Legends were performed using Prism software.

Proliferation and axonogenesis
For determination of cell cycle exit rates E14.5 electroporated pregnant mice were injected with 30 µg per gram of bodyweight EdU at E15.5. Embryos were collected at E16.5 and processed as described in the main text. Coronal cryosections were treated with 10 mM Na-citrate pH6.0 for 10 minutes at 90°C and stained with antibodies against GFP (Abcam, ab13970) and Ki67 (BD Pharmingen, #550609). EdU was detected with a Click-iT assay kit (Thermo Fisher Scientific). At least 130 cells were counted per embryo and 3-4 embryos per condition were analysed. To measure the length of axonal projections E14.5 embryonic heads were used for ex vivo electroporation with five 50V pulses at 1s intervals. Brains were dissected and electroporated cortical regions dissociated in L-15 medium supplemented with 10mM Hepes. Cells were seeded on poly-D-lysine and laminin precoated coverslips in Neurobasal medium supplemented with N2, B27, glutamine and Penicillin-Streptomycin. After 2 days in vitro cells were fixed with 4% paraformaldehyde, treated with 10mM Na-citrate pH6.0 at 90°C and stained with GFP and Smi-312 (Biolegend, #837904) antibodies. Axonal lengths of at least 150 cells per experiment were measured using Image J.

Table S3, related to Experimental Procedures.
Targeting sequences of shRNAs used in this study.

Zfp609 shRNA1	GGGAGGAACTGGAACATAA
Zfp609 shRNA2	GCTACAGTGACCAGAGTTA
Nipbl shRNA1	GGAGGGTTATTAAGTTCAA
Nipbl shRNA2	CGCTTCTCAAAGGAAGTTCAA
Ints1 shRNA	GTGCTCCTCAACCACTATA
Ints11 shRNA	GCAGCCATGTTCCAGATTAAA
control shRNA	GCGCGCTTTGTAGGATTCG

Table S4, related to Experimental Procedures.
Primer sequences used for qPCR analysis.

ChIP	Forward	Reverse
Jag1 (+16 kb)	AAGGACAACTCCCTGGAGAA	CTGTTAGTGCCCTGTCTGGA
Sox21 (+6.5 kb)	TTATTTATGCGCCTGCTCCT	GGAATGAATAGCGGTGCAAT
Dll1 (TSS)	GCGTGGCTGTCATTAAGG	GGTGCTGTCTGCATTACC
Fbxw7 (+125 kb)	CAGCTATGTTCCTGCTGTGC	CAACTTCTGCCTGCTTCCTC
Rfx4 (TSS)	TAATGAGTGCCGGGCTAAGG	CCATTGTGGGCCTGTTTTGA
Fos (TSS)	AGCGAGCAACTGAGAAGACT	TCATGGTCGAAGTTTGGGGA
Hes6 (TSS)	GCTCCAGCGTTCTTGGATTT	GCTCCTTGTCCTGGCTCTAA
Sox4 (TSS)	TGCAAGGTAGGAAGCCAAGA	GGTAACCAGCTCCCCTTCTT
Nr4a1 (TSS)	TAGTGGGCGCTTGTTTAGGA	AACCCAGAGTACAGAGTGCC

Amy2a5 (TSS)	CTCCTTGTACGGGTTGGT	AATGATGTGCACAGCTGAA
**cDNA**		
Zfp609	CCTGCTGTGATGATGCAGA	GCCATACGGGGAGAAAGAAT
Nipbl	TGAATCTACCATGCCACTTTGT	CTTCTTCTGTCGCGCTCTTC
Sema3a	TTGCCTGTCTTTTCTGGGGT	AGTCTACTCCGTTCTTCATCCA
Nrp1	ACCTCACATCTCCCGGTTAC	AGAGAAAGGGCCCTGAAGAC
Gabbr2	TCACTCTCTGCCTGGTGTTT	GTAGGCGGTGGTTTTCTGAC
Ints1	GAGAAGCGAGCCATTTCTCC	CTTCAATCTCGTCCAGCAGC
Ints11	AGTGAAATGGTGGGCTACGA	CACGTGGCCTGCATAGTATG
Hprt	AGCCTAAGATGAGCGCAAGT	ATGGCCACAGGACTAGAACA
Tbp	GGGGAGCTGTGATGTGAAGT	CCAGGAAATAATTCTGGCTCA

The following Taqman probes from Applied Biosystems were used: *Plxnd1* Mm01184367_m1, *Actb* 4352933E.

Table S5, related to Experimental Procedures.
Primer sequences used for in situ probe template.

	Forward	Reverse
Zfp609	CCTCCTTATGGCTACAGTGACC	TTCCTTGGGAACAGAGTCCTTA

SUPPLEMENTAL REFERENCE

Wilm, M., Shevchenko, A., Houthaeve, T., Breit, S., Schweigerer, L., Fotsis, T., and Mann, M. (1996). Femtomole sequencing of proteins from polyacrylamide gels by nano-electrospray mass spectrometry. Nature *379*, 466-469.
